# New *Bohaiornis*-like bird from the Early Cretaceous of China: enantiornithine interrelationships and flight performance

**DOI:** 10.7717/peerj.7846

**Published:** 2019-10-25

**Authors:** Luis M. Chiappe, Meng Qingjin, Francisco Serrano, Trond Sigurdsen, Wang Min, Alyssa Bell, Liu Di

**Affiliations:** 1 Dinosaur Institute, Natural History Museum of Los Angeles County, Los Angeles, CA, USA; 2 Beijing Museum of Natural History, Beijing, China; 3 Spanish Royal Academy of Sciences, Madrid, Spain; 4 Department of Biological Sciences, University of Southern California, Los Angeles, CA, USA; 5 Institute of Vertebrate Paleontology and Paleoanthropology, Beijing, China

**Keywords:** Evolution of flight, Jehol Biota, Birds, Enantiornithes, Cretaceous

## Abstract

During the last decade, several *Bohaiornis*-like enantiornithine species—and numerous specimens—have been recognized from the celebrated Jehol Biota of northwestern China. In this paper, we describe the anatomy of another “bohaiornithid” species from the 125 million-year-old Yixian Formation of Liaoning Province, China. The new taxon differs from previously recognized “bohaiornithids” on a number of characters from the forelimb and shoulder girdle. We also provide a new phylogenetic framework for enantiornithine birds, which questions the monophyly of the previously recognized bohaiornithid clade and highlights ongoing challenges for resolving enantiornithine interrelationships. Additionally, we offer the first assessment of the flight properties of *Bohaiornis*-like enantiornithines. Our results indicate that while “bohaiornithids” were morphologically suited for flying through continuous flapping, they would have been unable to sustain prolonged flights. Such findings expand the flight strategies previously known for enantiornithines and other early birds.

## Introduction

During the last three decades, the celebrated Early Cretaceous Jehol Biota of north-eastern China has yielded a rich fauna of enantiornithines (Sereno & Rao, 1992; Zhou, Jin & Zhang, 1992; Zhou, 2002; Hou et al., 2004; Chiappe, Ji & Ji, 2007; Zhou, Clarke & Zhang, 2008; O’Connor et al., 2009, 2011, 2016; Chiappe & Meng, 2016), a diverse clade of Cretaceous birds known worldwide (Chiappe & Walker, 2002). Despite the fact that the interrelationships of these extinct birds continue to be contentious, recent studies have identified a number of distinct Jehol clades including Pengornithidae (Zhou, Clarke & Zhang, 2008; O’Connor et al., 2016), Longipterygidae (Chiappe et al., 2007; O’Connor et al., 2009), and Bohaiornithidae (Hu et al., 2011; Wang et al., 2014), the monophyly of which needs to be further tested. Recent studies on these birds have also begun to explore their aerodynamic properties (Liu et al., 2017, 2019; Serrano et al., 2017, 2018; Chiappe et al., 2014) suggesting that early in their evolutionary history, enantiornithines developed various styles of intermittent flight (e.g., bounding, flap-gliding).

**Figure 1:**
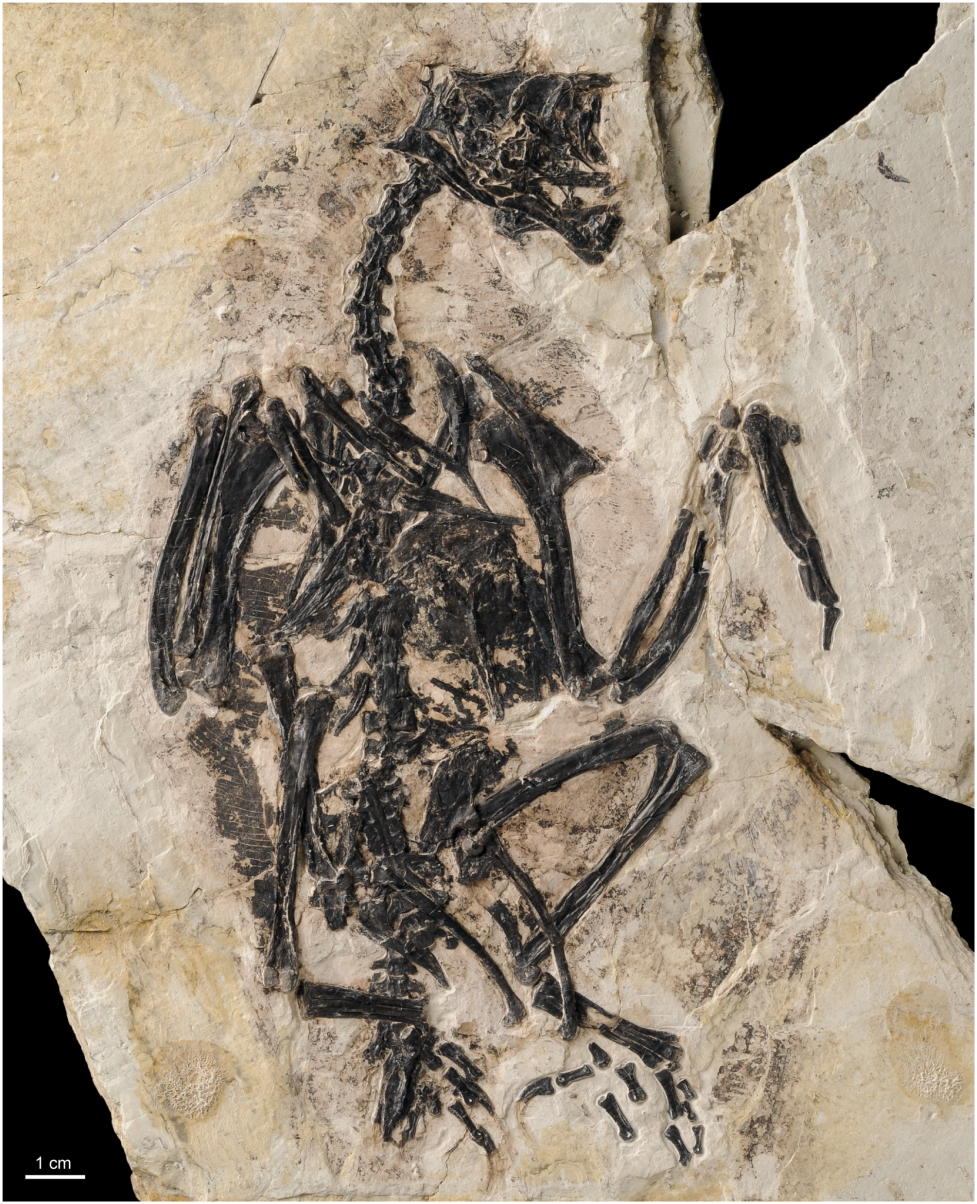
Photo of the holotype of *Gretcheniao sinensis* (BMNHC Ph 829).

“Bohaiornithid” enantiornithines have been recognized only in this decade. *Bohaiornis guoi* was erected by Hu et al. (2011) on the basis of a complete but poorly preserved specimen from the Jiufotang Formation of Liaoning Province (see comment about provenance in Wang et al., 2014). Wang et al. (2014) recognized a series of morphological similarities between *B. guoi* and several other mid-sized enantiornithes from the early Cretaceous of China, and coined the term Bohaiornithidae to name a seemingly monophyletic clade of enantiornithines. Nowadays, as most commonly accepted, Bohaiornithidae is seen as including at least six species of medium-sized birds (Lapwing to Magpie size): *Shenqiornis mengi* (Wang et al., 2010), *Sulcavis geeorum* (O’Connor et al., 2013), *Zhouornis hani* (Zhang et al., 2013), *Longusunguis kurochkini* (Wang et al., 2014), *Parabohaiornis martini* (Wang et al., 2014), and *Fortunguavis xiaotaizicus* (Wang, O’Connor & Zhou, 2014). These species are distributed stratigraphically within an approximately 5-million-year interval (Yixian Formation and Jiufotang Formation) and restricted to the Jehol Biota of north-eastern China. In this study, we describe the anatomy of a new *Bohaiornis*-like taxon (based on BMNH Ph 829) from the 125 million-year-old Yixian Formation (Fig. 1). This new fossil provides information for a novel phylogenetic framework for these birds. Additionally, because the flight style diversity of early birds sheds light on Mesozoic ecosystems, we also assess—for the first time—the flight properties of “bohaiornithids.”

## Methods

The character list and dataset used in the phylogenetic analyses was based on Wang et al. (2014), which in turn was compiled from expanding a series of early bird phylogenies dating back more than 20 years (Chiappe, 1995, 1996a, 2001, 2002). In addition to adding taxa and characters, we made a large number of amendments to the character descriptions, we eliminated some characters, and we modified some scorings contained in the matrix of Wang et al. (2014); the resulting data matrix and character descriptions are included in Appendices 1 and 2, respectively (see File S1 for a Nexus file of the data matrix). All 50 taxa included in the analyses constitute distinct species recognized by unique sets of morphological traits and the scored characters have at least one derived state shared by two or more taxa (i.e., autapomorphic states were excluded). Of the 212 total characters, 148 were described as binary variables; 64 are multistate characters. Of the latter, 24 (characters 1, 3, 8, 23, 39, 44, 48, 51, 52, 68, 77, 84, 134, 143, 149, 162, 173, 180, 181, 183, 187, 190, 195, 200) were treated as additive (or “ordered”), following the recommendation of Slowinski (1993) in which multistate characters are analyzed as additive when they represent morphoclines (i.e., small-medium-large). For purposes of rooting the trees, *Archaeopteryx lithographica* (a taxon usually used as the root for early avian phylogenetic studies) was selected as the root. The data matrix was analyzed using both maximum parsimony and Bayesian inference analyses. The maximum parsimony analysis was conducted using PAUP version 4.0 B10; sequences were added at random and the analysis was set to 100 repetitions. The support of the clades was assessed through a bootstrap analysis, also run in PAUP and using 200 bootstrap replicates. The Bayesian analysis was conducted using the software MrBayes v3.1.2, and it was run for 1,000,000 generations after which the standard deviation of split frequencies was 0.02 (sample frequency every 100th generation, and burnin was chosen to be 2,500).

Data used for the aerodynamic assessment is based on BMNH Ph 829 and other well-preserved “bohaiornithid” specimens (Tables 1 and 2). Anatomical measures were taken by either digital calliper or from high-resolution images using software TPSDig 2.26 (available from http://life.bio.sunysb.edu/morph/). Body mass (*M_b_*) estimates were obtained using multivariate equations derived from modern flying birds and described by Serrano, Palmqvist & Sanz (2015). The values of wingspan (*B*) were also calculated using multiple regressions (Serrano et al., 2017), in which the longest primary feather was incorporated into the models. Such a variable was obtained assuming an isometric change of the length of this feather, which was directly measured in CUGB P1202. Estimates of lift surface (*S_L_*) were obtained from a new multiple regression derived specifically for “bohaiornithids,” following the procedure of Serrano et al. (2017). OLS regressions between *M_b_* and *B* (Table 3) were derived separately for strict flapping and facultative flap-gliding birds. Statistical differences of slopes and intercepts of these regressions were based on 95% bootstrapped confidence intervals. Aspect ratio (AR) and wing loading (WL) were calculated as AR = *B*^2^/*S_L_* and WL = *M_b_*/*S_L_*, respectively; a AR–WL biplot was performed to explore the distribution of extant birds according to their flight strategies (Lindhe Norberg, 2002) and infer the morphofunctional properties of “bohaiornithids.” The dataset of living birds is detailed in the File S2.

**Table 1:** Measurements (in mm) of selected skeletal elements of “bohaiornithid” enantiornithines (R, right side; L, left side). In some cases, the lengths of elements had to be estimated. Averages were used in the statistical analysis whenever two measurements were equally likely to be correct. (1) Holotype of *Gretcheniao sinensis*, BMNH Ph 829; (2) Holotype of *Zhouornis hani*, CNUVB-0903 (Zhang et al., 2013); (3) *Zhouornis hani*, BMNH Ph 756 (Zhang et al., 2014); (4) Holotype of *Parabohaiornis martini*, IVPP V18691 (Wang et al., 2014); (5) *Parabohaiornis martini*, IVPP V18690 (Wang et al., 2014); (6) Holotype of *Bohaiornis guoi*, LPM B00167 (Hu et al., 2011); (7) *Bohaiornis guoi* IVPP V17963 (Li et al., 2014); (8) Holotype of *Shenqiornis mengi*, DNHM D2950 mostly left side (Wang et al., 2010; carpometacarpus length corrected); (9) Holotype of *Sulcavis geeorum*, BMNH Ph 805 (O’Connor et al., 2013); (10) Holotype of *Longusunguis kurochkini*, IVPP V17964 (Wang et al., 2014).

Element/Taxon	(1) *Gretcheniao*	(2) *Zhouornis*	(3) *Zhouornis*	(4) *Parabohaiornis*	(5) *Parabohaiornis*	(6) *Bohaiornis*	(7) *Bohaiornis*	(8) *Shenqiornis*	(9) *Sulcavis*	(10) *Longusunguis*
Scapula	R:∼36.8	40.7	32.4	33.3	?	36.0	36.7	39.3	34.9	34.7
Coracoid length	L: 29.3 R: 29.3	28.4	20.9	21.9	25.6	23.0	22.2	26.2	24.8	24.2
Coracoid width	L: 9.5	∼11.6	11.8	12.3	12.9	12.8	10.8	∼10.7	12.1	12.2
Humerus	L: 49.7 R:∼50.0	50.6	38.3	43.4	46.7	47.0	52.0	46.6	46.5	40.3
Radius	R: 49.2	50.1	35.9	40.3	?	45.4	48.5	45.8	47.7	40.5
Ulna	R: 52.9	53.5	36.9	43.8	?	48.0	52.5	44.8	51.1	43.6
Carpometacarpus	L: 27.9	23.6	19.3	∼20.6	?	∼22.7	23.3	22.5	23.7	∼21.6
Man. Di. I, ph. 1	L: ∼11.2 R:∼9.7	9.1	7.6	8.1	?	9.5	9.0	10.0	9.5	7.1
Man. Di. I, ph. 2	L: ∼4.9	4.6	4.2	3.5	?	4.5	5.1	5.9	4.1	4.5
Man. Di. II, ph. 1	L: 12.3	10.8	8.1	10.2	?	10.8	11.0	11.1	11.3	10.5
Man. Di. II, ph. 2	L: 7.4	8.1	6.4	7.4	?	7.5	7.3	8.7	8.0	6.9
Man. Di. III, ph. 1	L: 6.6	5.6	4.6	5.5	?	5.5	6.7	6.9	6.1	3.4
Pubis	L: 38.8 R: ∼35.9	37.3	31.2	31.0	32.0	33.0	?	33.4	?	29.1
Femur	L: 41.1 R: 43.2	44.5	31.4	36.0	37.5	39.0	42.6	38.8	41.3	35.8
Tibiotarsus	L: 49.6 R: 50.6	52.1	39.3	40.0	45.0	46.0	51.3	?	47.3	41.8
Tarsometatarsus	L: 26.9	26.1	22.5	∼19.5	∼22.0	∼22.5	22.7	24.8	24.9	∼21.4
Pygostyle	∼20.6	17.3	22.4	18.0	21.8	18.5	?	?	19.6	22.8

**Table 2:** Values of body mass (*Mb*), wing span (*B*), lift surface (*S*_L_), aspect ratio (AR), and wing loading (WL) estimated for six bohaiornithid specimens using multiple regressions derived on modern flying birds (see Methods). LCI and UCI indicate lower and upper confidence intervals of the estimates based on the prediction error of the equations.

Species	Specimen	*M_b_* (Kg)	LCI	UCI	*B* (m)	LCI	UCI	*S_L_* (m^2^)	LCI	UCI	AR	LCI	UCI	WL (Kg/m^2^)	LCI	UCI
*Gretcheniao sinensis*	BMNHC-PH 829	0.236	0.190	0.282	0.566	0.552	0.581	0.049	0.043	0.055	6.5	7.1	6.1	4.82	4.44	5.12
*Sulcavis geeorum*	BMNHC-PH 805	0.171	0.138	0.205	0.528	0.514	0.542	0.043	0.038	0.048	6.5	7.0	6.1	3.97	3.66	4.22
*Longusunguis kurochkini*	IVPP V 17964	0.130	0.105	0.155	0.457	0.446	0.469	0.034	0.030	0.039	6.1	6.6	5.7	3.78	3.48	4.01
*Zhouornis hani*	CNUVB-0903	0.227	0.183	0.271	0.559	0.545	0.574	0.048	0.042	0.054	6.5	7.1	6.1	4.73	4.35	5.03
*Zhouornis hani*	BMNHC-PH 756	0.137	0.110	0.164	0.421	0.410	0.432	0.027	0.023	0.030	6.6	7.2	6.2	5.13	4.72	5.45
*Bohaiornithidae* indet.	CUGB P1202	0.088	0.071	0.105	0.327	0.319	0.336	0.017	0.015	0.019	6.2	6.7	5.8	5.09	4.68	5.40

**Table 3:** OLS single regressions analyzing the relationship between body mass (*Mb*) and wingspan (*B*) in two different pools of modern birds. OLS single regressions analyzing the relationship between body mass (*Mb*) and wingspan (*B*) in two different pools of modern birds (log *B* = log *a* + *b* log *Mb*): strict flapping (grouping birds flying through typical continuous flapping and/or bounding) and facultative gliding birds (grouping birds flying through soaring and/or intermittent flap-gliding). 95% confidence intervals (CI) were obtained by bootstrapping (2,000 replicates).

Predominant flight mode	*N*	*R*^2^	Slope (*b*)	CI 95%	Intercept (*a*)	CI 95%
Strict flapping	253	0.922	0.340	[0.331–0.351]	−0.006	[−0.020–0.007]*
Facultative gliding	120	0.951	0.360	[0.347–0.373]	0.132	[0.120–0.143]*

**Notes:**

* Asterisks indicate statistical significance based on non-overlapping of 95% CIs.

Power curves for *Gretcheniao sinensis* were obtained using Flight v. 1.24 software (Pennycuick, 2008). We calculated values of the mechanical power output necessary for flapping flight (*P*_mec_) for a range of velocities (*V_t_*) as the summation of the induced power (*P*_ind_ = 2*k*(*M_b_ g*)^2^/*V_t_*π*B*^2^*ρ*, where *k* is the induced power factor, *g* is the gravity acceleration, and *ρ* is the air density), the parasite power (*P*_par_ = ρ*V_t_*^3^ *S_b_C*_Db_/2, where S_*b*_ is the frontal area of the body and *C*_Db_ is the body drag coefficient), and the profile power (*P*_pro_ = (2*k*(*M_b_ g*)^2^/*V*_mp_π*B*^2^*ρ* + *ρV*_mp_^3^ *S_b_C*_Db_/2)*C*_pro_/(*B*^2^/*S_L_*), where *V*_mp_ is the minimum power speed and *C*_pro_ is the profile power constant). Models assume an elliptical lift distribution across the wingspan; *k* (set at *k* = 1.2) accounts for any deviations from this distribution. *C*_pro_ was fixed at 8.4 to make *P*_pro_ proportional to wing area. Body frontal area was calculated as *S_b_* = 0.00813*M_b_*^0.666^ (Pennycuick et al., 1988), and *C*_Db_ from the equation *S_b_C*_Db_ = 0.01*S_L_* (Taylor, Reynolds & Thomas, 2016). Regarding the environmental factors, *g* was kept at modern values of 9.81 m/s^2^, while *ρ* were established at 1.209 or 1.192 kg/m^3^, values calculated from the ideal gases equation using the values of O_2_ levels (Ward & Berner, 2011) and temperature (Retallack, 2009) for either 120 or 125 Ma (the approximate ages of the Jiufotang and Yixian formations). The models simulated two flying strategies: continuous flapping, and bounding flight in which flapping and ballistic phases lasted equally (i.e., *q* = 0.5; see Pennycuick, 2008). The cruising speed or the speed with the minimum cost of transport, known as maximum range speed (*V*_mr_), was calculated as the tangent to the power curve intersects with the origin of the speed–power plot (Rayner, 1999). The maximum available power generated from the muscles in aerobic metabolism (*P*_av_) was calculated from the oxygen consumption rate (i.e., V_O2_, ml/min), by considering that one ml of O_2_ generates 20.1 J each second (Schmidt-Nielsen, 1997), and assuming a conversion efficiency of 0.2 from the total metabolic power input to the *P*_av_ (Tucker, 1973; Pennycuick, 2008; Bishop & Butler, 2015). Values of *V*_O2_ for *Gretcheniao sinensis* and other “bohaiornithids” were estimated using the allometric equation *V*_O2_ = 160 *M_b_*^0.74^ from Bishop & Butler (2015).

The electronic version of this article in portable document format will represent a published work according to the International Commission on Zoological Nomenclature (ICZN), and hence the new names contained in the electronic version are effectively published under that Code from the electronic edition alone. This published work and the nomenclatural acts it contains have been registered in ZooBank, the online registration system for the ICZN. The ZooBank LSIDs (Life Science Identifiers) can be resolved and the associated information viewed through any standard web browser by appending the LSID to the prefix http://zoobank.org/. The LSID for this publication is: *Gretcheniao sinensis* sp. nov. urn:lsid:zoobank.org:pub:BC33BE8C-7AFA-4D44-8E71-7EC61504AF07. The online version of this work is archived and available from the following digital repositories: PeerJ, PubMed Central, and CLOCKSS.

## Systematic Paleontology

Aves Linnaeus, 1758
Pygostylia Chiappe, 2002
Ornithothoraces Chiappe, 1995
Enantiornithes Walker, 1981
*Gretcheniao sinensis* gen. et sp. nov.

**Holotype.** BMNH Ph 829 (Beijing Museum of Natural History, Beijing, China) (Fig. 1; Table 1). Nearly complete skeleton, largely exposed in ventral view, and contained in a single slab. Faint remnants of plumage are preserved as carbonized material, primarily around the neck and projecting from the distal portion of the left forelimb. A resin cast of the specimen is deposited in the collection of the Dinosaur Institute of the Natural History Museum of Los Angeles County under the number LACM 7917/156630.

**Etymology.** *Gretcheniao* in recognition of Mrs. Gretchen Augustyn and her support to the Dinosaur Institute of the Natural History Museum of Los Angeles County, and the Chinese character (niăo), meaning “bird”; *sinensis* from “sino,” a term generally used in reference to China.

**Geographic and Stratigraphic Provenance.** BMNH Ph 829 (Fig. 1) was recovered from strata belonging to the Early Cretaceous Yixian Formation in Jianchang County, near Huludao City in Western Liaoning, China. The fossiliferous beds of the Yixian Formation have been dated at approximately 125 million-year-old (Swisher et al., 2002; He et al., 2004, 2006), corresponding to the Barremian Epoch of the Cretaceous.

**Differential Diagnosis.** A medium-sized enantiornithine bird sharing the following traits with other “bohaiornithids”: robust rostrum with large subconical teeth that are gently recurved, caudolaterally directed lateral trabeculae of the sternum, and strongly curved and elongated pedal claws. The new species is distinguishable from other *Bohaiornis*-like enantiornithines in having a unique combination of characters including: humeral bicipital area scared by a large cranioventrally facing fossa; slightly tapered omal ends of the furcular rami; ventral tubercle at the convergence of the furcular rami; slender coracoid with a sternal margin that is slightly more than 1/3 the length of the bone and a straight lateral margin; significantly elongated carpometacarpus; and metacarpal II carrying a protuberance on its dorsal surface.

### Anatomical description

**Figure 2:**
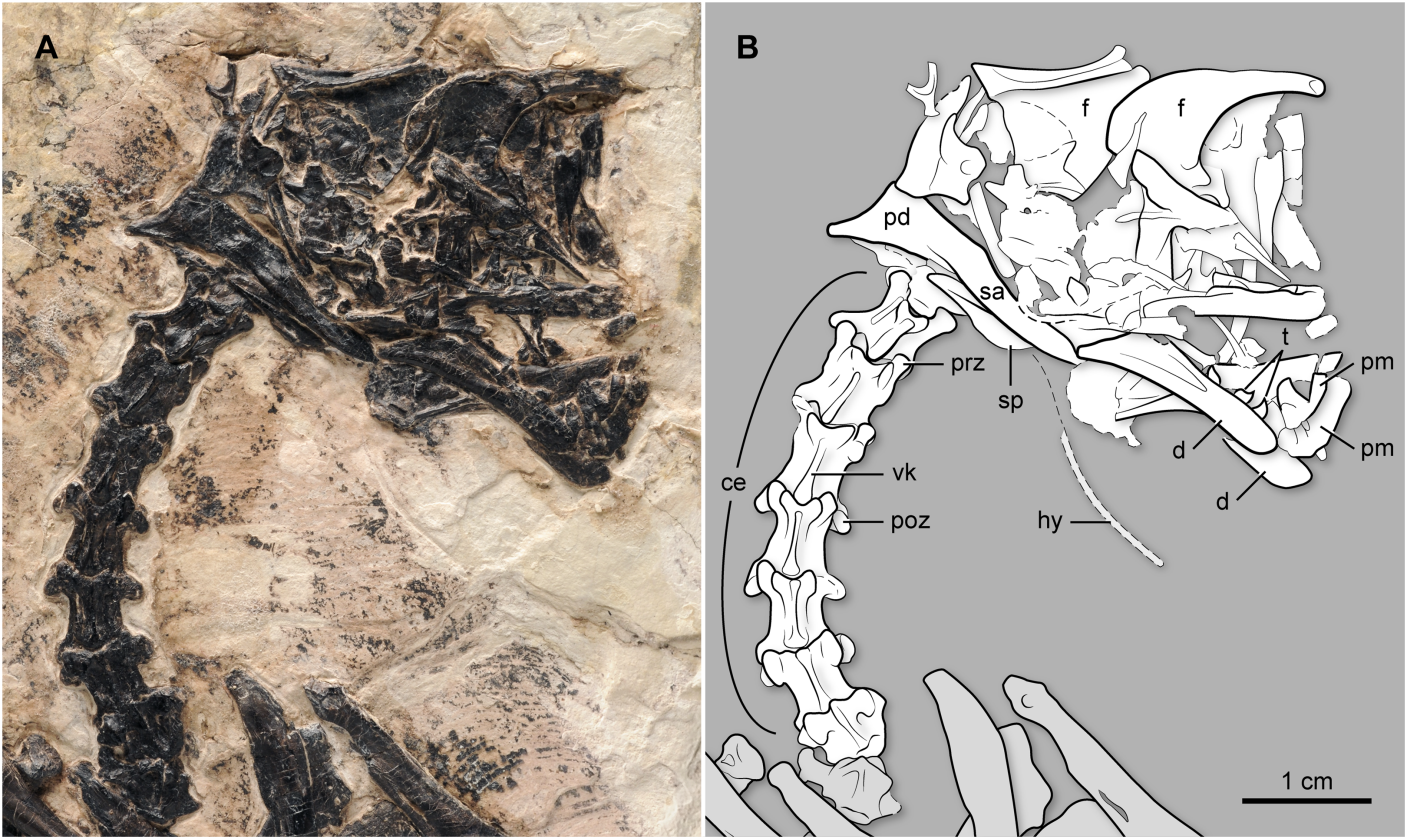
(A) Photo and (B) interpretative drawing of the skull and cervical vertebral series of BMNHC Ph 829. ce, cervical vertebrae; d, dentary; f, frontal; hy, hyoid; pd, postdentary bones; pm, premaxilla; poz, postzygapophysis; prz, prezygapophisis; t, teeth; vk, ventral keel.

**Skull.** Cranial bones are incomplete, largely disarticulated, and jumbled together making their interpretation problematic (Fig. 2). The right frontal is exposed in lateral view, with the caudoventral portion overlain by other elements. The anterior portion of this bone is elongated and nearly straight, proportionally longer than in *Parabohaiornis martini* and more slender than in *Z. hani*. The element is less vaulted dorsally than in other “bohaiornithids,” although it may prove to be a preservational artifact. The ventral margin is concave and thickened. The right dentary is exposed laterally, albeit with its rostral region seemingly broken and displaced in such a way that one piece partially overlaps the other. This bone has some teeth in situ while other teeth are preserved nearby. They have thick and inflated crowns, with recurved and pointy tips. Their enamel appears to show a faint pattern of ornamentation radiating from the tip of the crown. The two dentaries are not fused at their symphysial region. The caudal end of this bone slants caudoventrally, as in other enantiornithines, to form the articulation with the postdentary bones. The right surangular is exposed in lateral view. The dorsal margin is concave along the cranial half but straight distally. A broad, subtriangular bone partially overlapped by the right surangular may represent the splenial, exposed in medial view (Fig. 2).

**Figure 3:**
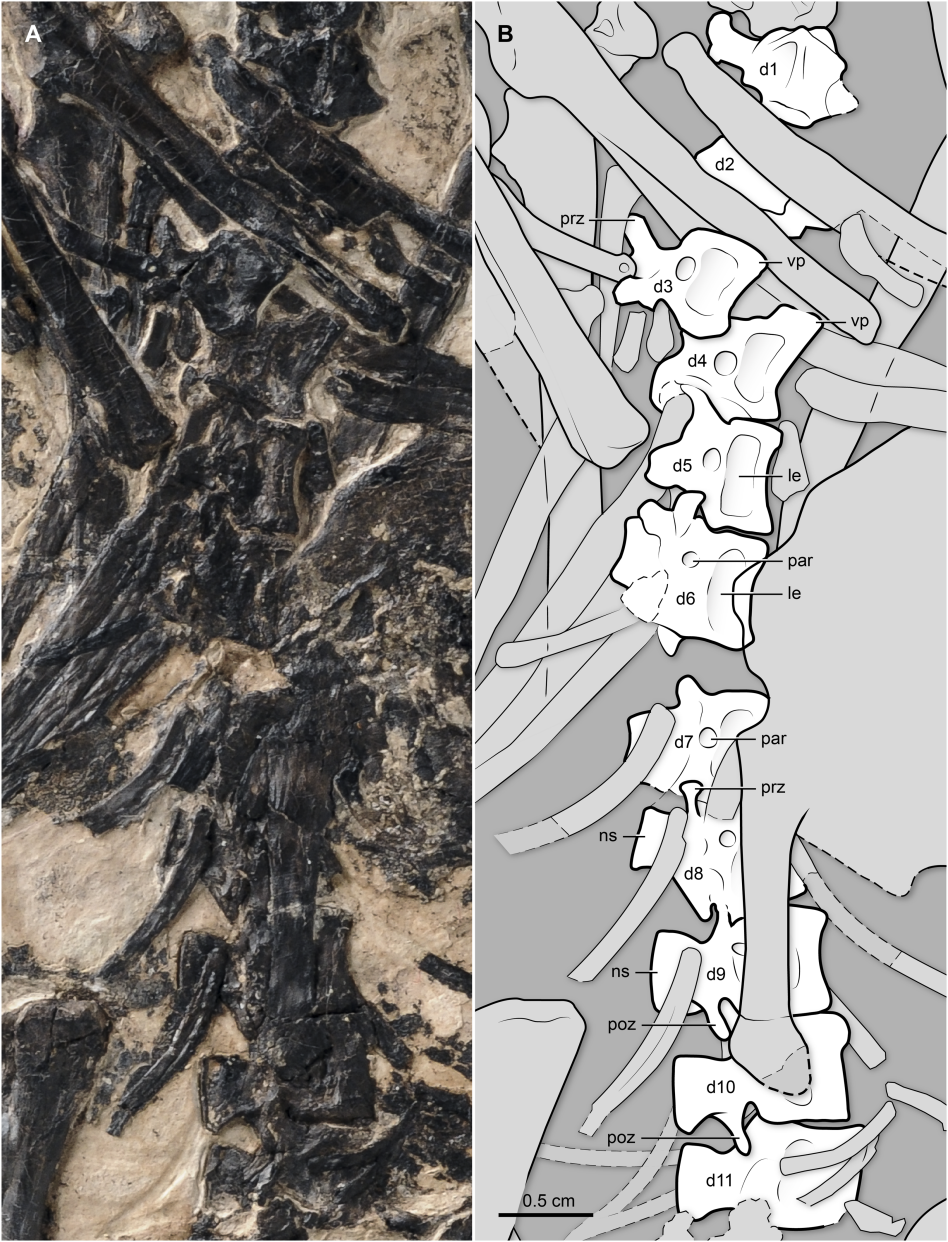
(A) Photo and (B) interpretive drawing of the thoracic vertebral series (dorsal vertebrae) of BMNHC Ph 829. d1–11, dorsal vertebrae 1–11; le, lateral excavation (of centra); ns, neural spine; par, parapophysis; prz, prezygapophysis; poz, postzygapophysis; vp, ventral process.

**Figure 4:**
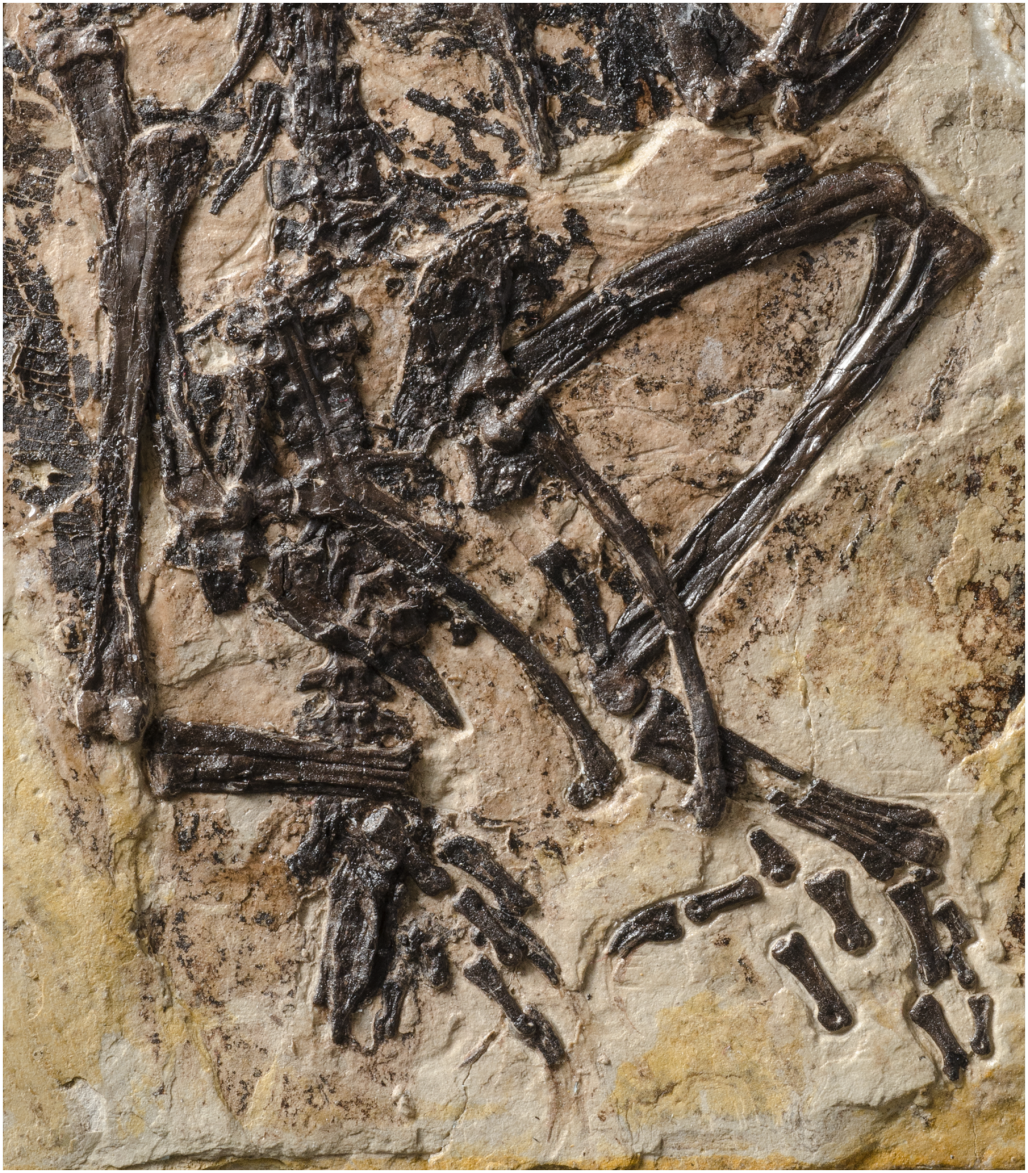
Photo of the pelvis, synsacral and caudal vertebral series, and hindlimb of BMNHC Ph 829. See Fig. 5 for interpretive drawing.

**Figure 5:**
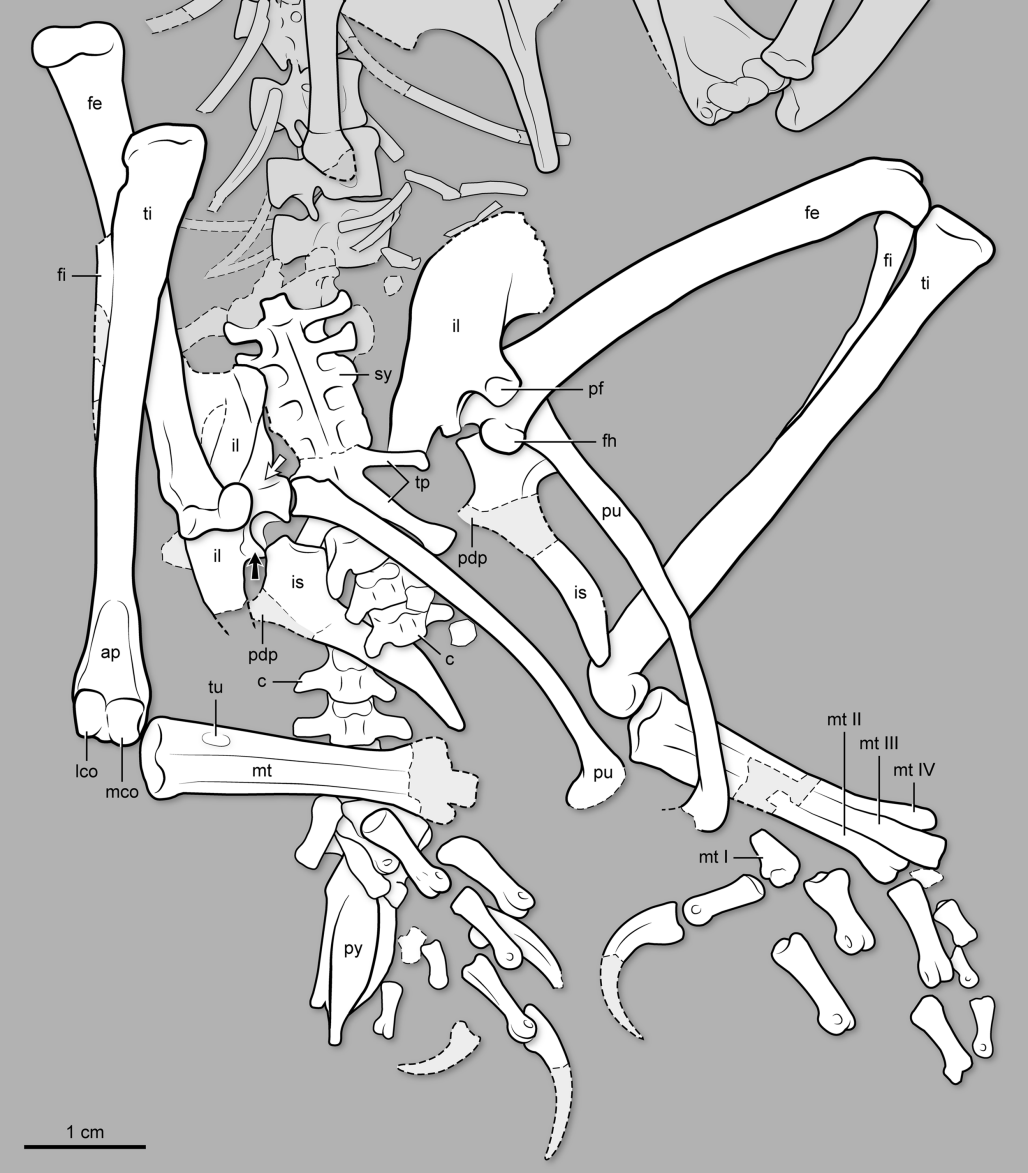
Interpretive drawing of the pelvis, synsacral and caudal vertebral series, and hindlimb of BMNH Ph 829. ap, ascending process of astragalus; c, free caudal vertebrae; fe, femur; fi, fibula; fh, femoral head; il, ilium; is, ischium; lco, lateral condyle of tibiotarsus; mco, medial condyle of tibiotarsus; mt I–IV, metatarsal I–IV; pdp, proximodorsal process of ischium; pf, medial facet for pubis articulation on ilium’s pubic pedicel; pu, pubis; py, pygostyle; ti, tibiotarsus; tp, transverse process; tu, tubercle for the attachment of the *M. tibialis cranialis*; sy, synsacrum. Black arrow points at the antitrochanter of the ilium; white arrow points at the cuppedicus fossa of the ilium.

**Vertebral Column.** There are at least eight vertebrae of cervical morphology not including the atlas, which is not exposed (Fig. 2). They are exposed ventrally. The ventral surface of the centrum forms a distinct keel that becomes thinner caudally. The vertebrae appear to have the incipient degree of heterocoely characteristic of other enantiornithines (Chiappe, 1996a). The preserved third to fifth vertebrae are more elongate than the other cervicals. Eleven articulated thoracic vertebrae are visible, although overlapped to different degrees by other bones (Fig. 3). The first of these is articulated to the cervical series and the last one is proximal to the synsacrum, thus suggesting that the entire thoracic series is represented. The first dorsal has a short ventral process; the process appears to become longer until the fourth dorsal, although it is not fully exposed due to bone overlap. Ventral processes are known for other enantiornithines (e.g., El Brete specimens; Chiappe, 1996a). The thoracic vertebrae are amphyplatyan with expanded neural spines. As the series progresses caudally, the prezygapophyses shorten as the postzygapophyses become longer. The lateral surface of the centra is gently excavated by a shallow, elliptical recess, above which there is a circular and centrally located parapophyses (Fig. 3), as in the majority of the enantiornithines (Chiappe & Walker, 2002). There are at least six vertebrae incorporated in the ventrally exposed synsacrum (Figs. 4 and 5); nonetheless, the synsacrum is incomplete anteriorly. As in *Z. hani*, its ventral surface bears a shallow median sulcus, which is absent in *B. guoi* and *Parabohaiornis martini* (unknown in other “bohaiornithids” due to preservation). The transverse processes define broad, U-shaped depressed areas, which gradually disappear caudally (these depressions are well defined along the anterior half of the synsacrum). The transverse processes of the last two synsacral vertebrae are very long; the last one is strongly angled caudolaterally, as is typical of many enantiornithines (Figs. 4 and 5). The tips of these processes are gently expanded for the attachment to the ilium. At least five free caudals are preserved and exposed ventrally—additional free caudals may be present but the right foot overlapping the area between the last exposed vertebrae and the pygostyle prevents direct observation of these. All caudal vertebrae are similar in morphology; they have short centra, and short and caudolaterally oriented transverse processes (the degree of angulation of these processes decreases caudally) (Figs. 4 and 5). A broad trough excavates the ventral surface of the caudal centra, and their cranioventral margin a facet for the articulation of the haemal arch (none of the latter are clearly identifiable). The articular facets of the first two caudals are amphyplatian, but in the remaining three preserved vertebrae the cranial surface becomes slightly concave, and the caudal one becomes convex (i.e., the vertebrae are slightly procoelous). The pygostyle is partially exposed, its cranial region covered by the right foot. It appears to expose its right lateral and ventral surfaces. The ventral surface exhibits two thin crests—parallel to each other—along most of the length of the bone (Figs. 4 and 5). Similar paired crests project ventrally from the cranial portion of the pygostyle of other enantiornithines (e.g., *Halimornis thompsoni*, *Sinornis santensis*; see Chiappe, Lamb & Ericson, 2002; Sereno, Rao & Li, 2002). The caudal tip, lacking these crests, abruptly tapers into a blunt end.

**Figure 6:**
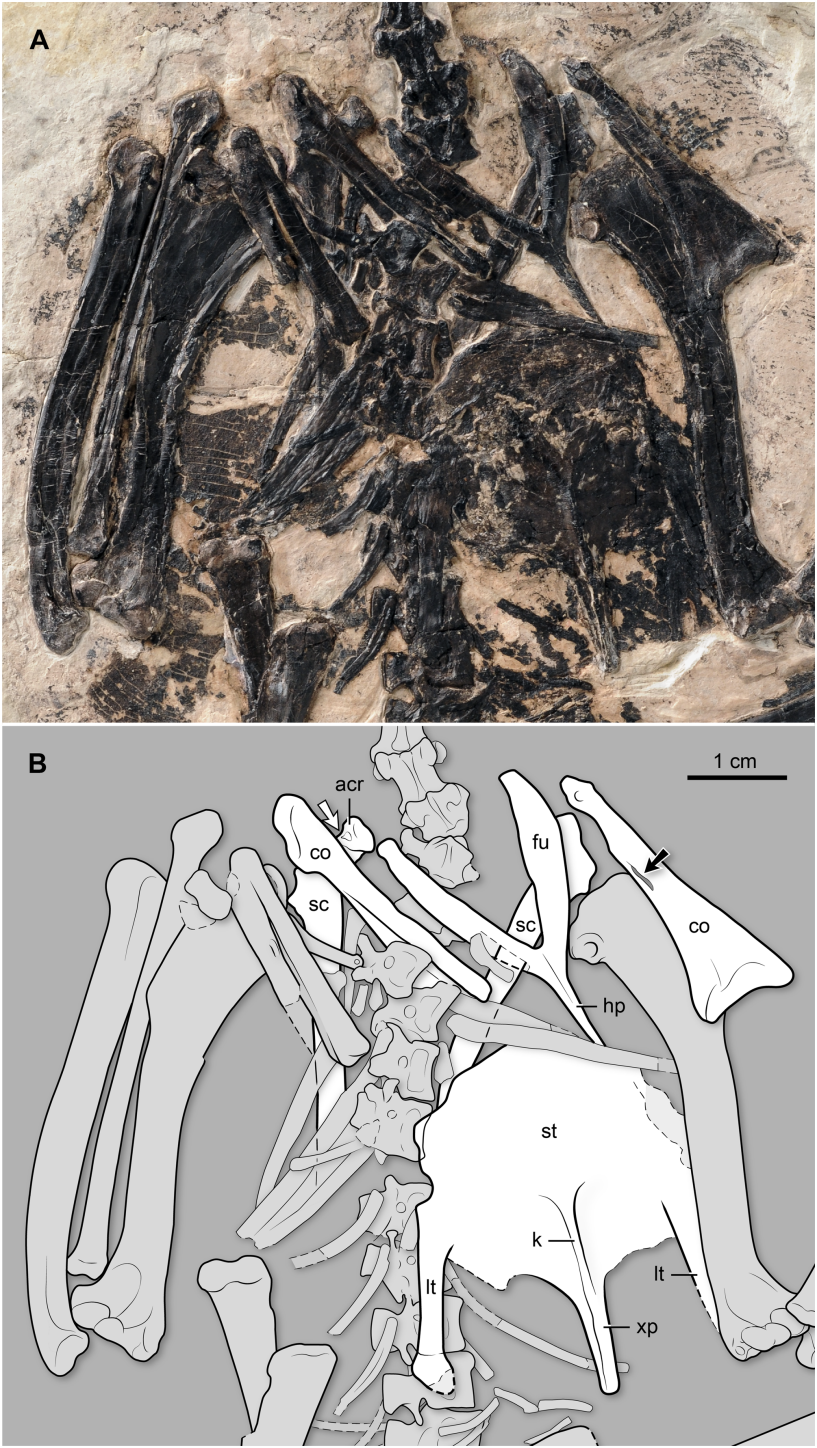
(A) Photo and (B) interpretive drawing of the thoracic region of BMNH Ph 829. acr, acrocoracoid; co, coracoid; fu, furcula; hp, hypocleidium; k, sternal keel (carina); lt, lateral trabecula; sc, scapula; st, sternum; xp, xiphoid process. Black arrow points at the supracoracoid nerve foramen; white arrow points at a fossa denting the ventral surface of the acromion (see description).

**Shoulder girdle and sternum.** Both coracoids are preserved. This bone is slender, and the sternal end is less mediolaterally expanded than in other “bohaiornithids” and most other enantiornithines (Fig. 6). Specifically, the sternal margin width to the shaft length ratio is approximately 0.36 in BMNH Ph 829, smaller than in other “bohaiornithids” (*Parabohaiornis martini*: 0.49; *Longusunguis kurochkini*: 0.50; *B. guoi*: 0.53; *Z. hani*: 0.60; *Shenqiornis mengi*: 0.49). The medial margin is concave, and the lateral margin is generally straight, lacking the strongly convex condition characterizing most other enantiornithines including the *Bohaiornis*-like taxa. Its sternal margin is slightly arched. Above the midshaft, medially, there is a long, slit-like opening for the passage of the supracoracoid nerve (observable on the left element) (Fig. 6). The right element shows that the coracoid is ventrally excavated by a fossa, as is common in other enantiornithines (Chiappe & Walker, 2002). The omal end of this bone slants dorsally; the short acrocoracoid, glenoid, and scapular facet are aligned, a condition also typical of enantiornithines. Both scapulae are preserved, albeit covered by multiple bones. Nonetheless, it is clear that the scapular blade is straight (Fig. 6). Proximally, this bone bears a costolaterally expanded acromion, angled at approximately 130° with respect to the longitudinal axis of the scapular blade. In contrast, the acromion runs nearly parallel to the shaft in other “bohaiornithids” with the exception of *Longusunguis kurochkini*. There appears to be a small fossa on the acromion’s ventral surface (Fig. 6); a similar pit is visible in other enantiornithines (e.g., *Enantiornisn leali* and other El Brete scapulae; see Chiappe, 1996b). The furcula, exposed ventrally, has the Y-shape (approximately 45° of interclavicular angle) and L-shaped cross-section characteristic of enantiornithines (Chiappe & Walker, 2002). Its omal end is gently tapered. The hypocleidium is gently tapered; its ventral surface bearing a faint longitudinal crest. The exposed portion of the hypocleidium extends for a little more than half the length of the rami; while the tip is not exposed, the overall morphology of this process indicates that it was not much longer than its visible portion. At the junction between the hypocleidium and the two rami, there is an oval-shaped and longitudinally oriented, tubercle. Distally, this tubercle connects with a slender, delicate crest that runs down the hypocleidium. In contrast, the corresponding surface is essentially flat in *Z. hani* (see Fig. 6 in Zhang et al. (2014)), and to the best of our knowledge (and taking into account preservational limitations), this trait is also absent in other “bohaiornithids.” The sternum has a rounded cranial margin. The lateral margins are indented by the area for the articulation with the sternal ribs, located just over the base of the lateral trabecula. The lateral trabeculae are slightly angled sideways, as in other “bohaiornithids”; the right lateral trabecular BMNH Ph 829 shows only a minimal distal expansion of its rounded end (the left lateral trabecular of this specimen is partially overlapped by the left humerus). Caudally these processes extend slightly less than the medial xiphoid process. Between the lateral trabeculae and the latter process, the caudal margin of the sternum appears to project into a short median process, although its presence cannot be confirmed due to overlap with carbonized matter. The sternal carina is shallow; it begins around the level of the base of the lateral trabeculae, extending caudally along the ventral surface of the xiphoid process (Fig. 6).

**Figure 7:**
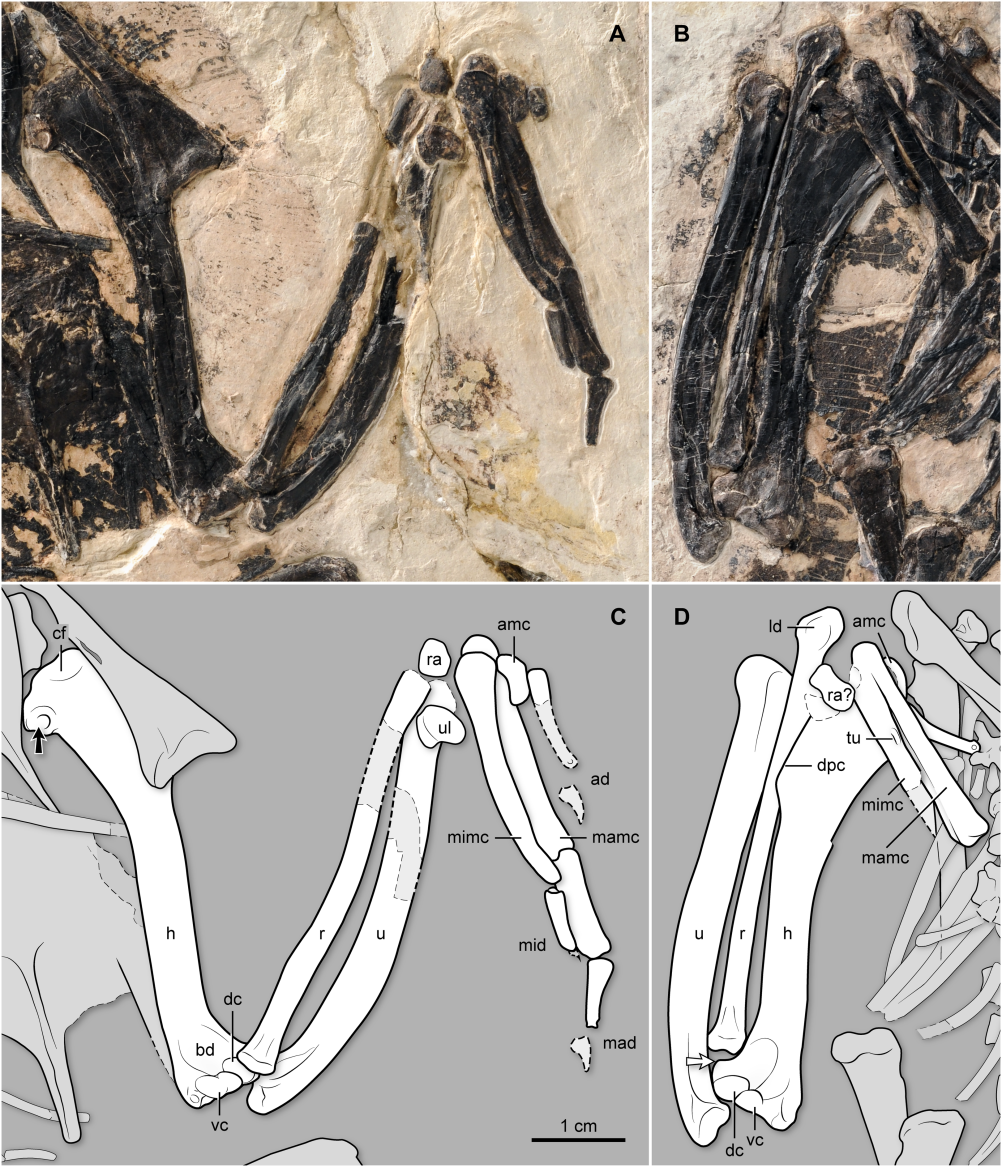
(A–B) Photo and (C–D) interpretive drawing of the left (A, C) and right (B, D) forelimb bones of BMNH Ph 829. ad, alular digit; amc, alular metacarpal; bd, brachial depression; cf, cranial fossa; dc, dorsal condyle of humerus; dpc, deltopectoral crest; h, humerus; ld, ligamental depression; mad, major digit; mamc, major metacarpal; mid, minor digit; mimc, minor metacarpal; r, radius; ra, radiale; tu, tubercle on minor metacarpal; u, ulna; ul, ulnare; vc, ventral condyle of humerus. Black arrow points at the round fossa denting the bicipital crest; white arrow points at the dorsal epicondyle.

**Forelimb.** Both humeri are exposed cranially. They have a slightly sigmoid overall shape. The humeral head is proximally flat, lacking the “double humped” morphology of some other enantiornithines (e.g., *Enantiornis leali*; Chiappe, 1996b). Immediately distal to the head and centered on the cranial surface, there is a shallow, suboval fossa. The deltopectoral crest is weak. The bicipital area is ample; it reveals a broad and round fossa that faces cranioventrally (Fig. 7). The fossa is deep and large, occupying nearly the entire cranial surface of the bicipital crest, a feature otherwise known only in *Linyiornis amoena* among the Early Cretaceous enantiornithines where the corresponding facet is visible (Wang et al., 2016). The distal condyles are oval-to-round. The dorsal one is angled proximodorsally; the ventral condyle, somewhat kidney-shaped, is horizontally oriented (Fig. 7). The two condyles are separated by a clear intercondylar sulcus. The area immediately proximal to the condyles is recessed forming a brachial depression, although there is no presence of a brachial scar. At the level of the proximal margin of the dorsal condyle, the dorsal margin of the humerus swells into a rounded dorsal epicondyle (Fig. 7). The flexor process is poorly developed, as in other “bohaiornithids”; the distal margin of the humerus slants ventrally only slightly. The ulna is straight with the exception of its bowed proximal third (Fig. 7). The ventral cotyla is concave but the dorsal one appears convex as in some other enantiornithines (e.g., El Brete specimens). However, these cotylae are not separated by a groove as is the case in some other enantiornithines (e.g., El Brete specimens). While somewhat crushed, the proximal ventral surface exhibits evidence of a bicipital impression. As most other enantiornithine birds, the ulna lacks any evidence of quill knobs. Its distal end—preserved only on the right wing—shows a semicircular dorsal condyle and a robust carpal tubercle, which is somewhat weathered. The straight radius shows a subtriangular depression on the proximal end of the interosseal surface; in the absence of a bicipital tubercle, it is possible that the radial branch of the *M. biceps brachii* attached to this fossa (it attaches into a pit in some passeriforms; see Ballmann, 1969a, 1969b) and a ligamental depression centered on its distal end (attachment of the *Lig. Interosseum radioulnare distale*, which prevents contact between the radius and the ulna, securing the position of these bones when the wing is folded; Baumel & Raikow, 1993). The right radius shows a longitudinal groove that is probably not an artefact of the bidimensional preservation of the specimen; it is consistent in morphology to similar radial grooves in other enantiornithines (e.g., *Enantiornis leali*, *Pterygornis dapingfangensis*, *Parabohaiornis martini*). Poorly preserved proximal carpals are visible on both wings. The ulnare is heart-shaped as in some other enantiornithines (e.g., *Sinornis santensis*; Sereno, Rao & Li, 2002; Wang & Liu, 2016) as well as in *Bohaiornis*-like enantiornithines. The proximal end of the carpometacarpus is co-ossified, although sutures still delimit the semicircular carpal from its contact with the major and minor metacarpals (right wing) and the alular metacarpal from its contact with major metacarpal (left wing) (Fig. 7). The semilunate carpal has its characteristic pulley-like, proximal articular surface. The subrectangular alular metacarpal has a rounded cranial margin; an extensor process is absent. The major metacarpal is straight and thicker than the minor metacarpal (Fig. 7), which is somewhat bowed ventrally. The intermetacarpal space between these two bones is narrow. As in other enantiornithines (Chiappe & Walker, 2002), the distal end of the minor metacarpal projects further than that of the major metacarpal. The right carpometacarpus, exposed dorsally, shows a distinct tubercle—11 mm from the proximal end of the carpometacarpus—that is developed primarily on the minor metacarpal (Fig. 7D); this tubercle most likely corresponds to the metacarpal protuberance of some modern birds (see Baumel & Witmer, 1993) and the attachment of the *M. extensor carpi ulnaris* (see Vanden Berge & Zweers, 1993). In contrast, this feature is absent in all other “bohaiornithids” described to date.

The partially overlapped, proximal phalanx of the right alular digit and the impressions of the two phalanges of its left counterpart show that the proximal phalanx of this digit was shorter than the proximal phalanx of the major digit, but longer than that of the minor digit. As in other *Bohaiornis*-like enantiornithines, the distal extension of the alular digit approaches the end of the major metacarpal. The major digit contains two non-ungual phalanges (preserved on the left side); the proximal one is almost twice the length of the intermediate one, and much thicker. As in other enantiornithines, the proximal phalanx of the major digit is not expanded craniocaudally. A faint impression of the claw of this digit is visible on the left manus just distal to the end of the intermediate phalanx; based on this impression, the claw of the major digit is assumed to have been smaller than its counterpart on the alular digit. There is only a single phalanx of the minor digit; it is about half the length of the proximal phalanx of major digit and much thinner.

**Pelvic girdle.** The pelvic elements are unfused to one another (Figs. 4 and 5). The ilia are exposed laterally (right) and medially (left). The dorsal margin of the bone is straight; its cranial edge rounded. The preacetabular wing has more than twice the height of the postacetabular wing. Laterally, the preacetabular wing is concave with its ventrocaudal corner projecting laterally. The ventral margin of the preacetabular wing forms a longitudinal trough that develops into a cuppedicus recess, laterally exposed, cranial to the pubic pedicel (Figs. 4 and 5). The pubic pedicel lacks the hooked morphology characterizing *Parabohaiornis martini* and *Longipteryx chaoyangensis* (Wang et al., 2014). The dorsal and ventral margins of the postacetabular wing are convex and concave, respectively, giving this portion of the bone a gently bowed appearance in either lateral or medial view. The end of the postacetabular wing is tapered. The pubic pedicel of the ilium is broad and slightly angled caudally; it projects distally much more than the ischiadic pedicel. The medial surface of the pubic pedicel is recessed, presumably to accommodate the articulation of the proximal end of the pubis (Figs. 4 and 5). The fully perforated acetabulum is small; its horizontal diameter being approximately 10–12% the length of the ilium. On the dorsocaudal corner of the acetabulum, there is a flat and subtriangular antitrochanter (Figs. 4 and 5). The pubes are exposed laterally (right) and medially (left). They are gently bowed, their cranial margin convex. The lateral-medial surfaces of the proximal half are somewhat flattened; those of the distal half are more rounded. This gives the cross-section of this bone a more square appearance proximally and a more rounded contour distally. The pubes end in a boot-like expansion; while the two ends are preserved apart from one another, it is most likely that they contacted, forming a symphysis. The ischium is gently tapered and almost 60% the length of the pubis (Figs. 4 and 5). The lateral surface of the ischium is somewhat convex; the medial one is flatter. The bases of a proximodorsal process are visible on both elements, although the processes are not preserved. The distal end of the ischium is gently curved caudally.

**Hindlimb.** The femur is straight and 85% the length of the tibiotarsus (Figs. 4 and 5). Its rounded head is smooth, lacking a ligamental pit as in other Early Cretaceous enantiornithines (Wang et al., 2016); conversely, the ligamental pit is widely distributed in Late Cretaceous forms (Chiappe & Walker, 2002). The head projects medially at an angle slightly exceeding 90°. The lateral surface of the proximal end of the bone is strongly dented by a posterior trochanter, almost levelled with the head in proximal projection (Figs. 4 and 5). The distal condyles have the combined shape of an hourglass in caudal view. The laterodistal margin lacks the prominent caudal ridge that develops in this region in some other enantiornithines (e.g., *Soroavisaurus australis*; see Chiappe, 1993). The lateral condyle bears a small ectocondylar tubercle (see Chiappe, 1996a), which fails to form a complete tibiofibular crest. The medial condyle is larger than the lateral one, and it extends farther proximally than the latter. The cranial distal end also lacks a patellar groove, as in other enantiornithines (Chiappe & Walker, 2002). The tibiotarsus has no development of cnemial crests, as in most other enantiornithines. Its fibular crest is short but prominent. Distally, the condyles are bulbous and separated by only a faint intercondylar sulcus (Figs. 4 and 5), whereas the sulcus is prominent in *Sulcavis geeorum*; the medial condyle is slightly larger than the lateral one. A distinct ascending process—fused but still delimited by sutures—caps the central surface of the cranial distal surface; it extends for approximately 15% of the length of the tibiotarsus (Figs. 4 and 5). The left fibula is preserved and exposed medially (small fragments of the right fibula are also visible). The medial surface of its proximal surface is slightly concave. The entire bone tapers gradually towards the distal end; at the level of the fibular crest of the tibia, the shaft of the fibula gently expands into an iliofibularis tubercle. Both tarsometatarsi are exposed dorsally. The proximal ends of metatarsals II–IV and distal tarsals are fused to one another; distally, the metatarsals are more loosely connected. The proximal articular surface of the tarsometatarsus is cranially slanted as in some other enantiornithines (e.g., *Soroavisaurus australis*; Chiappe, 1993). The shaft of metatarsal III is thicker than that of metatarsals II and IV; the latter is the thinnest of the three, a condition also found among many enantiornithines (Chiappe, 1993; Chiappe & Walker, 2002). Metatarsal II exhibits a faint longitudinal tubercle for the attachment of the *M. tibialis cranialis*, centered 7.5 mm below the proximal end of the cranial surface (Figs. 4 and 5). Metatarsal III projects more distally than metatarsal II and IV; the latter projects slightly more than metatarsal II. The trochlea of metatarsal II gently bends medially, while that of metatarsal IV splays laterally. The trochlea of metatarsal III is similar in width to that of metatarsal II and similarly ginglymus. That of metatarsal IV is much thinner, bearing only a rounded articular facet. Metatarsal I is short and subtriangular; its medial surface carries a well-demarcated and rounded articular facet for the proximal phalanx of digit I. The latter phalanx is longer than its metatarsal. The claw of this digit is somewhat recurved. The remaining pedal phalanges are largely disarticulated and somewhat scattered. In all, they match the morphology and digital formula (2-3-4-5-X) of enantiornithines. The two non-ungual phalanges, displaced close to the left hallux, most likely represent the proximal and the penultimate phalanges of digit II (Figs. 4 and 5). If this interpretation is correct, digit II is stouter than other digits, a character considered as diagnostic of Bohaiornithidae (Wang et al., 2014). Lateral to digit II, two non-ungual phalanges are preserved partially disarticulated from each other, and we interpret them as the proximal two phalanges of digit III. Three short, delicate non-ungual phalanges that are aligned proximodistally probably belong to digit IV with reference to articulated feet seen in other enantiornithines. The distal phalanx is much longer than the preceding two; in most enantiornithines (O’Connor et al., 2011; Sereno, Rao & Li, 2002; Wang et al., 2014), the proximal three phalanges of digit IV are subequal in length and much shorter than the penultimate phalanx of the same digit. Following this, it is likely that these three phalanges represent the second through fourth phalanges of digit IV. A fragment, preserved between the trochlea of metatarsal III and the proximal phalanx of digit II, is likely to be the proximal phalanx of digit IV. Two other claws preserved—presumably corresponding to digits II and III—appear to match the long and rather strait morphology of other “bohaiornithids” (Wang et al., 2014), although none of them are completely preserved.

## Results and Discussion

**Figure 8:**
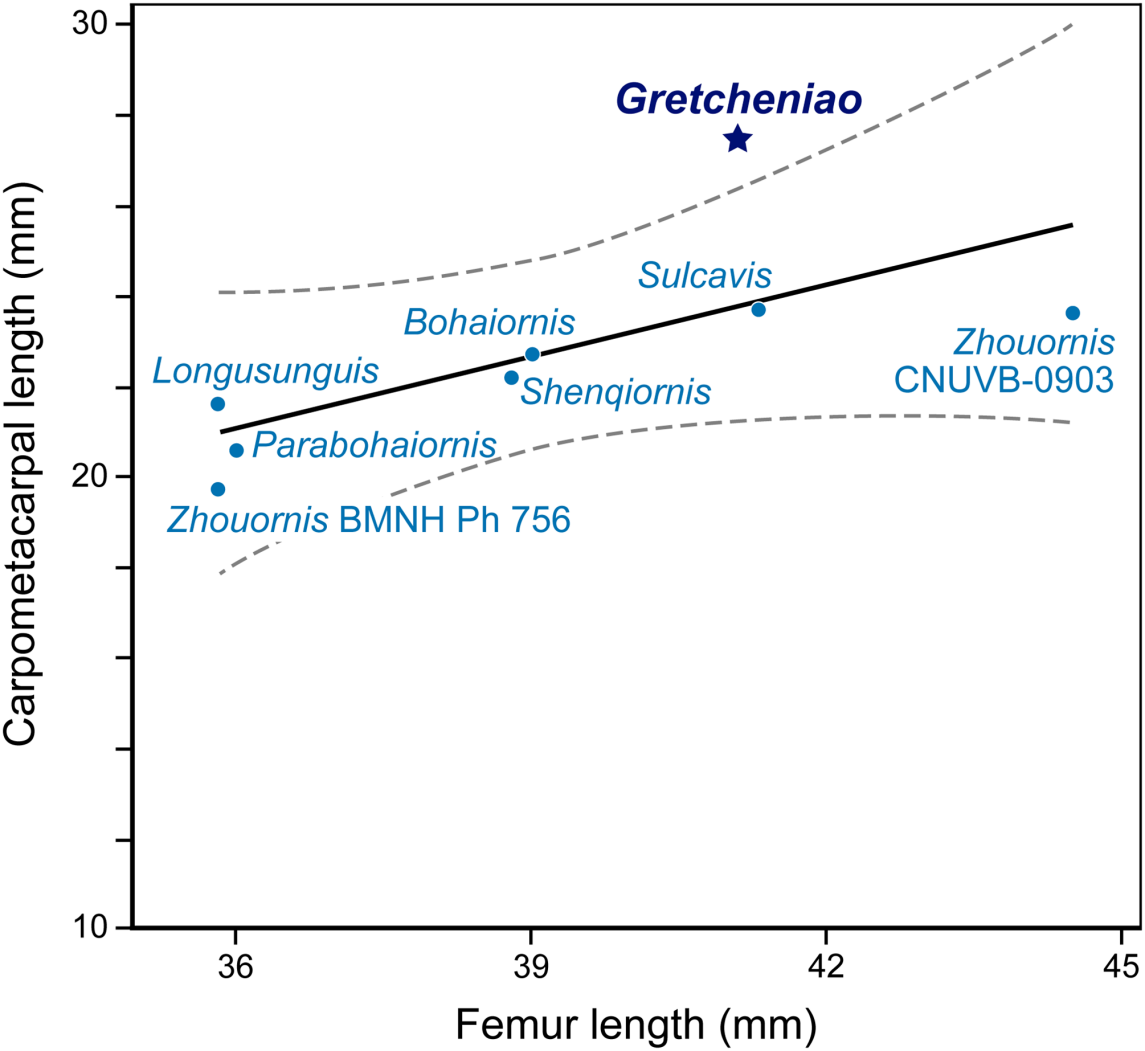
A simple regression analysis of the relative lengths of the carpometacarpus versus femur of different *Bohaiornis*-like enantiornithines. BMNH Ph 829 falls outside the 95% confidence interval, showing the proportionally long carpometacarpus characteristic of *Gretcheniao sinensis*.

The identification of BMNH Ph 829 as an enantiornithine is supported by the presence of characters that are consistently interpreted as synapomorphies of this clade: thoracic vertebrae bearing centrally located parapophyses, a Y-shaped furcula with laterally excavated rami, a radius that bears a longitudinal groove on its interosseous surface, a femur with a prominent posterior trochanter on its proximolateral margin, a relatively thin metatarsal IV, and a long metacarpal III that projects more distally than metacarpal II (Chiappe & Walker, 2002). BMNH Ph 829 also shows a number of traits that are typical of “bohaiornithids”: the generally robust skeleton, a strong rostrum (reflected by the thick dentary), robust teeth that are subconical and slightly recurved, a sternum with caudolaterally oriented lateral trabeculae, a tapered pygostyle lacking an abrupt distal constriction, and elongated pedal claws (Wang et al., 2014). However, BMNH Ph 829 shows features that differentiate it from all other “bohaiornithids.” Notably, the coracoids are unusually long and slender, with a straight lateral margin (Fig. 6). The manus is more robust and relatively longer than in most other “bohaiornithids” (Fig. 8; File S3) as is shown by the fact that the carpometacarpus is the longest of any “bohaiornithid” despite the humerus being only third in size among the known species (Table 1). In addition, BMNH Ph 829 can be further differentiated from other “bohaiornithids” by a unique combination of traits (see Diagnosis). The overall size and skeletal maturation of BMNH Ph 829 indicate that these differences are not ontogenetic. In all, this evidence warrants the erection of a new species, *Gretcheniao sinensis*.

**Figure 9:**
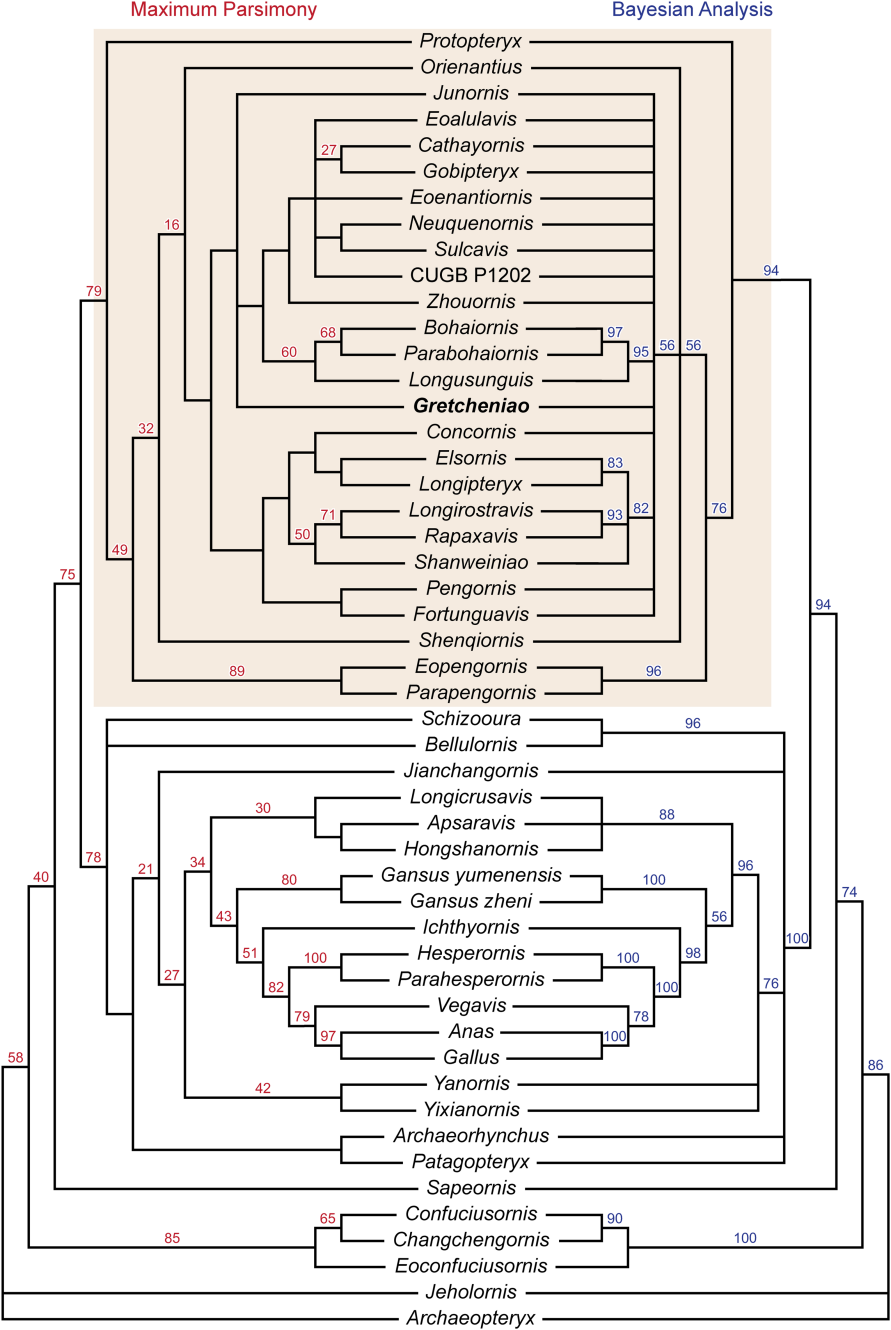
Phylogenetic tree resulting from the maximum parsimony analysis, bootstrap values are shown at the nodes. Phylogenetic tree resulting from the Bayesian analysis, posterior probabilities are shown at the nodes. Shade box highlights the enantiornithine clade.

The results of our phylogenetic analyses show significant differences with those of Wang et al. (2014). The maximum parsimony analysis produced 94 equally parsimonious trees (858 steps), and taken together with the bootstrap support values and Bayesian analysis, our analyses show a general lack of clade support within Enantiornithes (Fig. 9). This is exemplified by the collapse of most clades in the Bayesian analysis, and low bootstrap support for many of the same clades in the parsimony analyses. Our conclusions further highlight the need for caution when drawing phylogenetic inferences on the admittedly contentious tree of enantiornithine birds. However, the analyses do confirm a number of broad conclusions. In agreement with a number of other phylogenetic proposals (Zhou, Clarke & Zhang, 2008; Hu, Zhou & O’Connor, 2014; Wang & Lloyd, 2016), *Protopteryx fengningensis* is resolved as the most basal taxon within Enantiornithes, with forms like *Elsornis keni* in a more derived position (*contra*, e.g., Wang et al., 2014). Pengornithidae may also have a relatively basal position within Enantiornithes, as it has been proposed by other phylogenetic analyses (Zhou, Clarke & Zhang, 2008; Wang & Lloyd, 2016; Wang et al., 2016), but this lacks support in the Bayesian analysis and needs to be investigated further. There is no support for Longipterygidae as previously defined (Wang et al., 2014), but the results of both analyses show strong support for a larger clade including these forms. This encompasses the long-snouted taxa *Longipteryx chaoyangensis*, *Rapaxavis pani*, *Longirostravis hani*, and *Shanweiniao cooperorum* as well as *Concornis lacustris* and *Elsornis keni* for which skulls are unknown. Most importantly for the present study, there is support for the monophyletic grouping of *B. guoi*, *Parabohaiornis martini*, and *Longusunguis kurochkini* (60% bootstrap support, 95% posterior probability). These taxa were previously included in Bohaiornithidae (Wang et al., 2014). However, “bohaiornithids,” as previously defined, also include a number of similar fossil birds that may not form a monophyletic taxon. The six species typically considered as “bohaiornithids” are not clustered in a single monophyletic group. The similarities of these forms, which could be said to include the morphology of *Gretcheniao sinensis*, point to a mix of shared symplesiomorphies and synapomorphies, and may be interpreted as an evolutionary grade (similar to “iguanodontid” ornithischians, “prosauropod” sauropodomorphs, and “pelycosaur” synapsids), which shares an overall morphology, and perhaps ecological niche, but do not constitute a monophyletic taxon. As such, the species typically considered as “bohaiornithids” appear to be relatively basal taxa closely related to *Eoenantiornis buhleri* and leading to the more derived forms such as *Cathayornis yandica* and *Gobipteryx minuta*. Otherwise, the relationships within Enantiornithes are for the most part unresolved (Fig. 9), reflecting once again the challenge of untangling the interrelationships of this clade.

**Figure 10:**
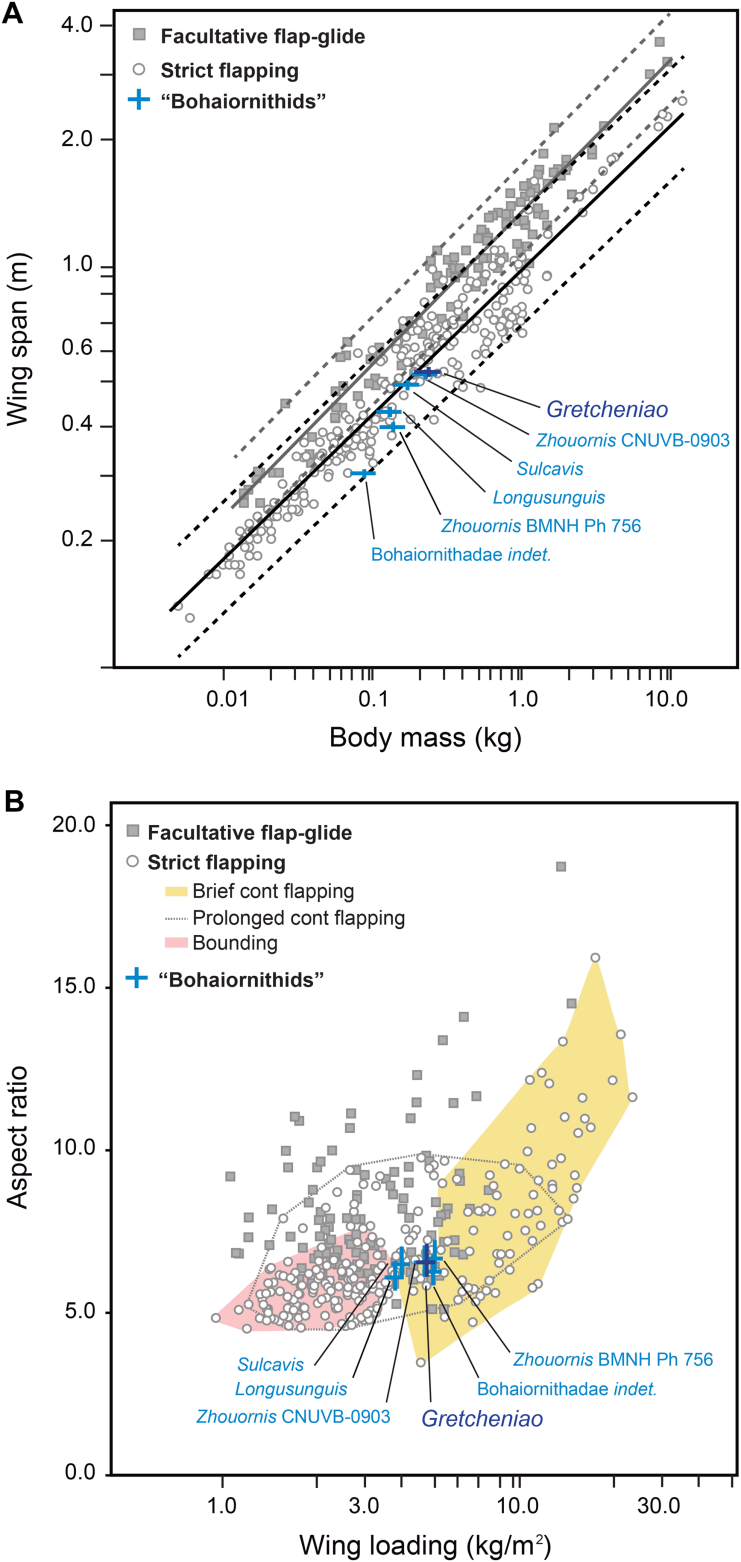
Flight capacity of six “bohaiornithid” specimens, represented in the plots by blue crosses. The length of the bars in the crosses indicate the confidence intervals for the estimation of the fossils, given in Table 2. (A) Relationship between wingspan and body mass in *Bohaiornis*-like enantiornithines and modern flying birds. The black and grey lines correspond to the slopes of the OLS regressions derived for predominantly soaring and predominantly flapping birds, respectively; regression coefficients are shown in Table 3. Dashed lines mark the corresponding confidence intervals for the regression lines based on the 95% of the individuals. This evidence indicates that the “bohaiornithids” were suited for strict flapping flight. (B) Morphospace of the flight modes of extant birds (only basic modes of strict flapping flight are graphically delimited) defined by the relationship between wing loading (WL) and aspect ratio (AR). This evidence indicates that within strict flapping flight, “bohaiornithids” are best suited for brief continuous flapping.

The flight capacity of BMNH Ph 829 and other “bohaiornithids” can be inferred from their well-preserved skeletons. Using Serrano, Palmqvist & Sanz (2015) and Serrano et al. (2017) quantitative models, we obtained values for body mass (*M_b_*), wing span (*B*), lift surface (*S_L_*), AR, and WL of BMNH Ph 829 and other five “bohaiornithid” specimens (Table 2). Within the range of *M_b_* (130–236 g) and *B* (327–566 cm) estimated for these birds, the Magpie-sized BMNH Ph 829 was the largest (i.e., *M_b_* = 236 g; *B* = 566 cm). These estimates indicate that *Bohaiornis*-like enantiornithines had short wings with respect to their body masses; a configuration observed in most modern birds that use strict flapping as their main flight modality (Fig. 10A). For example, the proportions of *Gretcheniao sinensis* approach that of the Cardinal Lory (*Chalcopsitta cardinalis*) and the Watercock (*Gallicrex cinerea*). Although there is some overlap, strict flapping birds tend to have wings significantly shorter than those birds with facultative flap-gliding flight (Tables 2 and 3; Fig. 10A)—the long wings of the latter are an adaptation for efficient gliding (Meseguer & Sanz-Andrés, 2007; Pennycuick, 2008). Thus, the fact that *B* estimates of the fossils lie within the range of strict flapping birds, and under the lower confidence interval of the facultative gliders, indicates that “bohaiornithids” were suited to fly through strict flapping but not through facultative glides. Birds that fly through strict flapping include those that use bounding, hovering (exclusive of hummingbirds and therefore, excluded from this study), and continuous flapping—among the latter, there are those that can fly for long distances (e.g., shorebirds, pigeons, many ducks) and others that spend little time flying (brief fliers such as divers and land fowl). The values of *AR* (6.1–6.6) and *WL* (3.78–5.13 Kg/m^2^) estimated for “bohaiornithids” (see Table 2) are also consistent with a continuous flapping flight. *Gretcheniao sinensis* and its “bohaiornithid” counterparts score in a region of the AR–WL morphospace where brief flyers and prolonged flyers overlap (Fig. 10B). For instance, the values of *Gretcheniao sinensis* approach those of the Common Quail (*Coturnix coturnix*) and Coconut Lorikeet (*Trichoglossus haematodus*). Note that *Longusunguis kurochkini* and *Sulcavis geeorum* are also close to bounding flyers. The low AR shown by “bohaiornithids” could have allowed rapid take-offs as this type of wing maximizes the thrust during slow flight (Swaddle & Lockwood, 2003).

**Figure 11:**
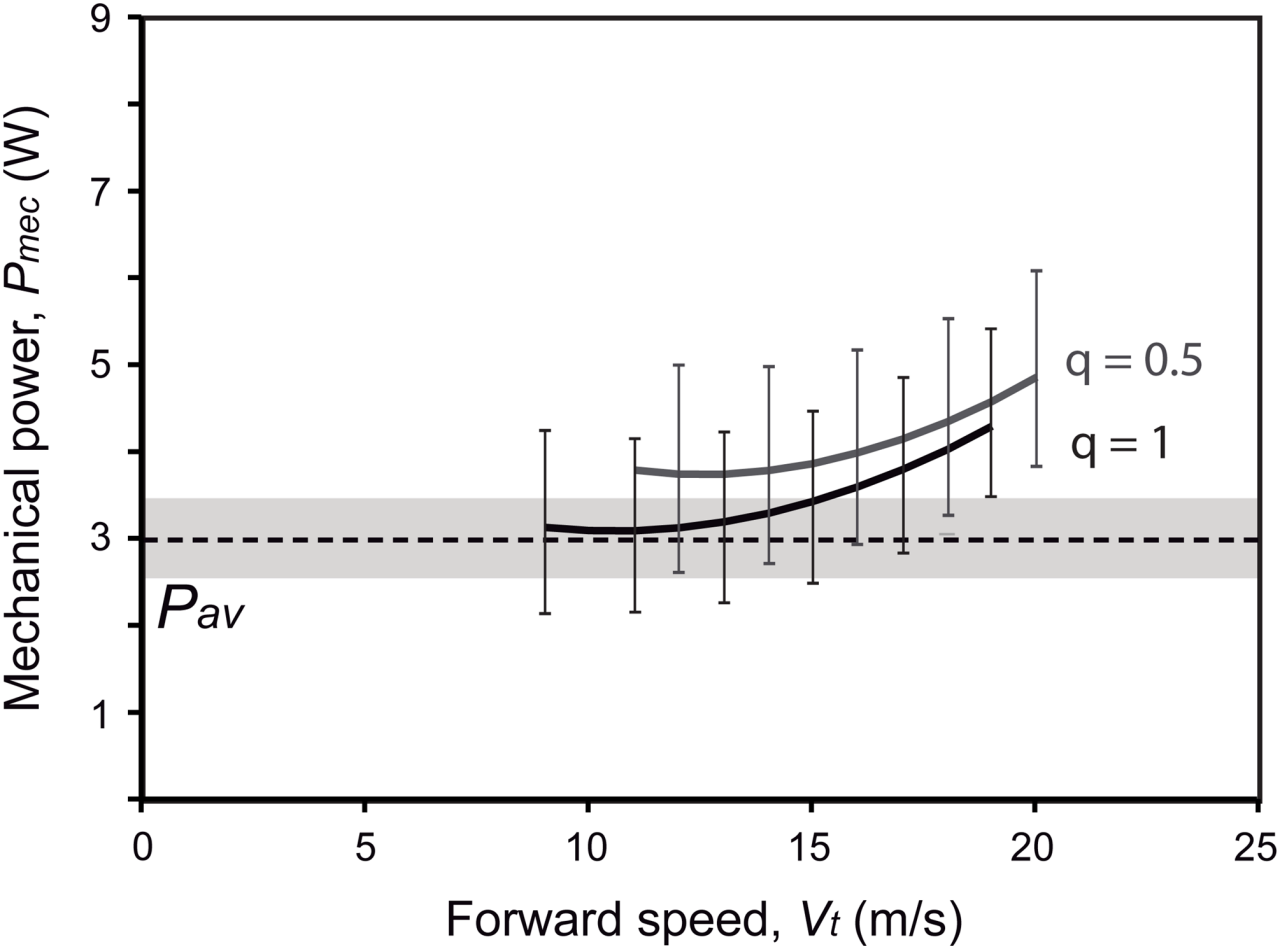
Energetic fitness for maintaining flapping flight as illustrated by the relationship between the maximum aerobic power available (*P*_av_; dashed line) and the mechanical power output (*P*_mec_; solid lines) (see Table 4). Two flying strategies are depicted: continuous flapping (black curve; *q* = 1), and bounding (gray curve; *q* = 0.5). Uncertainty ranges of the estimations (obtained from the lowest and highest estimations of body mass, wing span and wing area) are graphed as a grey region for *P*_av_ and error bars for *P*_mec_. It is worth noting that the over or underestimation of the *M*_b_ will affect both *P*_mec_ and *P*_av_ in the same way; in other words, one cannot be overestimated while the other is underestimated only due to *M*_b_ effect.

Active flapping in extant birds is constrained by the ratio between the power available from the aerobic metabolism (*P*_av_) and the mechanical power output required for flapping (*P*_mec_) (Rayner, 1988; Lindhe Norberg, 2002; Pennycuick, 2008). By assuming a negative scaling between *P*_av_ and *M_b_*, as observed in extant flying birds (Bishop & Butler, 2015), *Gretcheniao sinensis* would have generated 3.0 W. As this value is under *P*_mec_ for most speeds, either when flying through continuous flapping (i.e., *q* = 1; estimate of *P*_mec_ at *V*_mr_ is 4.0 W) or bounding (i.e., *q* = 0.5; *P*_mec_ at *V*_mr_ is 4.6 W), our analysis indicates that *P*_av_ was insufficient to cover the energetic requirements for flapping flight (Fig. 11). This negative power margin (i.e., *P*_av_ < *P*_mec_; see Table 4) rules out the possibility that *Gretcheniao sinensis* and other “bohaiornithids” could have performed long flights through either sustained flapping or bounding. Thus, unlike other early Cretaceous enantiornithines for which intermittent flight has been inferred, either bounding or flap-gliding (Serrano et al., 2017, 2018; Liu et al., 2017, 2019), *Gretcheniao sinensis* and other “bohaiornithids” probably flew through continuous flapping. However, our results suggest that they would have been unable to generate sufficient power to sustain a prolonged flight. Such a flight behavior would have been analogous to that observed in extant brief flyers such as land fowl and diving birds.

**Table 4:** Estimated parameters for flight reconstruction models of *Gretcheniao sinensis* and other “bohaiornithids.”

Species	Specimen	*C*_Db_	*P*_av_ (W)	*q* = 1		*q* = 0.5	
				*V*_mr_ (m/s)	*P*_mec_ (W)	*V*_mr_ (m/s)	*P*_mec_ (W)
*Gretcheniao sinensis*	BMNH Ph 829	0.1576	3.03	17.7	3.97	19.0	4.60
*Sulcavis geeorum*	BMNH Ph 805	0.1717	2.39	16.3	2.68	17.4	3.08
*Longusunguis kurochkini*	IVPP V 17964	0.1648	1.93	16.2	2.14	17.3	2.45
*Zhouornis hani*	CNUVB-0903	0.1585	2.92	17.5	3.79	18.8	4.40
*Zhouornis hani*	BMNH Ph 756	0.1234	2.01	18.2	2.35	19.5	2.72
*Bohaiornithidae* indet.	CUGB P1202	0.1072	1.44	18.6	1.63	19.9	1.87

**Note:**

*C*_Db_, body drag coefficient; *P*_av_, maximum available power output from aerobic metabolism; *P*_mec_, mechanical power output; *q*, duration of flapping phase with respect to ballistic phase in bounding flight (continuous flapping, *q* = 1; bounding, *q* = 0.5); *V*_mr_, maximum range speed.

## Conclusion

BMNH Ph 829 adds diversity to the overall anatomy of *Bohaiornis*-like enantiornithines (“bohaiornithids”) by providing morphological details previously unknown for these birds. The unique combination of skeletal traits of BMNH Ph 829 supports the recognition of a new species, *Gretcheniao sinensis*, which appears to represent an early divergence among these birds. Nonetheless, our extensive cladistic analyses show that despite the discovery of many well-preserved enantiornithines from the Jehol Biota (Chiappe & Meng, 2016), the interrelationships of these birds remain contentious. Our analyses do not recover a monophyletic clade of the six species that have been previously considered as “bohaiornithids”—only a subset of these are clustered in a monophyletic group. Further studies are needed to test whether these previously identified “bohaiornithids” constitute an evolutionary grade, sharing an overall morphology (largely symplesiomorphic characters) but lacking a single common ancestry. The degree to which these “bohaiornithids” may have shared a similar ecology needs to be further explored considering that some of them (e.g., *F. xiaotaizicus*) have been interpreted as highly specialized (i.e., scansorial) (Wang, O’Connor & Zhou, 2014).

Our study also provides the first assessment of the flight properties of “bohaiornithid” enantiornithines. Key flight parameters inferred for these birds suggest ineffectiveness for performing intermittent flight (either flap-gliding or bounding) that was inferred for other enantiornithines (Liu et al., 2017, 2019; Serrano et al., 2017, 2018). Instead, these parameters indicate that *Gretcheniao sinensis* and other “bohaiornithids” were morphologically suited for flying through continuous flapping, although not able to perform prolonged flights. Our aerodynamic results thus expand the previously known aerial repertoire of the Cretaceous enantiornithines.

## Appendix 1: Character list used in the cladistics analyses

Premaxillae in adults: unfused (0); fused only rostrally (1); completely fused (2). (ORDERED)
Maxillary process of premaxilla: restricted to rostral portion (0); subequal or longer than facial contribution of maxilla (1).
Frontal process of premaxilla: short (0); relatively long, approaching rostral border of antorbital fenestra (1); very long, approaching the lacrimals (2). (ORDERED)
Premaxillary teeth: present throughout (0); present but rostral tip edentulous (1); present but restricted to rostral portion (2); absent (3).
Caudal margin of naris: far anterior than rostral border of antorbital fossa (0); nearly reaching or overlapping rostral border of antorbital fossa (1).
Naris longitudinal axis: considerably shorter than long axis of antorbital fossa (0); subequal or longer (1).
Maxillary teeth: present (0); absent (1).
Dorsal (ascending) ramus of maxilla: present with two fenestra (the promaxilllary and maxillary fenestra) (0); present with one fenestra (1); unfenestrated (2); ramus absent (3). (ORDERED)
Caudal margin of choana: located rostrally, not overlapping orbital region (0); displaced caudally, at same level or overlapping rostral margin of orbit (1).
Contact between palatine and maxilla/premaxilla: palatine contact maxilla only (0); contacts premaxilla and maxilla (1).
Jugal process of palatine: present (0); absent (1).
Ectopterygoid: present (0); absent (1).
Postorbital: present (0); absent (1).
Contact between postorbital and jugal: present (0); absent (1).
Quadratojugal: sutured to quadrate (0); joined through ligamentary articulation (1).
Lateral, round cotyla on mandibular process of quadrate (quadratojugal articulation): absent (0); present (1).
Squamosal incorporated into the braincase, forming a zygomatic process: absent (0); present (1).
Squamosal, zygomatic process: variably elongate, dorsally enclosing otic process of the quadrate and extending cranioventrally along shaft of this bone, dorsal head of quadrate not visible in lateral view (0); short, head of quadrate exposed in lateral view (1).
Frontal/parietal suture in adults: open (0); close, bones fully fused to one another (1).
Quadrate orbital process (pterygoid ramus): broad (0); sharp and pointed (1).
Quadrate pneumaticity: absent (0); present (1).
Quadrate: articulating only with squamosal (0); articulating with both prootic and squamosal (1).
Otic articulation of the quadrate: articulates with a single facet (squamosal) (0); articulates with two distinct facets (prootic and squamostal) (1); articulates with two distinct facets and quadrate differentiated into two heads (2). (ORDERED)
Quadrate distal end: with two transversely aligned condyles (0); with a triangular, condylar pattern, usually composed of three distinct condyles (1).
Eustachian tubes: paired, lateral, and well-separated from each other (0); paired, close to each other and to cranial midline or forming a single cranial opening (1).
Dentary teeth: present (0); absent (1).
Robustness of teeth relative to dentary: anteroposterior width of largest tooth crowns (measured at thickest portion of crown’s base) far less than half of dentary dorsoventral depth (0); close to half (or more) of dentary depth, i.e., 45%, or more (1). Taxa near the cutoff point are coded 0/1.
Dentary tooth implantation: teeth in individual sockets (0); teeth in a communal groove (1).
Symphysial portion of dentaries: unfused (0); fused (1).
Deeply notched rostral end of mandibular symphysis: absent (0); present (1).
Small ossification present at rostral tip of mandibular symphysis (intersymphysial ossification or “predentary bone”): absent (0); present (1).
Caudal margin of dentary: unforked, or with weakly developed dorsal ramus (0); strongly forked with dorsal and ventral rami approximately equal in caudal extent (1).
Meckel’s groove of mandible (medial side of mandible): not completely covered by splenial (0); covered by splenial, not exposed medially (1).
Rostral mandibular fenestra: absent (0); present (1).
Caudal mandibular fenestra: present (0); absent (1).
Articular pneumaticity: absent (0); present (1).
Atlantal hemiarches in adults: unfused (0); fused, forming a single arch (1).
One or more pneumatic foramina piercing centra of mid-cranial cervicals, caudal to level of parapophysis-diapophysis: present (0); absent (1).
Cervical vertebrae: variably dorsoventrally compressed, amphicoelous (“biconcave”: flat to concave articular surfaces) (0); cranial articular surface heterocoelous (i.e., mediolaterally concave, dorsoventrally convex), caudal articular surface flat or slightly concave (1); heterocoelous cranial (i.e., mediolaterally concave, dorsoventrally convex) and caudal (i.e., mediolaterally convex, dorsoventrally concave) articular surfaces (2). (ORDERED)
Prominent carotid processes in intermediate cervicals: absent (0); present (1).
Postaxial cervical epipophyses: prominent, projecting further back from postzygapophyses (0); weak, not projecting further back from postzygapophyses, or absent (1).
Keel-like ventral surface of cervical centra: absent (0); present (1).
Prominent (50% or more the height of centrum’s cranial articular surface) ventral processes of cervicothoracic vertebrae: absent (0); present (1).
Thoracic vertebral count: 13–14 (0); 11–12 (1); fewer than 11 (2). The transition between cervical and thoracic vertebrae is often difficult to identify, which makes counting these vertebrae problematic; we identify the first vertebra in articulation with a long costal rib as the first thoracic vertebra (ORDERED)
Thoracic vertebrae: at least part of series with subround, central articular surfaces (e.g., amphicoelous/opisthocoelous) that lack the dorsoventral compression seen in heterocoelous vertebrae (0); series completely heterocoelous (1).
Thoracic vertebrae, lateral side of centra: weakly or not excavated (0); deeply excavated by a groove (1); excavated by a broad fossa (2).
Cranial thoracic vertebrae, parapophyses: located in cranial part (0) or central part (1) of centra.
Sacral vertebrae, number ankylosed centra (synsacrum): less than 7 (0); 7 (1); 8 (2); 9 (3); 10 (4); more than 10 (5). (ORDERED)
Synsacrum, procoelous articulation with last thoracic centrum (deeply concave facet of synsacrum receives convex articulation of last thoracic centrum): absent (0); present (1).
Cranial vertebral articulation of first sacral vertebra: approximately equal in height and width (0); wider than high (1).
Degree of fusion of distal caudal vertebrae: fusion absent (0); few vertebrae partially ankylosed (intervening elements are well-discernable) (1); vertebrae completely fused into a pygostyle (2). (ORDERED)
Distal caudal vertebra prezygapophyses: elongate, exceeding the length of centrum by more than 25% (0); shorter (1); absent (2). (ORDERED)
Pygostyle: longer than or equal to the combined length of free caudals (0); shorter (1).
Cranial end of pygostyle dorsally forked: absent (0); present (1).
Cranial end of pygostyle with a pair of laminar, ventrally projected processes: absent (0); present (1).
Distal constriction of pygostyle: absent (0); present (1).
Ossified uncinate processes in adults: absent (0); present and free (1); present and fused (2).
Gastralia: present (0); absent (1).
Coracoid shape: rectangular to trapezoidal in profile (0); strutlike (1).
Coracoid-scapula articulation: “ball and socket” articulation (i.e., pit-shaped scapular cotyla developed on coracoid, and coracoidal tubercle developed on scapula) (0); scapular articular surface of coracoid convex (1); flat (2).
Scapula: articulated at omal end of coracoid (0); well below it (1).
Coracoid, humeral articular facet (glenoid): dorsal to acrocoracoid process (“biceps tubercle”) (0); ventral to acrocoracoid process (1).
Humeral articular facets of coracoid and scapula: placed in same plane (0); forming a sharp angle (1).
Coracoid, acrocoracoid: straight (0); hooked medially (1).
Coracoid, laterally compressed omal end with nearly aligned acrocoracoid process, humeral articular surface, and scapular facet, in dorsal view: absent (0); present (1).
Coracoid, procoracoid process: absent (0); present (1).
Coracoid, broad, deep fossa on dorsal surface (dorsal coracoidal fossa): absent (0); present (1).
Coracoid, supracoracoidal nerve foramen: centrally located (0); displaced toward (often as an incisure) medial margin (1); absent (i.e., nerve position is displaced so that it no longer passes through coracoid) (2). (ORDERED).
Coracoid, medial surface, strongly depressed elongate furrow (usually levelled with passage of n. supracoracoideus): absent (0); present (1).
Coracoid, width of the sternal end relative to the length along the shaft: approximately half or greater (0); between half to 1/3 (1); less than 1/3 (2).
Coracoid, sternal margin: convex (0); nearly straight (1); concave (2)
Coracoid, supracoracoid nerve foramen, location relative to dorsal coracoidal fossa: above fossa (0); inside fossa (1).
Coracoid, sternolateral corner: unexpanded (0); expanded (1); well developed squared-off lateral process (sternocoracoidal process) (2); present and with distinct omal projection (hooked) (3).
(244) Scapula and coracoid: fused (0); unfused (1).
Scapula, blade: straight (0); sagittally curved (1).
Scapula, length: shorter than humerus (0); as long as or longer than humerus (1).
(245) Scapula, acromion process length relative to length of humeral articular facet: less than half (0); nearly equivalent (1); longer but less than two times (2); more than two times longer (3). (ORDERED)
Scapula, acromion process: in lateral or costal view, strongly projecting craniodorsally, forming a large angle with proximal shaft (0); nearly parallel to shaft (1).
Scapula, costal surface of blade with prominent longitudinal furrow: absent (0); present (1).
Scapula, caudal end: blunt (may or may not be expanded) (0); sharply tapered (1).
Furcula: boomerang-shaped (0); V to Y-shaped (1); U-shaped (2).
Furcula, interclavicular angle: approximately 90° (0); less than 70° (1). The interclavicular angle is measured as the angle formed between three points: the omal ends of the rami and the center of the clavicular symphysis.
Furcula, dorsal and ventral margins: subequal in width (0); ventral margin distinctly wider than dorsal margin so that furcular ramus appears concave laterally (1).
Furcula, hypocleideum: absent (0); present as a tubercle or short process (1); present as elongate process approximately 30–50% the length of rami (2); hypertrophied, exceeding 50% the length of rami (3). (ORDERED)
Sternum: unossified (0); partially ossified, coracoidal facets cartilaginous (1); fully ossified (2).
Sternum, ossification: two flat bony plates (0); single, more or less flat element (1); single element, with slightly raised midline ridge (2); single element, with strongly projected carina (3).
Sternum, carina: near to, or projecting rostrally from, cranial border (0); not reaching cranial border (1).
Sternum, caudal margin, number of paired caudal trabeculae: none (0); one (1); two (2).
Sternum, outermost trabeculae: tips terminate cranial to caudal end of sternum (0); tips terminate at or approaching caudal end of sternum (1); tips extend caudally past the end of sternal midline (2).
Sternum, distal expansion of outermost trabecula: absent (0); present, simple bulb-like (1); fan-shaped (2); triangular–shaped with acute medial angle (3); branched (4).
Sternum, rostral margin broad and rounded: absent (0); present (1).
Sternum, coracoidal sulci spacing on cranial edge: widely separated mediolaterally (0); adjacent (1); crossed on midline (2). In taxa such as *Eoalulavis* in which the preserved sternum does not bear actual sulci, the placement of the coracoids (seemingly in their original place) can be used to infer their position relative to the sternum.
Sternum, costal facets: absent (0); present (1).
Sternum, caudal half, paired enclosed fenestra: absent (0); present (1).
Sternum, dorsal surface, pneumatic foramen (or foramina): absent (0); present (1).
(243) Sternum, outermost trabecula: mainly parallel to long axis of sternum (0); clearly directed caudo-laterally (1).
Humerus, proximal and distal ends: twisted (0); nearly co-planar (1)
Humerus, head: concave cranially and convex caudally (0); globe shaped, craniocaudally convex (1).
Humerus, proximal margin of head is concave in its central portion, rising ventrally and dorsally: absent (0); present (1).
Humerus, proximocranial surface, well-developed circular fossa on midline: absent (0); present (1).
Humerus, transverse ligamental groove: absent (0); present (1).
Humerus, ventral tubercle projected caudally, separated from humeral head by deep capital incision: absent (0); present (1).
Humerus, pneumatic fossa in caudoventral corner of proximal end: absent or rudimentary (0); well developed (1).
Humerus, deltopectoral crest: projected dorsally (the plane of the crest is coplanar to cranial surface of humerus) (0); projected cranially (1).
Humerus, deltopectoral crest width: less than shaft width (0); approximately same width (1); prominent and subquadrangular (i.e., subequal length and width) (2).
Humerus, deltopectoral crest, distal end recedes abruptly with the humeral shaft: present (0); absent (1)
Humerus, deltopectoral crest: imperforated (0); perforated by a fenestra (1).
Humerus, bicipital crest: little to no cranial projection (0); developed as cranial projection relative to shaft surface in ventral view (1); hypertrophied, rounded tumescence (2).
Humerus, distal end of bicipital crest, pit-shaped fossa for muscular attachment: absent (0); craniodistal on bicipital crest (1); directly ventrodistal at tip of bicipital crest (2); caudodistal, variably developed as a fossa (3).
Humerus, demarcation of muscle origins (e.g., m. extensor metacarpi radialis) on the dorsal edge of the distal humerus: no indication (0); a pit or a tubercle (1); a variably projected scar-bearing tubercle (dorsal supracondylar process) (2).
Humerus, well-developed brachial depression on cranial face of distal end: absent (0); present (1).
Humerus, well-developed olecranon fossa on caudal face of distal end: absent (0); present (1).
Humerus, groove for passage of m. scapulotriceps: absent (0); present (1).
Humerus, m. humerotricipitalis groove: absent (0); present as a well-developed ventral depression contiguous with the olecranon fossa (1).
Humerus, distal margin: approximately perpendicular to long axis of shaft (0); strongly angled ventrally (ventrodistal margin projected significantly distal to dorsodistal margin) (i.e., well-projected flexor process (1).
Humeral distal condyles: mainly located on distal aspect (0); on cranial aspect (1).
Humerus, long axis of dorsal condyle: at low angle to humeral axis, proximodistally oriented (0); at high angle to humeral axis, almost transversely oriented (1).
Humerus, distal condyles: subround, bulbous (0); weakly defined, “straplike” (1).
Humerus, ventral condyle: length of long axis less than the same measure in dorsal condyle (0); same or greater (1).
Ulna: shorter than humerus (0); nearly equivalent to or longer than humerus (1).
Ulna: mid-shaft relative width: radial-shaft/ulnar-shaft ratio larger than 0.70 (0); smaller than 0.70 (1).
Ulna, cotylae: dorsoventrally adjacent (0); widely separated by deep groove (1).
Ulna, dorsal cotyla strongly convex: absent (0); present (1).
Ulna, bicipital scar: absent (0); developed as slightly raised scar (1); developed as conspicuous tubercle (2).
Ulna, proximal end with well-defined area for insertion of m. brachialis anticus: absent (0); present (1).
Ulna, semilunate ridge on dorsal condyle: absent (0); present (1).
Radius, long longitudinal groove on ventrocaudal surface of shaft: absent (0); present (1).
Ulnare: heart-shaped with little differentiation into short rami (0); U-shaped to V-shaped, well-developed rami (1).
Semilunate carpal and proximal ends of metacarpals in adults: unfused (0); semilunate fused to the alular (I) metacarpal (1); semilunate fused to the major (II) and minor (III) metacarpals (2); fusion of semilunate and all metacarpals (3). Juvenile specimens are scored as “?” to account for the possibility of ontogenetic change.
Semilunate carpal, position relative to alular metacarpal (I): over entire proximal surface (0); over less than one-half proximal surface or no contact present (1).
Carpometacarpus, proximal ventral surface: flat (0); raised ventral projection contiguous with minor metacarpal (1); pisiform process forming a distinct peg-like projection (2).
Carpometacarpus, proximoventral surface, supratrochlear fossa deeply excavating proximal surface of pisiform process: absent (0); present (1).
Alular metacarpal (I), round-shaped: absent (0); present (1).
Alular metacarpal (I), extensor process: absent (0); tip barely (1) or conspicuously (2) surpasses cranial margin of distal articular facet. (ORDERED)
Alular metacarpal (I), distal articulation with proximal phalanx: ginglymoid (0); shelf (1); ball-like (2).
Minor metacarpal (III), craniocaudal diameter as percentage of same dimension of major metacarpal (II): approximately equal or greater than 50% (0); less than 50% (1).
Alular digit (I), proximal phalanx: longer than proximal phalanx of major digit (II) (0); shorter than or equivalent to proximal phalanx of major digit (II) (1).
Intermetacarpal process (or tubercle) on major metacarpal (II): absent (0); present (1).
Intermetacarpal space: absent or very narrow (0); at least as wide as maximum width of minor metacarpal (III) shaft (1).
Intermetacarpal space: reaches proximally as far as distal end of alular metacarpal (I) (0); terminates distal to end of alular metacarpal (I) (1).
Distal end of metacarpals: unfused (0); partially or completely fused (1).
Minor metacarpal (III) projecting distally more than major metacarpal (II): absent (0); present (1).
Alular digit (I), proximal phalanx, distal extension relative to major metacarpal (II): beyond distal end of major metacarpal (II) (0); approximately equal in distal extension (1); shorter than distal end but beyond half of major metacarpal (II) (2); terminating less than half of major metacarpal (II) (3). (ORDERED)
Proximal phalanx of major digit (II): round-shaped cross section (0); flat and craniocaudally expanded (1).
Intermediate phalanx of major digit (II): longer than proximal phalanx (0); shorter than or equivalent to proximal phalanx (1).
Ungual phalanx of major digit (II): present (0); absent (1).
Ungual phalanx of major digit (II): larger or subequal to other manual unguals (0); smaller than alular ungual but larger than ungula phalanx of minor (III) digit (ungual of minor digit may or may not be present) (1); smaller than unguals of alular and minor digits (2).
Ungual phalanx of minor digit (III): present (0); absent (1).
Manus, relative length: length of semilunate carpal + major metacarpal and digit longer than humerus (0); subequal (1); shorter (2). (ORDERED)
Intermembral index = (length of humerus + ulna)/(length of femur + tibiotarsus): less than 0.7 (0); between 0.7 and 0.9 (1); between 0.9 and 1.1 (2); greater than 1.1 (3).
Pelvis, bone fusion at level of acetabulum: unfused or partial fusion (0); completely fused (1). Juvenile specimens with unfused pelvis are scored “?” to allow for ontogenetic fusion.
Ilium/ischium, distal co-ossification to completely enclose ilioischiadic fenestra: absent (0); present (1).
Ilium, midline proximity of preacetabular wings: separated (may exist cartilaginous connection) (0); co-ossified, dorsal closure of “iliosynsacral canals” (1).
Preacetabular pectineal process (preacetabular tubercle of Baumel): absent (0); present (1).
Pelvis, acetabulum proportions: large acetabulum, acetabulum/ilium length ratio greater than 0.11 (0); small acetabulum, same proportion equal or smaller than 0.11 (1).
Prominent antitrochanter: caudally directed (0); caudodorsally directed (1).
Ilium, postacetabular process: deep (0), more than 50% of depth of preacetabular wing at level of acetabulum; shallow (1), less than 50%.
Ilium, brevis fossa: present (0); absent (1).
Ischium, relative length: two-thirds or less the length of pubis (0); more than two-thirds the length of pubis (1).
Ischium, obturator process: prominent (0); reduced or absent (1).
Ischium, proximodorsal (or proximocaudal) process: absent (0); present (1).
Pubis, orientation of proximal portion: cranially to subvertically oriented (0); retroverted, separated from main synsacral axis by a 45–65° angle (1); more or less parallel to ilium and ischium (2). (ORDERED)
Ilium, pubic pedicel very compressed laterally and hook-like: absent (0), present (1).
Pubis, shaft laterally compressed throughout its length: absent (0); present (1).
Pubis, pubic apron: present (0); absent (absence of symphysis) (1).
Pubis, distal foot: flaring into simple round shape (0); triangular shape with pointed caudal tip and caudoventally directed with respect to distal pubic shaft (1); caudal tip recurved caudodorsally with respect to distal pubic shaft (2); absent (3).
Femur, distinct fossa for capital ligament: absent (0); present (1).
Femur, neck: present (0); absent (1).
Femur, anterior trochanter: separated from greater trochanter (0); fused to it, forming a trochanteric crest with laterally curved edge (1); fused to it, forming a trochanteric crest with flattened edge (2).
Femur, trochanteric crest: projects proximally beyond femoral head (0); equal in proximal projection (1); does not project beyond femoral head (2).
Femur, posterior trochanter: present, developed as slightly projected tubercle or flange (0); hypertrophied, “shelf-like” conformation (1); absent (2).
Femur, prominent patellar groove: absent (0); present as continuous extension onto distal shaft (1); present and separated from shaft by slight ridge, giving it a pocketed appearance (2).
Femur, lateral distal end: ectocondylar tubercle and lateral condyle separated by deep notch (0); ectocondylar tubercle and lateral condyle contiguous but without developing a tibiofibular crest (1); tibiofibular crest present, defining laterally a fibular trochlea (2). (ORDERED)
Femur, popliteal fossa distally bounded by a complete transverse ridge: absent (0); present (1).
Tibia, calcaneum, and astragalus: unfused or poorly co-ossified (sutures still visible) (0); complete fusion of tibia, calcaneum, and astragalus (1).
Tibia, round proximal articular surface: absent (0); present (1).
Tibia, proximal articular surface: flat (0); angled so that medial margin is elevated with respect to lateral margin (1).
Tibiotarsus, proportions: tibiotarsus length/tarsometatarsus length equals 2 or more (0); between 2 and 1.6 (1); smaller than 1.6 (2). When distal tarsals are not fused with metatarsals, metatarsal III length is used.
Tibiotarsus, cnemial crests: absent (0); present, one (1); present, two (2).
Tibia, caudal extension of articular surface for tarsals/tarsometatarsus: absent, articular surface restricted to distalmost edge of caudal surface (0); well-developed caudal extension, sulcus cartilaginis tibialis (Baumel & Witmer, 1993), distinct surface extending up caudal surface of tibiotarsus (1); with well-developed, caudally projecting medial and lateral crests (2). (ORDERED)
Tibiotarsus, extensor canal: absent (0); present as emarginate groove (1); groove bridged by ossified supratendinal bridge (2). (ORDERED)
Tibibiotarsus, condyles, cranial projection: medial condyle projecting farther cranially than lateral condyle (0); equal in cranial projection (1).
Tibiotarsus, condyles, relative mediolateral width: medial condyle wider (0); approximately equal (1); lateral condyle wider (2). (ORDERED).
Tibiotarsus, condyles, intercondylar groove: mediolaterally broad, approximately 1/3 with of anterior surface (0); less than 1/3 width of anterior surface (1).
Tibiotarsus, condyles: gradual sloping of condyles towards tibiotarsal midline of tibiotarsus (0); no tapering of either condyle (1).
Fibula, proximal end: prominently excavated by medial fossa (0); nearly flat (1).
Fibula, tubercle for m. iliofibularis: craniolaterally directed (0); laterally directed (1); caudolaterally or caudally directed (2). (ORDERED)
Fibula, distal end reaching proximal tarsals: present (0); absent (1).
Distal tarsals in adults: free (0); completely fused to the metatarsals (1). Juvenile specimens are scored as “?” in order to account for the possibility of ontogenetic change.
Metatarsals II–IV, intermetatarsal fusion: absent or minimal co-ossification (0); partial fusion, sutural contacts easily discernible (1); completely or nearly completely fused, sutural contacts absent or poorly demarcated (2). (ORDERED)
Metatarsal V: present (0); absent (1).
Metatarsal III, proximal end: co-planar with metatarsals II and IV (0); plantarly displaced with respect to metatarsals II and IV (1).
Tarsometatarsus, proximal vascular foramen and/or foramina between metatarsals III and IV: absent (0); one (1); two (2).
Tarsometatarsus, intercotylar eminence: absent (0); present, low and rounded (1); present, high and peaked (2).
Tarsometatarsus, projected surface and/or grooves on proximocaudal surface (associated with the passage of tendons of pes flexors; hypotarsus): absent (0); developed as caudal projection with flat caudal surface (1); at least one groove enclosed by bone caudally (2). (ORDERED)
Tarsometatarsus, plantar surface: flat (0); excavated (1).
Tarsometatarsus, distal vascular foramen completely enclosed by metatarsals III and IV: absent (0); present (1).
Metatarsal I: straight (0); J-shaped, with short projection (articulation of hallux) extending medially (1); J-shaped; articulation of hallux extending caudally (2); distal half of metatarsal I is laterally deflected so that laterodistal surface is concave (3).
Metatarsal II tubercle (associated with the insertion of the tendon of the *m. tibialis cranialis*): absent (0); present, approximately centered on proximodorsal surface of metatarsal II (1); present, developed on lateral surface of metatarsal II, at contact with metatarsal III or on lateral edge of metatarsal III (2).
Metatarsal II, distal plantar surface, fossa for metatarsal I: absent (0); shallow notch (1); conspicuous ovoid fossa (2). (ORDERED)
Relative position of metatarsal trochleae: trochlea III more distal than trochleae II and IV (0); trochlea III at same level as trochlea IV, both more distal than trochlea II (1); trochlea III at same level as trochleae II and IV (2); distal extent of trochlea III intermediate to trochlea IV and II where trochlea IV projects furthest distally (3).
Metatarsal II, distal extent of metatarsal II relative to metatarsal IV: approximately equal in distal extent (0); metatarsal II shorter than metatarsal IV but reaching distally farther than base of metatarsal IV trochlea (1); metatarsal II shorter than metatarsal IV, reaching distally only as far as base of metatarsal IV trochlea (2).
Tarsometatarsus, trochlea in distal view: aligned in a single plane (0); metatarsal II slightly displaced plantarly with respect to III and IV (1); metatarsal II strongly displaced plantarly in respect to III and IV, such that there is little or no overlap in medial view (2).
Metatarsal II, trochlea of metatarsal II broader than trochlea of metatarsal III: absent (0); present (1).
Metatarsal III, trochlea in plantar view, proximal extent of lateral and medial edges of trochlea: trochlear edges approximately equal in proximal extent (0); medial edge extends farther (1).
Distal end of metatarsal II strongly curved medially: absent (0); present (1).
Phalanx in digit IV; second and third phalanges reduced and significantly shorter than fourth phalanx: absent (0), present (1), present, but with proximal phalanx reduced to be nearly equal in length with second and third phalanx (2).
Digit IV phalanges in distal view, medial trochlear rim enlarged with respect to lateral trochlear rim: absent (0); present, lateral trochlea reduced to a rounded peg (1).
Pes, proximal phalanx of hallux is the longest non-ungual phalanx: absent (0); present (1).
Size of claw of hallux relative to other pedal claws: shorter, weaker, and smaller (0); similar in size (1); longer, more robust, and larger (2).
Alula: absent (0); present (1).
Fan-shaped feathered tail composed of more than two elongate retrices: absent (0); present (1).

## Appendix 2: Character matrix

**Table:** 

Taxon\character	1	2	3	4	5	6	7	8	9	10	11	12	13	14	15	16	17	18	19	20	21	22	23	24	25	26	27	28	29	30
*Archaeopteryx*	0	0	0	0	0	0	0	0	0	0	0	0	0	0	0	0	0	?	0	0	?	0	0	0	0	0	0	0	0	0
*Jeholornis*	0/1	0	2	3	1	1	0	?	?	?	?	?	?	?	?	0	?	?	?	?	?	?	?	?	?	0	0	0	?	?
*Sapeornis*	1	0	1	0	0	1	0	0/1	?	?	?	?	0	0	1	0	1	?	0	?	?	?	?	?	?	1	?	?	0	?
*Confuciusornis*	1	0	2	3	1	1	1	1	?	?	?	0	0	0	1	0	1	0	0	?	0	1	2	0	?	1	?	?	0	1
*Changchengornis*	1	?	2	3	1	?	1	1	?	?	?	?	?	?	1	?	?	?	?	?	0	?	?	?	?	1	?	?	1	1
*Eoconfuciusornis*	1	0	2	3	1	?	1	0/1	?	?	?	?	0	0	?	?	?	?	?	?	?	?	?	?	?	1	?	?	0	1
*Protopteryx*	0/1	0	1	0	1	1	0	?	?	?	?	?	?	?	?	?	?	?	?	?	?	?	?	?	?	0	0	?	0	?
*Orienantius*	0/1	0	1/2	0	?	?	0	?	?	?	?	?	?	?	?	?	?	?	0	?	?	?	?	?	?	0	0	0	0	0
*Eopengornis*	0	0	0	0	*1*	1	0	0/1/2	?	?	?	?	?	?	?	?	?	?	0	?	?	?	?	?	?	0	0	0	?	0
*Parapengornis*	0	0	0	0	1	?	0	0/1/2	?	?	?	?	?	?	1	?	?	?	0	?	?	?	0/1	0	?	0	0	0	0	?
*Junornis*	1/2	0	1/2	0/2	1	?	0	0/1/2	?	?	?	?	?	?	?	?	?	?	0	?	?	?	?	?	?	0	0/1	0	0	?
*Concornis*	?	?	?	?	?	?	?	?	?	?	?	?	?	?	?	?	?	?	?	?	?	?	?	?	?	?	?	?	?	?
*Elsornis*	?	?	?	?	?	?	?	?	?	?	?	?	?	?	?	?	?	?	?	?	?	?	?	?	?	?	?	?	?	?
*Eoalulavis*	?	?	?	?	?	?	?	?	?	?	?	?	?	?	?	?	?	?	?	?	?	?	?	?	?	?	?	?	?	?
*Cathayornis*	1/2	0	1/2	0	?	?	0	?	?	?	?	?	?	?	?	?	?	?	0	?	?	?	?	?	?	0	0	0	0	?
*Eocathayornis*	?	0	?	0	?	?	?	?	?	?	?	?	?	?	?	?	?	?	0	?	?	?	0	?	?	0	?	0	0	?
*Eoenantiornis*	0/1	0	1	0/1	?	?	0	0/1	?	?	?	?	?	?	?	?	?	?	0	?	?	?	?	?	?	0	0	0	?	?
*Gobipteryx*	1	0	1/2	3	1	0	1	2	0	0	1	0	?	?	?	?	?	?	?	0	?	?	?	0	?	1	?	?	0	0
*Iberomesornis*	?	?	?	?	?	?	?	?	?	?	?	?	?	?	?	?	?	?	?	?	?	?	?	?	?	?	?	?	?	?
*Longipteryx*	?	0	2	2	1	1	1	?	?	?	?	?	?	?	?	?	?	?	?	?	?	?	?	?	?	0	1	0	0	?
*Longirostravis*	0/1	0	2	2	?	?	1	?	?	?	?	?	?	?	?	?	?	?	?	?	?	?	?	?	?	0	0	0	?	?
*Neuquenornis*	?	?	?	?	?	?	?	?	?	?	?	?	?	?	?	?	?	?	0	?	?	?	?	?	?	?	?	?	?	?
*Pengornis*	0	0	0/1	0	1	1	0	1	?	?	?	?	0	?	?	?	?	?	0	?	1	?	?	?	?	0	0	0	0	?
*Rapaxavis*	0/1	0	2	2	?	?	1	0/1/2	?	?	?	?	?	?	?	?	?	?	?	?	?	?	0/1	?	?	0	0	0	0	?
*Shanweiniao*	0/1	?	1/2	1/2	?	?	1	?	?	?	?	?	?	?	?	?	?	?	?	?	?	?	?	?	?	?	0	?	0	?
*Vescornis*	?	?	?	?	?	?	?	0/1/2	?	?	?	?	?	?	?	?	?	?	?	?	?	?	?	?	?	0	0	0	0	0
*Vorona*	?	?	?	?	?	?	?	?	?	?	?	?	?	?	?	?	?	?	?	?	?	?	?	?	?	?	?	?	?	?
*Schizooura*	0	1	2	3	1	1	1	0/1	?	?	?	?	?	?	?	?	?	?	?	?	?	?	?	?	?	1	?	?	0	?
*Jianchangornis*	?	?	?	?	?	?	?	?	?	?	?	?	?	?	?	?	?	?	0	?	?	?	?	?	?	0	0	?	0	0
*Archaeorhynchus*	1	0	1	3	1	1	1	1/2	?	?	?	?	?	?	?	?	?	?	?	?	0	1	2	?	?	1	?	?	0	0
*Longicrusavis*	1	0	1/2	0	1	?	0	?	?	?	?	?	?	?	?	?	?	?	?	?	?	?	?	?	?	?	?	?	0	0
*Apsaravis*	?	?	?	?	?	?	?	?	?	?	?	?	?	?	?	?	?	?	?	?	?	1	0/1	?	?	1	?	?	1	1
*Hongshanornis*	?	0	2	?	1	1	0	?	?	?	?	?	?	1	?	?	?	?	?	?	?	?	?	?	?	0	0	0	0	?
*Yanornis*	1	0	2	1	1	1	0	2	?	?	?	?	?	?	1	?	?	?	?	?	?	?	?	?	?	0	0	0	0	?
*Patagopteryx*	?	?	?	?	?	?	?	?	?	?	?	?	1	?	1	1	1	0	0	0	1	1	1	1	?	?	?	?	?	?
*Yixianornis*	1	0	2	1	?	?	?	?	?	?	?	?	?	?	?	?	?	?	0	?	?	?	1	?	?	0	0	?	0	?
*Gansus yumenensis*	?	?	?	?	?	?	?	?	?	?	?	?	?	?	?	?	?	?	?	?	?	?	?	?	?	?	?	?	?	?
*Gansus zheni*	?	?	?	?	1	0	?	3	?	?	?	?	?	?	?	?	?	?	?	1	?	?	?	?	?	0	0	?	0	?
*Ichthyornis*	1/2	1	2	?	?	?	0	?	?	?	?	?	?	?	1	1	?	?	?	?	1	1	1	0	0	0	0	0	0	?
*Hesperornis*	1	1	2	3	1	1	0	3	?	?	1	1	1	?	1	1	1	1	0	1	1	1	1	1	?	0	0	1	0	0
*Parahesperornis*	1	1	2	3	1	1	0	?	?	?	1	1	1	?	1	1	1	1	0	?	1	1	1	1	?	0	0	1	0	0
*Vegavis*	?	?	?	?	?	?	?	?	?	?	?	?	?	?	?	?	?	?	?	?	?	?	?	?	?	?	?	?	?	?
*Anas*	2	1	2	3	0	1	1	3	1	1	1	1	1	?	1	1	1	1	1	1	1	1	2	1	1	1	?	?	1	0
*Gallus*	1	1	2	3	1	1	1	3	1	1	1	1	1	?	1	1	1	1	1	1	1	1	2	1	1	1	?	?	1	0
*Bellulornis*	?	?	?	?	?	?	?	?	?	?	?	?	?	?	?	?	?	?	?	?	?	?	?	?	?	?	?	?	?	?
*Fortunguavis*	?	?	?	?	?	?	0	?	?	?	?	?	?	?	?	?	?	?	?	?	?	?	?	?	?	?	?	?	?	?
*Qiliania*	?	?	?	?	?	?	?	?	?	?	?	?	?	?	?	?	?	?	?	?	?	?	?	?	?	?	?	?	?	?
*shenqiornis*	0/1	0	1	0	1	1	0	2	?	?	?	?	0	?	?	?	?	?	0	?	1	?	?	?	?	0	1	0	0	?
*Sulcavis*	0/1	0	1	0	?	?	0	?	?	?	?	?	0	?	?	?	?	?	0	?	?	?	?	?	?	0	1	0	0	?
*Bohaiornis*	1	0	1	0	1	1	0	2	?	?	?	?	?	?	?	?	?	?	0	?	?	?	?	?	?	0	1	0	0	?
*Parabohaiornis*	?	0	2	0	1	0	0	2	?	?	?	?	?	?	?	?	?	?	0	?	?	?	?	?	?	0	1	0	0	?
*Longusunguis*	0/1	0	1/2	0	1	?	0	2	?	?	?	?	?	?	?	?	?	?	?	?	?	?	?	?	?	0	1	0	0	?
*Zhouornis*	0/1	0	1/2	0	1	?	0	1	?	?	?	?	?	?	?	?	?	?	0	?	1	?	?	?	?	0	0/1	0	0	?
CUGB P1202	?	?	1/2	0	1	?	0	2	?	?	?	?	?	?	?	?	?	?	?	?	?	?	?	?	?	0	0/1	?	0	?
*Gretcheniao*	?	?	?	0/2	?	?	0	?	?	?	?	?	?	?	?	?	?	?	0	?	?	?	?	?	?	0	0/1	?	0	?

Taxon\character	31	32	33	34	35	36	37	38	39	40	41	42	43	44	45	46	47	48	49	50	51	52	53	54	55	56	57	58	59	60
*Archaeopteryx*	0	0	0	0	0	?	0	0	0/1	?	1	0	0	0	0	0	0	0	0	?	0	1	?	?	?	?	0	0	0	?
*Jeholornis*	?	0	?	0	0	?	?	?	0	?	?	?	?	0	0	0	0	0	?	?	0	0	?	?	?	?	?	0	1	0
*Sapeornis*	?	0	?	0	1	?	?	?	2	?	1	0	0	0/1	0	1	0	1	?	?	2	0	1	0	?	0	0	0	0	?
*Confuciusornis*	0	1	1	1	0	0	1	0	1	?	1	0	1	0/1	0	2	0	1	1	0	2	0	0	0	0	0	1	0	1	?
*Changchengornis*	0	?	?	1	0	0	?	?	1/2	?	?	?	?	?	?	2	?	1	?	?	2	?	0	0	0	?	?	0	1	?
*Eoconfuciusornis*	0	0	?	1	0	0	?	?	?	0	?	0	?	0/1	0	0	?	1	?	?	2	?	0	0	0	0	?	0	?	?
*Protopteryx*	0	0	?	?	?	?	?	?	?	?	1	?	?	0/1	0	2	?	?	?	?	2	?	?	?	?	0	1	0	1	?
*Orienantius*	0	0	?	?	?	?	?	?	0	?	1	?	?	1	0	1	1	1	?	?	2	0	1	?	?	0	?	0	1	?
*Eopengornis*	0	0	?	?	?	?	?	?	1	?	?	?	?	?	0	?	?	?	?	?	2	?	1	?	?	?	?	0	1	?
*Parapengornis*	0	?	?	0	?	?	?	?	1	?	*1*	?	?	?	0	1	1	?	?	?	2	1	*1*	0	?	*0*	1	0	1	?
*Junornis*	?	0	?	?	?	?	?	?	?	?	?	?	?	?	0	?	?	1	?	?	2	?	1	1	?	0	?	?	1	?
*Concornis*	?	?	?	?	?	?	?	?	?	?	?	?	?	?	0	1	1	?	?	?	?	?	?	?	?	?	0	?	1	1
*Elsornis*	?	?	?	?	?	?	?	?	0/1	?	?	1	?	?	0	1	0	?	?	?	?	?	?	?	?	?	?	?	1	1
*Eoalulavis*	?	?	?	?	?	?	?	?	0	0	1	1	1	2	0	2	?	?	?	?	?	?	?	?	?	?	0	?	1	1
*Cathayornis*	?	0	?	?	?	?	?	?	?	?	?	1	?	?	0	1	1	?	?	?	2	?	0	1	1	1	?	?	1	1
*Eocathayornis*	?	0	?	?	?	?	?	?	?	?	?	1	?	?	0	0	1	?	?	?	2	?	?	?	?	?	?	?	1	1
*Eoenantiornis*	?	?	?	?	?	?	?	?	?	?	?	?	?	?	?	?	?	?	?	?	2	?	?	1	?	?	1	0	1	1
*Gobipteryx*	0	1	?	?	1	0	?	?	?	?	?	1	?	?	?	0	?	?	0	0	1/2	1	?	?	?	?	?	?	1	1/2
*Iberomesornis*	?	?	?	?	?	?	?	?	0	?	?	1	1	1	0	0	0	0	0	?	2	1	0	?	?	?	?	?	1	?
*Longipteryx*	?	0	?	0	1	?	?	?	1	1	1	1	?	?	?	?	?	2/3/4	0	?	2	?	0	1	?	1	1	0	1	?
*Longirostravis*	?	0	?	0	1	?	?	?	1	?	?	?	?	?	0	?	?	1	?	?	2	?	0	1	1	1	0	0	1	1
*Neuquenornis*	?	?	?	?	?	?	?	?	?	?	?	?	?	?	0	1	1	?	?	?	?	?	?	?	?	?	?	?	1	?
*Pengornis*	0	?	?	?	?	?	1	?	1/2	?	?	1	?	?	0	1	0	1	?	?	2	?	?	?	?	?	?	0	1	?
*Rapaxavis*	0	?	?	0	1	?	?	?	?	?	?	1	?	?	0	1	1	0/1	?	?	2	?	0	?	1	1	0	0	1	?
*Shanweiniao*	?	0	?	0	1	?	?	?	1/2	?	1	?	?	?	?	?	?	?	?	?	1/2	?	?	1	?	1	?	0	1	?
*Vescornis*	?	0	0	0	1	?	1	?	2	1	?	1	?	?	0	1	?	2	?	?	2	?	?	?	0	0	?	0	1	?
*Vorona*	?	?	?	?	?	?	?	?	?	?	?	?	?	?	?	?	?	?	?	?	?	?	?	?	?	?	?	?	?	?
*Schizooura*	?	1	?	0	1	?	0	?	1/2	1	?	0	0	?	0	?	?	3/4/5	0	1	1	1/2	?	0	0	0	?	0	1	0
*Jianchangornis*	1	0	?	0	1	?	?	?	1	?	?	?	?	?	0	2	0	3/4	0	?	2	?	?	?	?	?	?	0	1	0
*Archaeorhynchus*	0	0	0	?	?	1	?	?	1/2	1	?	?	?	?	0	?	?	?	?	?	2	1	1	?	?	?	1/2	0	1	0
*Longicrusavis*	1	0	?	0	1	?	?	?	1/2	1	?	1	1	?	0	2	0	?	?	?	?	?	?	?	?	?	?	?	1	0
*Apsaravis*	?	1	?	?	?	?	?	0	2	1	?	1	?	2	0	0	0	4	0	?	2	1	?	?	?	?	?	?	1	0
*Hongshanornis*	1	0	?	0	?	?	?	?	0	?	?	?	?	?	0	2	?	3/4/5	?	?	1/2	?	?	?	?	0	1	0	1	0
*Yanornis*	1	?	?	0	1	?	?	?	2	1	?	?	?	?	0	2	0	3	0	?	1/2	?	?	?	?	?	?	0	1	0
*Patagopteryx*	?	?	?	0	0	0	0	1	2	1	1	0	1	1	0	0	0	3	1	1	?	2	?	?	?	?	?	?	1	0
*Yixianornis*	1	1	?	?	?	?	?	0	1	?	1	?	?	2	0	1	0	3	?	?	2	?	1	0	0	0	1/2	0	1	0
*Gansus yumenensis*	?	?	?	?	?	?	?	1	0	1	?	1	0	2	0	1	0	4	0	0	2	2	1	0	0	0	0	1	1	0
*Gansus zheni*	1	0	1	0	1	?	?	?	?	1	1	0	?	?	0	2	?	?	0	?	2	?	1	0	0	0	0	0	1	?
*Ichthyornis*	?	0	1	0	1	1	1	1	0/1	1	1	0	1	2	0	2	0	4	0	0	2	1	1	0	0	0	?	?	1	0
*Hesperornis*	1	0	1	0	1	0	?	0	2	1	0	0	1	2	1	2	0	5	0	1	1	2	1	0	0	0	1	?	0	1/2
*Parahesperornis*	1	?	?	0	1	0	?	0	2	1	0	0	1	2	1	2	0	5	0	1	1	2	1	0	?	0	1	?	0	1/2
*Vegavis*	?	?	?	?	?	?	?	0	2	?	1	?	?	?	1	2	?	5	?	?	?	1	?	?	?	?	?	?	1	0
*Anas*	0	0	1	0	1	1	1	0	2	1	1	0	1	2	1	0	0	5	0	1	2	2	1	0	0	0	2	1	1	0
*Gallus*	0	0	1	0	1	1	1	0	2	1	1	0	1	2	1	0	0	5	0	1	2	2	1	0	0	0	2	1	1	2
*Bellulornis*	?	?	?	?	?	?	?	?	?	?	?	?	?	?	?	0	0	2/3/4	?	?	2	*1*	?	0	0	0	?	?	1	0
*Fortunguavis*	?	?	?	?	0	?	?	?	0	?	1	1	0	1/2	0	1	?	0	0	?	2	?	?	1	?	1	0	0	1	1
*Qiliania*	?	?	?	?	?	?	?	?	?	?	?	?	?	?	?	?	?	?	?	?	?	?	?	?	?	?	?	?	?	?
*shenqiornis*	0	0	?	?	1	?	0	?	0/1	1	1	0	0	?	0	1	1	?	?	?	?	?	?	?	?	?	?	0	1	?
*Sulcavis*	0	?	?	?	?	?	?	?	?	?	?	1	1	?	?	1	1	1/2	?	?	2	1	0	1	1	0	?	0	1	?
*Bohaiornis*	0	0	?	0	0	1	?	?	?	?	1	1	1	?	0	1	1	0/1/2	?	?	2	?	0	1	1	0	1	0	1	1
*Parabohaiornis*	0	0	?	0	?	?	?	?	?	0	1	?	?	1	0	1	1	1	?	?	2	?	0	1	1	0	1	0	1	?
*Longusunguis*	0	0	?	0	1	?	?	?	1/2	?	1	?	?	1	0	1	1	1/2	?	?	2	0	0	1	1	0	?	0	1	?
*Zhouornis*	0	0	0	0	?	?	1	?	1/2	?	1	1	?	?	0	1	1	1	?	?	2	0	0	1	1	0	?	0	1	1
CUGB P1202	0	0	?	?	?	?	?	?	1/2	?	?	1	?	?	?	1/2	?	1/2	?	?	2	?	?	?	?	0	?	0	1	?
*Gretcheniao*	?	0	?	0	0	?	?	?	1/2	?	?	1	0	1	0	1	1	?	?	?	2	?	1	?	?	0	?	?	1	1

Taxon\Character	61	62	63	64	65	66	67	68	69	70	71	72	73	74	75	76	77	78	79	80	81	82	83	84	85	86	87	88	89	90
*Archaeopteryx*	0	0	0	0	0	0	0	0	0	0	0	?	0	0	0	0	0	0	0	0	0	0	0	0	0	?	?	?	?	?
*Jeholornis*	0	1	?	0	0	0	0	1	?	0	1	?	1	1	1	0	?	1	?	1	0	?	0	0	1/2	0/1	?	?	?	?
*Sapeornis*	0	1	1	0	0	0	0	0	?	0	0	?	0	1	1	0	?	?	?	0	0	0	0	2	?	?	?	?	?	?
*Confuciusornis*	0	?	0	?	0	0	0	?	?	0	0	?	0	0	1	0	1/2	1	0	0	0	0	0	0	2	1/2	1	0	?	?
*Changchengornis*	0	?	0	?	0	0	0	?	?	?	?	?	0	0	1	0	?	?	?	?	0	0	0	1	2	1	?	?	?	?
*Eoconfuciusornis*	0	?	?	?	0	0	0	0/1	0	?	0	?	0	?	1	0	0	?	1	0	0	0	0	0	?	?	?	?	?	?
*Protopteryx*	1	1	?	0	1	1	?	?	?	0	1	?	2/3	1	0	0	?	1	?	0	1	1	1	3	2	1/2	1	1	1	2
*Orienantius*	?	1	?	0	?	0	?	1	?	1	1	?	?	1	0	0	?	0	0	0	1	1	?	3	2	2	1	2	0	2
*Eopengornis*	*1*	1	1	0	1	0	0	?	?	1	1	?	?	1	0	*0*	1	1	0	0	*1*	1	1	3	2	1	?	1	1	0
*Parapengornis*	1	1	*1*	0	1	*0*	0	?	0	0	1	?	?	1	0	?	1	1	?	?	1	1	1	3	2	1/2	?	*1*	*2*	0
*Junornis*	1	?	?	0	?	?	?	1	?	1	2	?	0	1	0	0	1/2	0	?	1	1	1	?	2/3	2	?	?	2	1	3
*Concornis*	1	1	1	0	?	0	?	1	1	1	2	?	0	1	0	?	3	1	?	?	1	1	1	2/3	2	2	1	2	2	2
*Elsornis*	1	1	1	0	0	0	1	2	1	1	2	?	0	1	0	0	1	0	1	0	1	1	1	2/3	2	2	1	1	0	?
*Eoalulavis*	1	1	1	0	1	0	1	1	1	1	1	0	0	1	0	0	2	0	?	1	1	1	?	3	1	2	?	0	?	?
*Cathayornis*	1	1	1	0	1	0	?	?	?	1	1	?	0	1	0	0	1	0	0	1	1	1	?	3	2	2	1	2	2	3
*Eocathayornis*	1	?	?	?	?	0	?	?	?	1	2	?	0	1	0	0	1	0	?	?	?	?	?	?	2	2	1	2	1	3
*Eoenantiornis*	1	1	1	0	1	0	1	1	1	0	1	0	0	1	0	?	2/3	0	?	?	1	1	1	2/3	2	1/2/3	?	2	0	2/3
*Gobipteryx*	1	1	?	0	1	0	1	1	1	?	1/2	0	0	1	?	?	0	0	?	?	1	1	?	2/3	?	?	?	?	?	?
*Iberomesornis*	1	?	?	0	?	0	?	?	?	1	1	?	0	1	?	?	?	?	?	?	1	1	?	2/3	1/2	1/2/3	?	?	?	?
*Longipteryx*	1	1	1	0	1	0	?	?	?	1	1/2	?	0	1	0	0	1	0	?	1	1	1	1	3	2	2/3	?	2	1	1
*Longirostravis*	1	?	?	?	?	0	?	2	?	1	1	?	0	1	0	0	1/2/3	0	?	0	1	1	1	1/2/3	2	1/2/3	?	2	1	4
*Neuquenornis*	1	?	?	?	?	0	1	1	1	2	1/2	1	0	1	0	?	?	?	1	?	1	1	1	1	2	3	0	1/2	?	1
*Pengornis*	1	1	?	?	?	0	?	?	?	1	1	?	?	1	0	?	2	?	?	?	1	1	?	2	?	?	?	?	2	1
*Rapaxavis*	1	?	?	0	?	0	0	?	?	0	1	?	0	1	?	?	2	0	?	?	1	1	0	3	2	2	1	2	2	4
*Shanweiniao*	1	?	?	?	?	0	0	?	?	1	1	?	0	1	0	0	2	0	0	0	1	1	?	2/3	2	1/2/3	?	2	1	1
*Vescornis*	1	?	?	0	?	0	?	1/2	?	0/1	1	?	0	1	0	0	2	0	?	0	1	1	?	3	2	2	1	2	0	1
*Vorona*	?	?	?	?	?	?	?	?	?	?	?	?	?	?	?	?	?	?	?	?	?	?	?	?	?	?	?	?	?	?
*Schizooura*	1	1	?	0	?	1	0	1	?	1	1	?	0	1	1	?	?	0	?	?	1	1	0	1	2	2	?	2	?	0
*Jianchangornis*	1	1	1	0	0	1	0	2	0	0	1	?	0	1	1	0	0	0	0	1	2	1	0	0	2	2/3	0	2	1	0
*Archaeorhynchus*	1	1	?	0	0	1	?	2	0	0	1	?	0	1	1	0	0	1	0	?	2	1	0	0	2	2/3	0	2	2	2
*Longicrusavis*	1	1	1	0	0	1	?	?	?	0	?	?	1/2	1	1	0	0	1	0	1	2	1	0	1	2	3	0	1	2	0
*Apsaravis*	1	1	1	0	0	0	1	1	1	1	2	1	1	1	1	1	?	1	0	1	?	?	?	?	1/2	2/3	0	?	?	?
*Hongshanornis*	1	1	1	0	0	?	0	?	?	0	2	?	1	1	1	1	0	1	0	1	2	1	?	1	2	1/2/3	?	2	2	?
*Yanornis*	1	1	1	1	0	1	0	?	0	0	1	?	2	1	1	0	?	1	?	1	2	1	0	0	2	3	0	2	1/2	2
*Patagopteryx*	1	1	1	0	0	0	0	?	0	?	?	?	1	1	1	1	1	0	?	0	?	?	?	?	2	1/2	?	?	?	?
*Yixianornis*	1	1	1	0	0	1	0	1	0	0	1	?	2	1	1	1	0	1	0	1	2	1	0	?	2	3	0	1	1	2
*Gansus yumenensis*	1	1	1	0	0	1	0	2	0	0	1	?	3	1	1	0	1	1	1	1	2	1	0	0	2	3	0	2	1	0
*Gansus zheni*	1	1	?	0	0	1	?	2	0	0	1	?	3	1	1	0	1	1	?	?	2	1	0	0	2	3	0	2	1	1
*Ichthyornis*	1	1	1	1	0	1	0	1	0	0	1	?	2	1	1	1	0	1	0	1	2	?	0	0	2	3	0	0	?	?
*Hesperornis*	0	1	?	0	0	?	0	1	0	0	1	?	0	1	1	0	?	?	0	0	?	0	0	0	2	1	?	0	?	?
*Parahesperornis*	0	1	?	0	0	?	0	1	0	0	1	?	0	1	?	?	?	?	?	?	?	?	?	?	2	1	?	0	?	?
*Vegavis*	1	1	?	0	0	1	0	1	?	0	1	?	0/1	1	0	0	0	?	?	?	?	?	?	?	?	?	?	?	?	?
*Anas*	1	1	1	1	0	1	0	2	0	1	2	?	2	1	1	0	0	1	0	1	2	1	0	0	2	3	1	1	1	0
*Gallus*	1	1	1	1	0	1	0	2	0	1	2	?	2	1	1	1	0	1	0	1	2	1	0	1	2	3	1	1	0	1
*Bellulornis*	1	*1*	?	0	?	1	?	1	0	0/1	1	?	0	1	1	?	?	?	0	?	1	1	0	1	2	2/3	1	1	2	2
*Fortunguavis*	1	1	1	0	1	0	1	1	1	0	?	0	0	1	1	0	?	?	0	0	?	?	1	2	2	2/3	?	2	1/2	?
*Qiliania*	?	?	?	?	?	?	?	?	?	?	?	?	?	?	?	?	?	?	?	?	?	?	?	?	?	?	?	?	?	?
*shenqiornis*	1	1	?	0	?	0	?	1/2	?	0/1	1	?	0	1	0	0	2	1	?	0	1	1	?	2/3	2	?	?	1/2	1	2
*Sulcavis*	1	?	?	0	?	0	?	?	?	1	1	?	0	1	0	0	2	0	0	?	1	1	1	3	1/2	?	?	?	?	?
*Bohaiornis*	1	1	1	0	?	0	0	?	0	0	2	?	0	1	0	0	2	0	0	1	1	1	1	2	2	2	1	2	1	3
*Parabohaiornis*	1	1	1	0	?	0	?	1	1	0	2	?	0	1	0	0	2	0	0	1	1	1	1	3	2	1/2	?	2	0	3
*Longusunguis*	1	?	?	0	?	?	0	?	?	0	2	?	0	1	0	0	2	0	0	1	1/2	1	?	3	2	1/2	?	?	?	?
*Zhouornis*	1	1	1	0	1	0	0	1	1	1	2	?	0	1	1	0	1/2	0	0	1	1	1	1	3	2	2	1	2	1	3
CUGB P1202	1	?	?	0	?	0	?	1	?	1	1	?	0	1	0	0	?	0	?	?	1	1	1	2/3	2	2	1	2	1	2/3/4
*Gretcheniao*	1	1	1	0	1	0	?	1	1	2	2	?	0	1	0	0	0/1/2	0	0	?	1	1	1	3	2	2	1	2	1	1/2

Taxon\Character	91	92	93	94	95	96	97	98	99	100	101	102	103	104	105	106	107	108	109	110	111	112	113	114	115	116	117	118	119	120
*Archaeopteryx*	?	?	?	?	?	?	0	0	0	?	0	0	0	0	0	0	0	0	0	0	0	0	0	0	0	0	0	0	?	0
*Jeholornis*	?	?	?	0	0	0	?	0	0	0	0	0	0	0	0	0	0	0	?	0/1	0	0	?	?	0	1	0	1	1	1
*Sapeornis*	?	?	?	?	?	?	0	0	0	?	?	0	0	0	1	0	1	0	0	0	0	0	0	0	0	1	0	0	1	1
*Confuciusornis*	0	1	1	0	0	?	0	0	0	0	1	0	0	0	2	0	1	0	?	0	0	0	0	0	0	1	0	0	0	0
*Changchengornis*	?	?	?	0	?	?	?	?	0	?	?	?	?	0	2	0	?	?	?	?	?	?	?	?	0	?	?	?	?	0
*Eoconfuciusornis*	?	?	?	?	?	?	0	0	0	0	0	0	0	0	1	0	0	1	?	?	?	?	?	?	0	?	?	?	?	0
*Protopteryx*	0	1	?	0	?	0	?	?	0	?	?	0	0	0	0	1	0	?	?	?	?	?	?	?	0	?	?	?	?	1
*Orienantius*	1	?	?	0	?	0	?	?	0	?	?	?	?	0	0	1	0	0	?	?	?	?	?	?	?	?	0	?	?	1
*Eopengornis*	?	1	?	0	?	0	0	0	*1*	*0*	*0*	1	*0*	*0*	0	0	0	?	?	0	?	0	0	0	0	?	?	?	?	1
*Parapengornis*	1	0/1	0	0	?	0	0	0	1	?	*0*	*1*	*0*	0	0	0	0	?	?	0	?	0	0	0	0	?	?	?	?	1
*Junornis*	1	1	?	0	?	1	0	0	1	?	?	1	?	0	0	1	0	2	?	?	?	?	?	?	1	?	?	?	?	1
*Concornis*	1	1	1	0	1	0	0	0	1	1	1	1	?	0	0/1	1	0	2	1	?	?	?	?	?	?	1	?	?	?	?
*Elsornis*	1	1	0	0	?	1	0	0	1	1	0	0	0	0	0	1	0	0	1/2	1	0	0	0	0	1	1	1	?	?	0
*Eoalulavis*	0	?	0	0	?	?	0	0	1	1	1	1	0	0	0	1	0	2	1	0	0	1	0	0	1	1	1	1	1	1
*Cathayornis*	1	1	0	0	?	0	0	0	1	1	1	1	?	0	1	1	0	2	1	0/1	0	1	0	1	1	1	1	1	0	1
*Eocathayornis*	1	1	?	0	?	0	?	0	1	?	?	1	?	0	0	1	0	?	?	0/1	?	1	1	?	1	1	?	?	?	1
*Eoenantiornis*	1	1	0	0	0	0	0	?	1	?	?	1	?	0	0	1	0	?	?	?	?	?	?	?	?	?	?	?	?	1
*Gobipteryx*	?	?	?	?	?	?	0	?	?	?	?	?	?	0	?	?	?	?	?	?	?	?	?	?	?	?	?	?	?	1
*Iberomesornis*	?	?	?	?	?	?	?	?	?	?	0	?	?	0	?	?	?	0/1	?	?	0	?	?	?	1	?	?	?	?	1
*Longipteryx*	1	1	?	0	?	1	?	0	1	1	0	1	?	0	0	1	0	2	?	0/1	0	0	0	0	1	1	?	1	1	1
*Longirostravis*	1	1	0	0	?	0	?	0	1	?	?	1	?	0	0	1	0	?	?	0/1	?	?	?	?	1	?	?	?	?	1
*Neuquenornis*	?	1	?	?	?	?	0	?	?	?	?	1	1	0	?	0	?	?	?	?	0	1	?	1	1	?	?	?	?	1
*Pengornis*	?	?	?	?	?	?	0	0	1	?	0	1	0	0	1	0	0	1	?	0/1	?	1	0	0	0	1	?	0	?	1
*Rapaxavis*	1	1	?	0	?	0	0	0	1	0	0	?	?	0	0	1	0	1	?	0/1	0	?	?	?	1	1	1	1	1	1
*Shanweiniao*	?	0/1	?	0	?	0	?	?	1	?	?	?	?	?	0	1	0	?	?	?	?	?	?	?	1	1	?	?	?	1
*Vescornis*	1	1	?	0	?	0	1	0	1	1	0	1	?	0	1	1	0	1	?	0/1	0	?	?	?	1	1	1	1	1	1
*Vorona*	?	?	?	?	?	?	?	?	?	?	?	?	?	?	?	?	?	?	?	?	?	?	?	?	?	?	?	?	?	?
*Schizooura*	1	1	?	?	?	?	1	?	0	0	0	?	?	0	1	0	0	0	1	0/1	0	?	?	?	0	1	?	0	?	1
*Jianchangornis*	0	1	?	0	?	1	0	1	0	0	0	0	0	0	1	1	0	0	1	0	0	?	?	?	0	1	?	0	1	1
*Archaeorhynchus*	1	1	0	0	?	0	0	1	0	0	0	?	?	0	1	1	0	?	?	?	0	?	?	?	0	1	1	0	?	1
*Longicrusavis*	1	1	0	0	?	0	1	1	0	1	0	1	?	0	1	0	0	2	2	2	1	1	0	0	0	1	0	0	0	1
*Apsaravis*	1	1	?	?	?	?	1	1	0	0	1	0	0	0	1	0	0	1	1	0	0	0	0	1	1	1	1	1	1	1
*Hongshanornis*	1	1/2	?	0	?	0	1	1	0	?	?	1	?	0	1	1	0	?	?	2	?	0	0	0	0	?	?	?	?	1
*Yanornis*	1	1/2	?	1	0	0	1	1	0	0	1	1	?	0	1	1	0	2	1	0/1	0	?	?	?	0	1	0	0	0	1
*Patagopteryx*	1	0/1	?	?	?	?	1	0	0	1	?	?	?	1	0	1	0	0	?	?	0	0	0	0	0	1	0	0	?	0
*Yixianornis*	1	1/2	0	1	?	1	?	1	0	?	?	1	1	?	?	1	0	?	?	1	?	1	0	?	0	1	?	0	?	1
*Gansus yumenensis*	1	0	0	1	?	0	1	1	0	0	1	1	?	0	1	1	0	1	3	?	1	0	0	0	0	1	0	0	1	1
*Gansus zheni*	1	?	?	?	?	0	1	1	0	0	1	?	?	0	1	1	0	2	2/3	?	1	?	?	?	0	1	?	0	?	1
*Ichthyornis*	1	2	1	0	1	?	1	1	0	0	1	1	0	0	1	0	0	1	2	2	1	0	0	0	0	1	0	0	0	1
*Hesperornis*	0	0	1	0	0	?	?	?	?	?	?	?	0	?	?	?	?	0	0	0	0	0	0	0	0	?	?	?	?	?
*Parahesperornis*	0	0	1	0	0	?	?	?	?	?	?	?	0	?	?	?	?	0	0	0	0	0	0	0	0	?	?	?	?	?
*Vegavis*	?	?	?	?	?	?	1	1	0	?	?	1	1	1	0	?	?	0	?	?	?	?	?	?	?	?	?	?	?	?
*Anas*	0	1	1	0	1	1	1	1	0	0	1	1	1	1	0	1	0	0	3	1	1	1	1	1	0	1	0	0	0	0
*Gallus*	0	1	1	0	1	1	1	1	0	0	1	1	1	1	0	1	0	0	3	1	1	1	1	1	1	1	0	0	0	1
*Bellulornis*	1	1	0	0	?	0	1	1	0	0	0	0	?	0	1	0	0	0	1	?	?	?	?	?	0	?	?	?	?	1
*Fortunguavis*	1	?	?	0	0	0	0	0	0	?	?	1	0	0	0	1	0	?	?	?	?	0	0	0	1	?	?	?	?	?
*Qiliania*	?	?	?	?	?	?	?	?	?	?	?	?	?	?	?	?	?	?	?	?	?	?	?	?	?	?	?	?	?	?
*shenqiornis*	?	1	?	0	?	0	?	0	1	1	0	1	?	0	0	0	0	0	?	?	?	?	?	?	?	1	?	?	?	1
*Sulcavis*	?	?	?	?	?	?	?	0	1	?	?	?	?	0	0/1	0	0	?	?	?	?	?	?	?	?	1	?	?	?	1
*Bohaiornis*	1	1	?	0	?	1	0	0	1	?	?	1	0	0	0	1	0	1	?	0	?	?	1	?	1	1	?	?	?	1
*Parabohaiornis*	1	1	?	0	?	1	0	0	1	?	?	1	0	0	0	1	0	1	0	0	1	?	?	?	1	1	1	0	0	1
*Longusunguis*	1	?	?	0	?	?	0	0	1	?	?	1	0	0	0	0	0	?	?	0	?	0	?	?	1	1	?	?	?	1
*Zhouornis*	1	1	0	0	?	1	0	0	0	?	?	1	0	0	0	0	0	?	?	0	?	1	1	1	1	1	?	?	?	1
CUGB P1202	?	?	?	0	?	0	?	?	0	?	?	?	?	0	0	1	0	?	?	?	?	?	?	?	?	?	?	?	?	1
*Gretcheniao*	1	?	?	0	?	1	?	?	0	0	0	?	0	0	0	1	0	1	1	?	0	?	0	?	?	1	1	0	0	1

Taxon\Character	121	122	123	124	125	126	127	128	129	130	131	132	133	134	135	136	137	138	139	140	141	142	143	144	145	146	147	148	149	150
*Archaeopteryx*	0	0	0	0	0	0	0	?	0	0	1	0	0	0	0	0	0	0	0	0	0	0	0	0	0	0	0	0	0	2
*Jeholornis*	1	0	?	0	0	0	0	0	2	1	1	0	0	1	0	0	0	0	1	0	0	0	2	0	0	0	1	0	1	3
*Sapeornis*	1	?	?	?	?	0	0	?	2	1	1	0	0	0	0	0	0	0	0	?	0	0	2	0	1	0	1	1	0	3
*Confuciusornis*	1	0	0	2	1	0	0	0	2	1	0	?	0	0	0	0	1	0	0	?	0	0	0	0	0	0	2	0	0	2
*Changchengornis*	1	?	?	?	?	?	0	?	?	?	?	?	0	0	?	0	0	0	?	?	0	0	0	0	1	0	2	0	0	2
*Eoconfuciusornis*	1	?	?	?	?	?	0	?	0	1	?	?	0	0	0	?	0	?	0	?	0	0	0	1	0	0	2	0	0	2
*Protopteryx*	1	?	?	?	1	1	0	0	3	1	?	?	0	0	0	0	0	0	0	0	0	1	1	?	0	0	0	1	0	2
*Orienantius*	1	?	?	?	?	1	0	?	0	1	?	?	0	0	1	0	1	0	0	?	0	1	2	0	1	0	0	1	0	2
*Eopengornis*	1	?	?	?	?	?	*?*	*0*	?	1	0/1	?	0	0	?	0	?	0	0	1	?	1	1	0	1	0	2	?	0	3
*Parapengornis*	1	*1*	?	?	?	?	?	0	?	1	*1*	0	0	0	1	0	0	0	0	1	?	1	1/2	0	1	0	2	1	1	3
*Junornis*	1	?	?	?	?	?	?	0	2	1	?	?	0	?	?	0	1	0	0	?	?	1	2	0	1	1	0	1	1	2
*Concornis*	1	1	1	2	?	?	?	?	?	?	?	?	?	?	?	1	?	?	0	?	?	?	?	0	1	0	?	?	2	2
*Elsornis*	1	0	0	1/2	1	?	1	?	3	1	?	?	?	?	?	?	?	?	?	0	1	?	?	?	?	?	?	?	?	?
*Eoalulavis*	1	?	?	2	1	?	1	?	?	?	?	?	?	?	?	0	?	?	0	?	0	1	?	0	1	?	?	?	2	?
*Cathayornis*	1	?	?	?	?	1	1	0	?	1	1	0	0	0	1	0	1	0	0	0	0	1	3	0	1	0	1	1	2	2
*Eocathayornis*	0	?	?	?	?	?	?	0	3	1	?	?	0	0	1	0	1	?	0	0	0	1	2	0	1	0	0	0	1	?
*Eoenantiornis*	1	?	?	?	?	?	1	0	3	1	?	?	0	0	0	0	1	0	0	0	0	1	2	0	1	0	1	1	2	2
*Gobipteryx*	1	?	?	?	1	?	1	?	?	?	?	?	?	?	?	0	?	?	0	?	0	1	?	0	?	?	?	?	?	?
*Iberomesornis*	1	?	?	?	?	?	?	?	?	?	?	?	?	?	?	?	?	?	?	?	?	?	?	?	?	?	?	?	?	?
*Longipteryx*	1	?	?	?	?	?	?	0	0	1	1	0	0	0	0	1	1	0	0	0	0	1	2	0	1	0	0	0	1	3
*Longirostravis*	0	?	?	?	?	?	?	?	3	1	?	?	1	0	?	?	?	?	0	1	0	1	2	0	1	?	?	?	?	3
*Neuquenornis*	1	?	1	?	?	1	1	?	3	1	?	?	1	0/1	?	0	?	?	0	0	?	?	?	?	?	?	?	?	?	?
*Pengornis*	1	?	?	?	?	?	?	1	2/3	1	1	1	?	?	?	0	?	0	1	1	0	1	?	0	1	?	?	?	1/2	3
*Rapaxavis*	0	?	?	?	1	?	?	0	0/1/2	1	1	?	0	0	0	0	1	0	0	0	0	1	3	0	1	1	?	1	2	3
*Shanweiniao*	1	?	?	?	?	1	?	?	?	?	?	?	?	?	?	?	?	?	?	?	?	?	2	0	1	1	?	?	2	2
*Vescornis*	1	?	?	?	?	?	?	1	0	1	1	0	0	0	?	0	1	?	0	?	0	1	2/3	0	1	0	0/1	1	2	2
*Vorona*	?	?	?	?	?	?	?	?	?	?	?	?	?	?	?	?	?	?	?	?	?	?	?	?	?	?	?	?	?	?
*Schizooura*	0	?	?	?	?	?	0	0	3	1	?	?	0	0	0	0	1	0	0	1	?	0	2	0	1	0	1	1	0	3
*Jianchangornis*	1	?	?	?	?	?	0	?	?	1	?	?	0	1	?	1	0	0	0	0	?	?	0/1	1	1	0	1	0	1	3
*Archaeorhynchus*	1	?	?	?	?	1	0	0	?	1	?	?	0	0	0	1	1	0	0	1	0	0	2	1	1	0	1	?	2	3
*Longicrusavis*	1	0	0	?	?	?	0	?	3	1	1	1	0	?	?	1	0	0	0	0	1	0	2	1	0	0	1	1	0	1
*Apsaravis*	1	0	1	2	1	1	0	0	3	1	1	0	0	1	1	1	?	1	1	0	?	?	?	1	?	?	?	?	?	?
*Hongshanornis*	1	?	?	?	?	?	?	?	3	1	?	?	0	1	?	1	0	?	0	0	1	0	2	1	1	0	1	1	0	1
*Yanornis*	1	?	0	?	1	1	?	0	3	1	1	1	0	0	0	1	1	0	0	0	1	0	2	1	1	0	1	?	2	3
*Patagopteryx*	0	0	0	?	0	?	0	?	1/2/3	?	?	?	?	?	?	?	?	?	1	?	1	?	?	0	0	0	?	?	2	0
*Yixianornis*	0	0	?	2	1	1	0	0	3	1	1	1	0	1	0	1	1	0	1	0	1	0	2	1	1	0	1	1	0	2
*Gansus yumenensis*	1	0	0	2	1	1	1	0	3	1	2	1	0	1	1	1	1	0	0	1	1	0	3	1	1	0	0	1	0	2
*Gansus zheni*	1	?	0	2	?	?	0	?	3	1	2	1	0	0	1	1	1	0	0	1	1	0	2	1	1	0	1	1	1	2
*Ichthyornis*	1	0	0	2	1	1	0	1	3	1	2	1	0	1	2	1	1	1	0	0	1	0	3	1	1	1	?	?	?	3
*Hesperornis*	?	?	?	?	?	?	?	?	?	?	?	?	?	?	?	?	?	?	?	?	?	?	?	?	?	?	?	?	?	?
*Parahesperornis*	?	?	?	?	?	?	?	?	?	?	?	?	?	?	?	?	?	?	?	?	?	?	?	?	?	?	?	?	?	?
*Vegavis*	0	?	?	?	?	?	0	1	3	1	2	?	0	1	1	1	1	?	1	0	?	0	3	1	1	?	?	?	2	?
*Anas*	1	0	0	1	1	1	0	1	3	1	2	0	1	2	2	1	1	1	1	1	1	0	3	1	1	1	?	1	2	3
*Gallus*	1	0	0	1	1	1	0	1	3	1	2	0	1	2	2	1	1	1	1	0	1	1	3	1	1	1	?	1	2	1
*Bellulornis*	0	?	?	?	?	?	?	0	3	1	0	0	0	0	0	0	0	0	0	0	1	0	2	0	1	0	0	1	0	3
*Fortunguavis*	1	?	?	?	?	?	1	1	2	1	1	0	0	0	0	0	1	0	0	1	0	1	2	0	1	0	0	?	1	?
*Qiliania*	?	?	?	?	?	?	?	?	?	?	?	?	?	?	?	?	?	?	?	?	?	?	?	?	?	?	?	?	?	?
*shenqiornis*	1	?	?	?	?	?	0	?	0	1	?	?	0	0	0	0	1	0	0	1	0	1	2	0	1	0	0	?	1/2	2/3
*Sulcavis*	1	?	?	0/1	?	?	?	0	0/2	1	?	?	0	0	0/1	?	1	0	?	?	0	1	2	0	1	0	1	1	1	2
*Bohaiornis*	1	?	?	?	?	?	1	0	0	1	1	0	0	0	0	1	1	0	0	?	0	1	2	0	1	0	1	1	2	3
*Parabohaiornis*	1	?	?	?	?	?	1	0	?	1	?	?	0	0	0	1	1	0	0	1	?	1	2	0	1	0	1	1	2	3
*Longusunguis*	1	?	?	?	?	?	1	0	0	1	?	?	0	0	0	1	?	0	0	1	?	1	?	0	1	0	1	1	2	2
*Zhouornis*	1	?	?	?	?	?	1	0	3	1	?	?	0	0	0	0	0	0	0	?	0	1	2	0	1	0	1	?	2	2
CUGB P1202	1	?	?	?	?	?	1	0	?	1	?	?	0	0/1	?	0	1	?	0	?	0	1	2	0	1	0	0/1	1	2	?
*Gretcheniao*	1	1	?	?	?	1	1	0	2	1	1	0	0	0	?	0	1	0	0	1	0	1	2	0	1	0	?	1	1	2/3

Taxon\Character	151	152	153	154	155	156	157	158	159	160	161	162	163	164	165	166	167	168	169	170	171	172	173	174	175	176	177	178	179	180
*Archaeopteryx*	0	0	0	0	0	0	1	?	0	0	1	0	0	0	0	2	?	1	0	?	0	0	0	0	0	0	0	1	1	?
*Jeholornis*	0	0	0	0	0	0	1	1	?	1	1	0	0	0	0	1	?	0	?	0/1	0	?	?	0	0	?	0	1	0	0/1
*Sapeornis*	0	0	0	?	0	?	1	1	0	1	1	1	?	0	0	1	0	1	0	?	?	0	1	0	1	0	0	1	0	?
*Confuciusornis*	0	0	0	0	0	0	?	1	0	1	1	1	0	0	0	0	1	0	1	1	2	0	2	1	1	?	0	0	0	1/2
*Changchengornis*	?	?	0	?	?	?	?	?	?	?	?	1/2	?	?	0	?	?	?	?	?	?	?	?	?	?	?	0	1	?	?
*Eoconfuciusornis*	?	?	0	?	0	?	?	?	?	?	?	?	0	0	0	1/2	?	?	?	?	?	1	?	?	0	?	?	1	?	?
*Protopteryx*	?	?	?	?	?	?	1	?	1	?	?	?	?	?	0	1	?	?	?	?	?	?	?	?	?	?	?	1	?	?
*Orienantius*	0	0	0	?	0	?	1	?	?	?	1	?	?	?	0	0	?	?	?	?	2	?	?	?	0	?	0	1	?	?
*Eopengornis*	?	?	?	?	?	?	?	?	?	?	?	?	?	?	0	0/1/2	?	?	?	?	?	?	?	?	?	?	?	1	*0*	*0*
*Parapengornis*	?	0	0	?	?	?	*0*	?	*1*	*0*	?	?	0	*1*	0	2	?	?	?	?	?	?	*0*	?	?	?	0	1	*0*	*0*
*Junornis*	0	0	0	?	?	?	?	?	0	?	1	1/2	0	?	?	?	?	?	?	?	?	?	?	?	1	?	0	1	0	?
*Concornis*	?	?	?	?	?	?	?	?	1	0	1	1/2	?	0	0	?	0	?	?	?	?	0	?	?	1	1	1	2	1	?
*Elsornis*	?	?	?	?	?	?	?	?	?	?	?	?	?	?	?	?	?	?	?	?	?	?	?	?	?	?	?	?	?	?
*Eoalulavis*	?	?	?	?	?	?	1	?	?	?	?	?	?	?	?	?	?	?	1	?	1	?	?	?	?	?	?	?	?	?
*Cathayornis*	0	0	0	0	?	?	1	1	?	?	?	?	0	0	?	0	?	0	1	2	1	?	1/2	1	1	?	0	0	0/1	?
*Eocathayornis*	?	?	?	?	?	?	?	?	?	?	?	?	?	?	?	?	?	?	?	?	?	?	?	?	?	?	?	?	?	?
*Eoenantiornis*	?	?	0	?	?	?	?	?	?	?	?	1/2	?	0	0	1	?	?	?	?	1	?	?	?	?	?	?	1	?	?
*Gobipteryx*	0	?	0	?	?	?	?	?	?	?	?	?	0	?	0	3	?	?	?	?	?	?	?	?	0	1	0	?	1/2	1
*Iberomesornis*	?	?	?	?	?	?	?	?	?	?	?	1	?	0	?	?	?	?	?	?	?	?	?	?	0	1	0	1	?	?
*Longipteryx*	0	0	0	?	?	?	1	1	1	0	1	1/2	1	0	0	1	?	0	?	1/2	1	?	?	?	?	?	0	2	?	?
*Longirostravis*	?	?	?	?	1	?	1	?	?	1	1	?	1	?	?	?	?	0	?	1	?	?	?	?	1	?	0	1	0	?
*Neuquenornis*	?	?	?	?	?	?	?	?	?	?	?	?	?	?	?	?	?	?	1	1	1	0	?	1	?	?	?	?	1	?
*Pengornis*	?	?	?	?	?	?	?	?	?	?	?	?	?	?	0	2	?	0	1	1	?	?	?	?	1	?	0	1	0/1	?
*Rapaxavis*	0	0	0	0	?	?	1	1	1	1	1	1/2	1	0	0	?	0	0	?	?	?	?	?	?	0	?	?	1	0/1	?
*Shanweiniao*	?	?	?	?	?	?	?	?	?	?	?	?	?	?	?	?	?	0	?	0	?	0	0/1	?	1	?	?	0	?	?
*Vescornis*	?	?	?	?	?	?	?	?	?	?	?	?	?	?	?	?	?	?	?	?	?	?	?	?	0	?	?	1	0/1	?
*Vorona*	?	?	?	?	?	?	?	?	?	?	?	?	?	?	?	?	1	0	1	0	2	0	2	1	0	0	0	?	1	1
*Schizooura*	?	0	?	?	?	?	?	?	0	1	1	1/2	0	1	0	3	?	?	?	?	?	1	?	?	?	?	?	1	1/2	?
*Jianchangornis*	?	?	0	?	?	?	?	?	?	?	?	1/2	0	0	0	3	?	?	?	?	?	?	?	0	?	?	0	0	?	?
*Archaeorhynchus*	?	0	0	?	?	?	1	0	1	1	0	1/2	0	0	0	1	?	?	?	?	?	0	?	?	?	?	0	0	1	0
*Longicrusavis*	?	?	?	?	?	1	1	?	?	1	0	1/2	?	0	?	?	?	?	?	?	?	?	?	1	1	0	0	1	2	2
*Apsaravis*	1	0	0	0	1	1	1	?	1	1	0	2	?	1	1	3	1	0	1	1/2	?	1	1	?	1	?	?	?	?	2
*Hongshanornis*	?	?	?	?	?	?	?	?	0	?	0	1/2	?	0	?	3	?	?	?	?	?	?	?	?	0	?	0	1	1/2	?
*Yanornis*	1	0	0	?	1	1	?	1	0	1	0	1/2	0	0	0	3	?	0	1	1	?	?	?	?	1	?	0	0	?	?
*Patagopteryx*	?	0	0	?	0	0	0	0	1	1	0	1	?	0	1	3	?	0	1	1	2	0	?	1	1	0	0	0	1	?
*Yixianornis*	1	0	?	?	?	1	0	?	0	1	1	1	?	0	0	3	?	?	1	?	2	?	1	?	1	0	0	0	1/2	2
*Gansus yumenensis*	1	0	0	1	1	1	0	1	0	1	1	2	0	0	0	3	0	0	1	1	2	1	1	1	1	0	0	1	2	2
*Gansus zheni*	1	0	?	?	1	1	?	1	0	1	0	2	0	0	0	0	?	0	1/2	1	?	?	1/2	1	1	?	0	1	2	2
*Ichthyornis*	1	0	0	0	1	1	?	?	?	1	1	2	?	?	?	?	1	0	1	0	2	1	1	1	1	0	0	1	2	2
*Hesperornis*	1	0	0	1	1	1	0	1	1	1	0	2	0	1	1	3	1	0	2	1	2	2	2	1	1	0	1	0	2	2
*Parahesperornis*	1	0	0	1	1	1	0	1	1	1	0	2	0	1	1	3	1	0	2	1	2	2	2	1	1	0	1	0	2	2
*Vegavis*	0	1	?	1	1	?	?	?	?	0	?	2	?	?	?	?	?	0	1	1	2	1	2	1	1	0	0	?	2	?
*Anas*	1	1	1	1	1	1	0	1	0	1	1	2	0	1	1	3	1	0	1	1	2	1	1	1	1	0	0	1	2	2
*Gallus*	1	1	1	1	1	1	0	1	1	0	1	2	0	1	1	3	1	0	1	1	2	1	1	1	1	0	0	1	2	2
*Bellulornis*	1	?	?	?	?	1	?	?	?	1	?	2	?	?	0	?	?	0	?	0	?	?	?	?	1	?	0	1	2	?
*Fortunguavis*	0	?	?	?	?	?	?	?	0	1	0	1	?	0	1	2	?	0	1	0	?	?	?	?	1	?	?	1	?	1
*Qiliania*	1	0	?	?	0	1	?	1	0	1	1	2	0	0	0	3	?	0	?	?	1	1	1	0	0	0	0	2	1	1
*shenqiornis*	0	0	0	?	?	?	?	?	0	1	1	0/1	?	?	0	1	?	0	?	?	?	?	?	?	0	?	?	?	?	?
*Sulcavis*	?	?	0	0	?	?	?	?	?	?	?	1/2	0	?	?	?	?	?	?	?	?	?	?	?	?	?	0	1	?	?
*Bohaiornis*	0	0	0	?	?	?	?	?	0	1	1	1/2	?	0	0	0	?	0	?	1	?	?	?	0	0	?	0	0/1	0	?
*Parabohaiornis*	?	0	0	?	0	?	?	1	0	1	1	1/2	1	0	0	0	0	0	?	?	?	?	?	?	?	?	0	0/1	0	?
*Longusunguis*	0	0	?	?	?	?	?	?	0	1	1	1/2	?	0	0	1	?	?	?	?	1	?	1	0	0	?	0	1	0	?
*Zhouornis*	0	0	0	?	0	?	1	?	?	?	?	1/2	?	0	0	1	0	0	?	?	1	?	1	0	1	?	0	1	0	?
CUGB P1202	?	?	?	?	?	?	?	?	?	?	?	1/2	?	?	?	?	?	?	?	?	?	?	?	?	0	?	?	?	?	?
*Gretcheniao*	0	0	0	?	0	?	1	1	0	1	1	1/2	0	0	?	1	?	0	?	?	1	0	1	0	0	?	0	1	0/1	?

Taxon\Character	181	182	183	184	185	186	187	188	189	190	191	192	193	194	195	196	197	198	199	200	201	202	203	204	205	206	207	208	209	210	211	212
*Archaeopteryx*	0	?	?	?	?	?	0	0	0	0	0	0	0	0	0	0	0	0	0	?	0	1	0	0	0	0	0	0	0	1	0	0
*Jeholornis*	0	?	1	0	0	?	?	1	1	0	0	0	0	0	0	?	?	0	0	?	0	2	0	0	?	0	0	0	0	0	?	0
*Sapeornis*	1	?	1	0	1	1	?	1	1	0	0	0	0	0	0	0	0	0	0	?	0	1	0	0	0	0	1/2	0	1	1	?	?
*Confuciusornis*	0	0	0	0	0	0	1	1	1	0	0	0	1	0	0	1	1	1	1	0	0	1	0	0	0	0	1	0	0	1	0	0
*Changchengornis*	?	?	?	?	?	?	?	1	1	0	0	0	?	?	0	?	?	1	?	?	0	1	0	0	?	0	1	0	0	1	?	0
*Eoconfuciusornis*	1	?	?	0	0	?	?	?	?	0	0	0	0	0	?	?	0	1	?	0	0	1/2	0	0	?	0	1	0	0	0	0	0
*Protopteryx*	?	?	?	?	?	?	?	1	?	1	?	?	?	0	?	?	?	0	?	?	0	2	?	0	?	0	0	?	0	1	1	0
*Orienantius*	?	?	?	?	?	?	?	1	0	1	?	?	?	0	?	?	?	1	?	?	0	0	?	0	?	0	2	?	0	1	1	0
*Eopengornis*	?	?	?	?	?	?	?	1	?	?	0	0	0	?	?	?	?	1	?	?	0	1	0	1	?	0	?	0	1	2	?	0
*Parapengornis*	?	*1*	*1*	?	*0*	?	?	1	?	?	0	0	?	0	?	?	?	1	?	?	*0*	*1*	?	?	?	0	1	0	1	1	?	0
*Junornis*	?	0	0	?	?	?	?	1	1	1	1	0	?	1	?	?	?	1	?	?	0	1	0	0	?	0	2	?	0	1	?	0
*Concornis*	1	1	?	?	?	?	?	1	1	1	?	0	?	0	?	?	0	0	?	?	0	0	0	0	?	0	1	?	?	?	?	?
*Elsornis*	?	?	?	?	?	?	?	?	?	?	?	?	?	?	?	?	?	?	?	?	?	?	?	?	?	?	?	?	?	?	?	?
*Eoalulavis*	?	?	?	?	?	?	?	?	?	?	?	?	?	?	?	?	?	?	?	?	?	?	?	?	?	?	?	?	?	?	1	?
*Cathayornis*	?	?	?	1	?	?	?	1	1	0/1	?	0	?	0	?	?	?	1	2	?	0	1	0	0	?	1	?	?	?	1	?	?
*Eocathayornis*	?	?	?	?	?	?	?	?	?	?	?	?	?	?	?	?	?	?	?	?	?	?	?	?	?	?	?	?	?	?	?	?
*Eoenantiornis*	?	?	?	?	?	?	?	?	1	1	?	0	?	?	?	?	?	?	?	?	0	?	?	1	?	0	?	?	0	1	1	0
*Gobipteryx*	0	?	0	1	1	?	?	1	1	0	?	0	?	0	0	0	?	1/2	?	1/2	0	?	0	1	?	1	?	?	?	?	?	?
*Iberomesornis*	?	?	?	?	?	?	?	?	0	0	1	0	0	0	?	?	0	1	?	?	0	0	0	0	?	0	?	?	0	0	?	?
*Longipteryx*	?	?	?	?	?	?	?	1	?	0	1	0	0	0	0	0	0	0	1	?	2	0	0	0	?	0	2	0	1	1	1	?
*Longirostravis*	?	?	?	?	?	1	?	1	1	0	1	0	0	0	?	?	0	?	2	?	0	1	0	0	?	0	?	?	?	?	?	?
*Neuquenornis*	?	?	?	?	?	?	?	?	1	0	?	0	?	?	?	1	?	2	?	?	0/1/3	?	0	1	1	0	?	?	?	1/2	?	?
*Pengornis*	?	1	1/2	?	1	?	?	1	1	0	1	0	0	0	0	1	0	1	1	0	0	2	0	1	?	0	1	0	0	?	?	?
*Rapaxavis*	0	?	1	?	1	?	?	1	0	0	1	0	0	0	?	?	0	0	2	?	0	1	0	0	?	0	2	0	0	1	?	?
*Shanweiniao*	?	?	?	?	?	?	?	1	1	0	?	0	0	0	0	?	?	?	1	?	0/1	1	?	0	?	0	2	?	0	1	?	1
*Vescornis*	?	?	?	?	?	?	?	?	0	0	?	0	0	0	?	?	0	1	0	?	1	2	0	1	?	1	1/2	?	0	1	?	?
*Vorona*	1	1	0	1	1	1	1	1	1	1	0	0	1	0	0	1	1	?	0	1	0	1	0	0	0	0	?	?	?	?	?	?
*Schizooura*	?	?	?	?	?	?	?	1	1	?	1	0	?	0	0	0	1	0/1	2	?	0	1	1	0	0	0	0	?	0	0	?	1
*Jianchangornis*	0	?	1	1	0	?	?	1	1	2	1	0	0	0	?	?	?	?	?	?	0	1	0	0	?	0	0	0	?	1	?	?
*Archaeorhynchus*	0	?	?	?	?	?	2	1	1	1	1	0	?	0	?	?	?	?	0	?	0	1	0	0	?	0	?	0	?	?	?	?
*Longicrusavis*	0/1	1	2	0	0	?	1	1	1	2	1	1	1	1	?	?	1	0	2	?	0	1	0	0	?	0	0	0	0	1	?	?
*Apsaravis*	0	1	2	1	1	?	?	1	1	2	1	1	1	1	1	?	1	?	2	0	0	2	1	0	?	0	?	0	?	?	?	?
*Hongshanornis*	1	1	2	0	1	?	?	1	1	2	1	1	1	1	?	0	1	0	1/2	?	0	2	0	0	?	0	0	0	0	1	1	1
*Yanornis*	0	1	?	0	0	?	?	1	1	1	1	0	?	0	0/1	1	?	1	2	?	0	1	0	0	?	0	0	0	0	1	?	?
*Patagopteryx*	0	?	0	0	1	1	1	1	1	2	1	0	1	0	1	1	1	0	0	0	0	1/2	0	0	0	0	1	0	0	1	?	?
*Yixianornis*	?	?	?	?	?	?	?	1	1	1/2	1	?	?	0	1	1	1	0	?	?	0	1	0/1	?	?	0	0	0	0	1	?	1
*Gansus yumenensis*	1	1	1/2	0	0	?	2	1	1	2	1	1	1	1	1	0	1	0	1/2	1	0	2	1	0	0	0	0	0	0	1	?	?
*Gansus zheni*	1	1	1	0	0	1	?	1	1	2	?	1	1	1	?	?	1	0	2	?	0	2	1	0	?	0	0	?	0	1	?	?
*Ichthyornis*	1	1	1	0	0	?	?	?	1	2	1	1	2	2	1	?	1	?	2	1	0	2	0	0	0	0	?	?	?	?	?	?
*Hesperornis*	1	1	1	0	1	1	2	1	1	2	1	1	2	2	1	0	1	3	2	2	3	2	2	0	0	0	?	1	?	?	?	?
*Parahesperornis*	1	1	1	0	1	1	2	1	1	2	1	1	2	1	1	0	1	?	2	2	3	2	2	0	0	0	1	1	?	?	?	?
*Vegavis*	2	?	1	?	?	?	?	1	1	2	1	1	?	?	2	?	1	?	?	?	?	1	?	?	?	0	?	?	?	?	?	?
*Anas*	2	0	1	0	1	1	2	1	1	2	1	1	2	1	2	0	1	3	2	1	0	2	2	0	0	0	0	0	0	0	1	1
*Gallus*	2	0	1	0	1	1	2	1	1	2	1	1	2	1	2	0	1	3	2	2	0	1	2	0	1	0	0	0	0	0	1	1
*Bellulornis*	0	?	?	?	?	?	?	1	1	2	1	0	1	0	?	?	?	0	2	?	0	2	0	0	?	0	0	?	0	0	?	1
*Fortunguavis*	0	1	?	?	?	1	?	1	1	0	1	0	0	0	0	0	0	?	0	?	0	2	0	1	0	0	1	0	0	1	?	?
*Qiliania*	0	0	0	1	0	?	?	1	1	0	?	0	0	0	0	?	?	1	1	?	0/1	?	2	?	?	0	2	0	0	0/1	?	?
*shenqiornis*	?	?	?	?	?	?	?	?	?	0/1	?	0	?	0	?	?	?	2	?	?	0/1	?	0	?	?	?	?	?	0	?	?	?
*Sulcavis*	0	?	0	1	1	?	?	1	1	0	1	0	0	0	?	?	0	1/2/3	?	?	0	1	0	1	?	0	1	0	0	1	?	?
*Bohaiornis*	0	?	0	0	1	0	?	1	1	0/1	1	0	0	0	?	?	0	1	?	0/1	0	1	0	1	?	0	1	?	0	1	?	?
*Parabohaiornis*	0	0	0	1	0	0	?	1	?	0	1	0	0	0	?	?	0	1	2	?	0	1	0	1	?	0	2	?	0	1	?	?
*Longusunguis*	0	0	0	1	1	0	?	1	?	0	1	?	?	0	0	?	?	1	?	?	0	1/2	0	1	0	0	2	?	0	?	?	?
*Zhouornis*	0	?	?	1	1	?	?	1	1	1	1	0	0	0	?	?	0	1	?	?	0	1	0	1	?	0	1	?	0	1	?	?
CUGB P1202	?	?	?	?	?	?	?	?	?	0/1	?	0	?	0	?	?	?	1	?	?	0	0	0	1	?	0	?	?	?	?	1	0
*Gretcheniao*	?	0	0	1	1	1	?	1	1	1	1	0	0	0	?	?	0	1	1	?	0	1	0/1	0	?	0	1/2	?	0	1	?	?

## Supplemental Information

10.7717/peerj.7846/supp-1 Supplemental Information 1 *NeXus* file of the character matrix used in the phylogenetic analyses (see Appendix 1 for the list of characters).

10.7717/peerj.7846/supp-2 Supplemental Information 2 Dataset of extant birds used in this study. Specimens were measured directly from the Burke Museum of the University of Washington, Seattle (UWBM), and the Beaty Biodiversity Museum of the University of British Columbia, Vancouver (UBCBBM). Other specimens were taken from references: (BR) Bruderer et al. (2010); (FS) Flight software’s dataset from Pennycuick (2008); (VF) Viscor & Fuster, 1987.

10.7717/peerj.7846/supp-3 Supplemental Information 3 Comparative illustrations of the forelimb bones in different “bohaiornithids”. Blue and yellow areas show deviation in the proportions of the carpometacarpus (red = longer; yellow = shorter) with respect to that of Bohaiornis guoi. Note the proportionally much longer carpometacarpus of Gretcheniao sinensis.

## Institutional Abbreviations

BMNH: Beijing Museum of Natural History (Beijing, China)
CNUVB: Capital Normal University (Beijing, China)
DNHM: Dalian Natural History Museum (Dalian, China)
CUGB: China University of Geosciences, Beijing
IVPP: Institute of Vertebrate Paleontology and Paleoanthropology (Beijing, China)
LACM: Natural History Museum of Los Angeles County (Los Angeles, USA)
LPM: Liaoning Paleontology Museum, Shenyang Normal University (Shenyang, China).

## Additional Information and Declarations

### New Species Registration

The following information was supplied regarding the registration of a newly described species: Publication LSID: urn:lsid:zoobank.org:pub:BC33BE8C-7AFA-4D44-8E71-7EC61504AF07. *Gretcheniao sinensis* LSID: urn:lsid:zoobank.org:act:3EE2342A-D3FC-413B-ACE9-583B66796A1C.

### Data Availability

The following information was supplied regarding data availability: The specimen described in this study is housed at Beijing Museum of Natural History (Beijing, China) under the catalog number BMNH Ph 829. The specimen was prepared by personnel of the Natural History Museum of Los Angeles County. A resin cast of the specimen is deposited in the collection of the Dinosaur Institute of the Natural History Museum of Los Angeles County under the number LACM 7917/156630.
