# Supplementary material for: New Bohaiornis-like bird from the Early Cretaceous of China: enantiornithine interrelationships and flight performance
Source: PeerJ. 2019 Oct 25;7:e7846. doi: 10.7717/peerj.7846 (PMC6816414; doi:10.7717/peerj.7846)
Supplement: Supplemental Information 2 — Specimens were measured directly from the Burke Museum of the University of Washington, Seattle (UWBM), and the Beaty Biodiversity Museum of the University of British Columbia, Vancouver (UBCBBM). Other specimens were taken from references: (BR) Bruderer et al. (2010); (FS) Flight software’s dataset from Pennycuick (2008); (VF) Viscor & Fuster, 1987. [file peerj-07-7846-s002.docx]

**Supplementary Table 1**

Dataset of extant birds used in this study. Specimens were measured directly from the Burke Museum of the University of Washington, Seattle (UWBM), and the Beaty Biodiversity Museum of the University of British Columbia, Vancouver (UBCBBM). Other specimens were taken from references:

(BR) Bruderer, Peter, Boldt and Liechti 2010. Wing-beat characteristics of birds recorded with tracking radar and cine camera. *Ibis* 152, 272–291.

(FS) Flight software's dataset: in Pennycuick 2008. Modelling the flying bird. Academic Press, 480 pp.

(VF) Viscor and Fuster 1987. Relationships between morphological parameters in birds with different flying habits. *Comparative Biochemistry and Physiology. Part A, Physiology* 87, 231–249.

| **Specie** | **Specimen/Source** | **Flight strategy** | ***M_b_* (Kg)** | ***B* (m)** | ***S_L_* (m^2^)** | ***AR*** | ***WL* (Kg/m2)** |
| --- | --- | --- | --- | --- | --- | --- | --- |
| Accipiter brevipes | BR | Flap-gliding | ,2200 | ,7000 | ,0739 | 6,6300 | 2,9770 |
| Accipiter gentilis | UWBM 64855 | Flap-gliding | 1,1000 | 1,1250 | ,2064 | 6,1300 | 5,3295 |
| Accipiter gentilis | UWBM 80189 | Flap-gliding | ,7680 | 1,0600 | ,1801 | 6,2400 | 4,2643 |
| Accipiter gentilis F | BR | Flap-gliding | 1,2000 | 1,2000 | ,2400 | 6,0000 | 5,0000 |
| Accipiter gentilis M | BR | Flap-gliding | ,7170 | 1,0300 | ,1643 | 6,4600 | 4,3640 |
| Accipiter nisus F | BR | Flap-gliding | ,2950 | ,7600 | ,0912 | 6,3300 | 3,2346 |
| Accipiter nisus M | BR | Flap-gliding | ,1150 | ,6200 | ,0638 | 6,0300 | 1,8025 |
| Accipiter virgatus | UWBM 59708 | Flap-gliding | ,1200 | ,5400 | ,0475 | 6,1400 | 2,5263 |
| Accipiter virgatus | UWBM 59781 | Flap-gliding | ,1060 | ,5500 | ,0492 | 6,1500 | 2,1545 |
| Accipiter virgatus | UWBM 59884 | Flap-gliding | ,1070 | ,5300 | ,0471 | 5,9700 | 2,2718 |
| Accipiter virgatus | UWBM 59915 | Flap-gliding | ,0961 | ,5350 | ,0485 | 5,9000 | 1,9814 |
| Acrocephalus arundin | UWBM 56436 | Bounding | ,0345 | ,2960 | ,0166 | 5,2800 | 2,0783 |
| Acrocephalus arundin | UWBM 56679 | Bounding | ,0347 | ,2910 | ,0156 | 5,4500 | 2,2244 |
| Acrocephalus arundin | UWBM 56685 | Bounding | ,0332 | ,3100 | ,0167 | 5,7700 | 1,9880 |
| Acrocephalus arundin | BR | Bounding | ,0310 | ,2700 | ,0117 | 6,2300 | 2,6496 |
| Acrocephalus palustr | BR | Bounding | ,0130 | ,2000 | ,0072 | 5,5600 | 1,8056 |
| Acrocephalus scirpac | BR | Bounding | ,0100 | ,2000 | ,0074 | 5,4100 | 1,3514 |
| Aegotheles cristatus | UWBM 62800 | Continuous flapping | ,0465 | ,4100 | ,0329 | 5,1100 | 1,4134 |
| Aegotheles cristatus | UWBM 62801 | Continuous flapping | ,0349 | ,3880 | ,0287 | 5,2500 | 1,2160 |
| Alauda arvensis | UWBM 47527 | Bounding | ,0345 | ,3200 | ,0197 | 5,2000 | 1,7513 |
| Alauda arvensis | UWBM 47606 | Bounding | ,0408 | ,3300 | ,0185 | 5,8900 | 2,2054 |
| Alauda arvensis | BR | Bounding | ,0310 | ,3600 | ,0233 | 5,5600 | 1,3305 |
| Alca torda | VF | Brief continuous flapping | ,7800 | ,6810 | ,0382 | 12,1400 | 20,4188 |
| Alcedo atthis | UWBM 63315 | Continuous flapping | ,0350 | ,2800 | ,0130 | 6,0200 | 2,6923 |
| Alcedo atthis | UWBM 66027 | Continuous flapping | ,0405 | ,2780 | ,0128 | 6,0300 | 3,1641 |
| Alcedo atthis | UWBM 71986 | Continuous flapping | ,0320 | ,2670 | ,0117 | 6,1000 | 2,7350 |
| Alle alle | VF | Brief continuous flapping | ,0912 | ,3870 | ,0167 | 8,9700 | 5,4611 |
| Anas acuta | BR | Continuous flapping | ,9110 | ,9300 | ,0918 | 9,4200 | 9,9237 |
| Anas clypeata | BR | Continuous flapping | ,5980 | ,7800 | ,0750 | 8,1100 | 7,9733 |
| Anas platyrhynchos | UWBM 56923 | Continuous flapping | 1,0360 | ,9320 | ,1080 | 8,0400 | 9,5926 |
| Anas platyrhynchos | BR | Continuous flapping | 1,0940 | ,8900 | ,1054 | 7,5200 | 10,3795 |
| Anas strepera | BR | Continuous flapping | ,7400 | ,9000 | ,1000 | 8,1000 | 7,4000 |
| Anthus pratensis | BR | Bounding | ,0200 | ,2700 | ,0143 | 5,1000 | 1,3986 |
| Anthus spinoletta | BR | Bounding | ,0260 | ,2800 | ,0151 | 5,1900 | 1,7219 |
| Anthus trivialis | BR | Bounding | ,0220 | ,2800 | ,0126 | 6,2200 | 1,7460 |
| Apus affinis | BR | Flap-gliding | ,0180 | ,3300 | ,0100 | 10,8900 | 1,8000 |
| Apus apus | BR | Flap-gliding | ,0400 | ,4000 | ,0150 | 10,6700 | 2,6667 |
| Apus melba | BR | Flap-gliding | ,0810 | ,5000 | ,0271 | 9,2300 | 2,9889 |
| Apus pallidus | BR | Flap-gliding | ,0400 | ,4000 | ,0150 | 10,6700 | 2,6667 |
| Aquila pomarina | BR | Flap-gliding | 2,0150 | 1,8000 | ,5133 | 6,3100 | 3,9256 |
| Ardea cinerea | BR | Continuous flapping | 1,2100 | 1,6000 | ,3580 | 7,1500 | 3,3799 |
| Ardea purpurea | BR | Continuous flapping | 1,1090 | 1,3700 | ,2488 | 7,5400 | 4,4574 |
| Artamus minor | UWBM 57477 | Flap-gliding | ,0150 | ,3010 | ,0133 | 6,8100 | 1,1278 |
| Artamus minor | UWBM 57539 | Flap-gliding | ,0140 | ,2950 | ,0127 | 6,8300 | 1,1024 |
| Artamus personatus | UWBM 57691 | Flap-gliding | ,0394 | ,3480 | ,0178 | 6,8200 | 2,2135 |
| Artamus superciliosu | UWBM 57688 | Flap-gliding | ,0385 | ,3650 | ,0210 | 6,3600 | 1,8333 |
| Asio flammeus | BR | Flap-gliding | ,3500 | 1,0300 | ,1343 | 7,9000 | 2,6061 |
| Asio otus | UWBM 59694 | Flap-gliding | ,3400 | ,9750 | ,1458 | 6,5200 | 2,3320 |
| Asio otus | UWBM 85097 | Flap-gliding | ,2770 | ,9550 | ,1370 | 6,6600 | 2,0219 |
| Asio otus | BR | Flap-gliding | ,2800 | ,9400 | ,1300 | 6,8000 | 2,1538 |
| Aythia affinis | VF | Brief continuous flapping | ,7630 | ,7400 | ,0472 | 11,6000 | 16,1653 |
| Aythia collaris | VF | Brief continuous flapping | ,7573 | ,7100 | ,0460 | 10,9600 | 16,4630 |
| Aythia ferina | VF | Brief continuous flapping | ,8420 | ,7740 | ,0615 | 9,7400 | 13,6911 |
| Aythia fuligula | VF | Brief continuous flapping | ,7410 | ,7060 | ,0474 | 10,5200 | 15,6329 |
| Aythia marila | VF | Brief continuous flapping | ,6750 | ,8140 | ,0621 | 10,6700 | 10,8696 |
| Aythya ferina | BR | Brief continuous flapping | 1,0050 | ,7800 | ,0660 | 9,2200 | 15,2273 |
| Aythya fuligula | BR | Brief continuous flapping | ,8060 | ,7500 | ,0630 | 8,9300 | 12,7937 |
| Brachyramphus marmor | UWBM 79447 | Brief continuous flapping | ,2580 | ,4390 | ,0202 | 9,5500 | 12,7723 |
| Bubo virginianus | UWBM 72511 | Flap-gliding | ,8320 | 1,3420 | ,2903 | 6,2000 | 2,8660 |
| Buteo buteo buteo | BR | Flap-gliding | ,9640 | 1,2900 | ,2540 | 6,5500 | 3,7953 |
| Buteo buteo vulpinus | BR | Flap-gliding | ,5800 | 1,1900 | ,2070 | 6,8400 | 2,8019 |
| Buteo jamaicensis | UWBM 69545 | Flap-gliding | 1,1400 | 1,0260 | ,2064 | 5,1000 | 5,5233 |
| Buteo jamaicensis | UWBM 69579 | Flap-gliding | 1,3040 | 1,3420 | ,2995 | 6,0100 | 4,3539 |
| Cacatua alba | UWBM 82950 | Continuous flapping | ,6400 | 1,0100 | ,2075 | 4,9200 | 3,0843 |
| Calidris alba | BR | Continuous flapping | ,0500 | ,3500 | ,0160 | 7,6600 | 3,1250 |
| Calidris alpina | BR | Continuous flapping | ,0440 | ,3600 | ,0160 | 8,1000 | 2,7500 |
| Caprimulgus caroline | UWBM 84731 | Flap-gliding | ,1130 | ,6500 | ,0539 | 7,8400 | 2,0965 |
| Caprimulgus caroline | UWBM 87389 | Flap-gliding | ,0986 | ,6100 | ,0533 | 6,9900 | 1,8499 |
| Caprimulgus ruficoll | BR | Flap-gliding | ,0690 | ,6700 | ,0567 | 7,9200 | 1,2169 |
| Carduelis cannabina | BR | Bounding | ,0180 | ,2400 | ,0093 | 6,1900 | 1,9355 |
| Carduelis carduelis | BR | Bounding | ,0180 | ,2400 | ,0099 | 5,8200 | 1,8182 |
| Carduelis chloris | BR | Bounding | ,0290 | ,2700 | ,0112 | 6,5100 | 2,5893 |
| Carduelis spinus | BR | Bounding | ,0110 | ,2100 | ,0079 | 5,5800 | 1,3924 |
| Cathartes aura | UWBM 81580 | Flap-gliding | 1,7750 | 1,7100 | ,4388 | 6,6600 | 4,0451 |
| Cathartes aura | UWBM 84397 | Flap-gliding | 1,6600 | 1,6800 | ,4206 | 6,7100 | 3,9467 |
| Centropus milo | UWBM 66047 | Continuous flapping | ,7900 | ,7700 | ,1711 | 3,4600 | 4,6172 |
| Chalcopsitta cardina | UWBM 58701 | Continuous flapping | ,2350 | ,5550 | ,0510 | 6,0400 | 4,6078 |
| Chalcopsitta cardina | UWBM 63146 | Continuous flapping | ,2080 | ,5400 | ,0471 | 6,1900 | 4,4161 |
| Chalcopsitta cardina | UWBM 63301 | Continuous flapping | ,1858 | ,5460 | ,0452 | 6,5900 | 4,1106 |
| Charadrius dubius | UWBM 56903 | Continuous flapping | ,0345 | ,3580 | ,0156 | 8,2200 | 2,2115 |
| Charadrius dubius | UWBM 56904 | Continuous flapping | ,0360 | ,3600 | ,0149 | 8,7000 | 2,4161 |
| Charadrius hiaticula | BR | Continuous flapping | ,0530 | ,4000 | ,0179 | 8,9400 | 2,9609 |
| Chauna torquata | UBCBBM 016558 | Flap-gliding | 2,2000 | 1,4900 | ,4357 | 5,1000 | 5,0500 |
| Chlidonias niger | UWBM 56898 | Flap-gliding | ,0677 | ,6230 | ,0410 | 9,4600 | 1,6512 |
| Chlidonias niger | UWBM 56906 | Flap-gliding | ,0620 | ,6170 | ,0382 | 9,9700 | 1,6230 |
| Ciconia ciconia | BR | Flap-gliding | 3,6000 | 2,1600 | ,6508 | 7,1700 | 5,5317 |
| Ciconia nigra | BR | Flap-gliding | 3,0000 | 1,8500 | ,5000 | 6,8500 | 6,0000 |
| Circus aeruginosus | UWBM 56450 | Flap-gliding | ,5400 | 1,1950 | ,2010 | 7,1000 | 2,6866 |
| Circus aeruginosus | UWBM 56456 | Flap-gliding | ,5600 | 1,0540 | ,1642 | 6,7600 | 3,4105 |
| Circus aeruginosus | UWBM 56488 | Flap-gliding | ,5450 | 1,2320 | ,1933 | 7,8500 | 2,8195 |
| Circus aeruginosus | BR | Flap-gliding | ,6510 | 1,3300 | ,2248 | 7,8700 | 2,8959 |
| Circus cyaneus | BR | Flap-gliding | ,4300 | 1,1000 | ,1539 | 7,8600 | 2,7940 |
| Circus macrourus | BR | Flap-gliding | ,3580 | 1,0600 | ,1553 | 7,2400 | 2,3052 |
| Circus pygargus | BR | Flap-gliding | ,2500 | 1,0400 | ,1290 | 8,3800 | 1,9380 |
| Coccothraustes cocco | UWBM 57930 | Bounding | ,0511 | ,3280 | ,0195 | 5,5300 | 2,6205 |
| Coccothraustes cocco | UWBM 57943 | Bounding | ,0506 | ,3220 | ,0180 | 5,7500 | 2,8111 |
| Columba livia | UWBM 66264 | Continuous flapping | ,2450 | ,6380 | ,0718 | 5,6700 | 3,4123 |
| Columba livia | BR | Continuous flapping | ,3500 | ,6700 | ,0649 | 6,9200 | 5,3929 |
| Columba palumbus | BR | Continuous flapping | ,5000 | ,7500 | ,0904 | 6,2200 | 5,5310 |
| Columba rupestris | UWBM 59754 | Continuous flapping | ,2980 | ,6650 | ,0612 | 7,2300 | 4,8693 |
| Columba rupestris | UWBM 59755 | Continuous flapping | ,2340 | ,6650 | ,0686 | 6,4400 | 3,4111 |
| Columba rupestris | UWBM 59986 | Continuous flapping | ,2400 | ,6510 | ,0629 | 6,7300 | 3,8156 |
| Coracias garrulus | UWBM 56480 | Continuous flapping | ,1690 | ,6460 | ,0671 | 6,2200 | 2,5186 |
| Coracias garrulus | UWBM 56485 | Continuous flapping | ,1620 | ,6370 | ,0648 | 6,2600 | 2,5000 |
| Coracias garrulus | UWBM 56647 | Continuous flapping | ,1680 | ,6870 | ,0714 | 6,6100 | 2,3529 |
| Coracias garrulus | UWBM 56886 | Continuous flapping | ,1460 | ,6700 | ,0711 | 6,3100 | 2,0534 |
| Corvus corax | UWBM 59566 | Flap-gliding | 1,4300 | 1,3350 | ,2781 | 6,4100 | 5,1420 |
| Corvus corax | UWBM 61493 | Flap-gliding | 1,3000 | 1,3820 | ,2592 | 7,3700 | 5,0154 |
| Corvus corax | UWBM 64779 | Flap-gliding | 1,0500 | 1,2950 | ,2449 | 6,8500 | 4,2875 |
| Corvus corax | BR | Flap-gliding | ,9450 | 1,1400 | ,2472 | 5,2600 | 3,8228 |
| Corvus corone | UWBM 56471 | Continuous flapping | ,5750 | ,9660 | ,1603 | 5,8200 | 3,5870 |
| Corvus corone | UWBM 56495 | Flap-gliding | ,5200 | 1,0010 | ,1665 | 6,0200 | 3,1231 |
| Corvus corone | BR | Flap-gliding | ,5520 | ,9300 | ,1470 | 5,8800 | 3,7551 |
| Corvus frugilegus | BR | Continuous flapping | ,4540 | ,9300 | ,1373 | 6,3000 | 3,3066 |
| Corvus monedula | BR | Continuous flapping | ,1810 | ,6000 | ,0618 | 5,8300 | 2,9288 |
| Corvus ruficollis | BR | Continuous flapping | ,7200 | 1,1000 | ,1944 | 6,2200 | 3,7037 |
| Cotournix cotournix | UWBM 61354 | Brief continuous flapping | ,1010 | ,3470 | ,0193 | 6,2400 | 5,2332 |
| Cotournix cotournix | UWBM 64806 | Brief continuous flapping | ,0955 | ,3630 | ,0190 | 6,9200 | 5,0263 |
| Coturnix coturnix | BR | Brief continuous flapping | ,0960 | ,3700 | ,0200 | 6,8500 | 4,8000 |
| Cuculus canorus | UWBM 56781 | Continuous flapping | ,1180 | ,6090 | ,0563 | 6,5800 | 2,0959 |
| Cuculus canorus | UWBM 58077 | Continuous flapping | ,1134 | ,6020 | ,0585 | 6,1900 | 1,9385 |
| Cuculus canorus | UWBM 59705 | Continuous flapping | ,1440 | ,6450 | ,0646 | 6,4400 | 2,2291 |
| Cursorius cursor | BR | Continuous flapping | ,1150 | ,5400 | ,0407 | 7,1600 | 2,8256 |
| Cygnus olor | UWBM 56584 | Continuous flapping | 8,2100 | 2,1500 | ,5938 | 7,7800 | 13,8262 |
| Cygnus olor | UWBM 56585 | Continuous flapping | 9,4400 | 2,2700 | ,6550 | 7,8700 | 14,4122 |
| Cygnus olor | BR | Continuous flapping | 8,7600 | 2,3000 | ,6504 | 8,1300 | 13,4686 |
| Dacelo novaeguineae | UWBM 57788 | Continuous flapping | ,2500 | ,7700 | ,1022 | 5,8000 | 2,4462 |
| Dacelo novaeguineae | UWBM 62803 | Continuous flapping | ,3220 | ,7400 | ,0954 | 5,7400 | 3,3753 |
| Dacelo novaeguineae | UWBM 62895 | Continuous flapping | ,3120 | ,7340 | ,0926 | 5,8200 | 3,3693 |
| Delichon urbica | BR | Flap-gliding | ,0180 | ,2800 | ,0107 | 7,3300 | 1,6822 |
| Diomedea exulans | Flight 1.24 | Flap-gliding | 9,5700 | 3,0600 | ,6440 | 14,5000 | 14,8602 |
| Diomedea exulans | VF | Flap-gliding | 8,5020 | 3,4080 | ,6206 | 18,7100 | 13,6996 |
| Dryocopus martius | UWBM 56955 | Continuous flapping | ,3400 | ,7400 | ,1085 | 5,0500 | 3,1336 |
| Dryocopus martius | UWBM 59748 | Continuous flapping | ,3500 | ,7350 | ,1130 | 4,7800 | 3,0973 |
| Dryocopus martius | UWBM 59988 | Continuous flapping | ,3480 | ,7530 | ,1142 | 4,9600 | 3,0473 |
| Dryocopus martius | UWBM 64701 | Continuous flapping | ,3160 | ,6950 | ,0968 | 4,9900 | 3,2645 |
| Ducula rubricera | UWBM 58730 | Continuous flapping | ,6600 | ,7550 | ,0990 | 5,7600 | 6,6667 |
| Ducula rubricera | UWBM 63171 | Continuous flapping | ,6500 | ,7540 | ,1054 | 5,3900 | 6,1670 |
| Egretta garzetta | BR | Continuous flapping | ,5000 | ,9200 | ,1150 | 7,3600 | 4,3478 |
| Emberiza hortulana | BR | Bounding | ,0220 | ,2600 | ,0138 | 4,9000 | 1,5942 |
| Emberiza schoeniclus | UWBM 56856 | Bounding | ,0270 | ,2590 | ,0148 | 4,5300 | 1,8243 |
| Eolophus roseicapill | UWBM 60857 | Continuous flapping | ,3760 | ,7900 | ,0957 | 6,5200 | 3,9289 |
| Erithacus rubecula | BR | Bounding | ,0150 | ,2200 | ,0101 | 4,7900 | 1,4851 |
| Eudynamus scolopacea | UWBM 62904 | Continuous flapping | ,2410 | ,6300 | ,0670 | 5,9200 | 3,5970 |
| Eudynamus scolopacea | UWBM 64911 | Continuous flapping | ,2365 | ,6080 | ,0689 | 5,3700 | 3,4325 |
| Eudynamus scolopacea | UWBM 64912 | Continuous flapping | ,1562 | ,6200 | ,0679 | 5,6600 | 2,3004 |
| Falco biarmicus | BR | Flap-gliding | ,5950 | 1,0560 | ,1410 | 7,9100 | 4,2199 |
| Falco columbarius M | BR | Flap-gliding | ,1590 | ,5900 | ,0493 | 7,0600 | 3,2252 |
| Falco concolor | BR | Flap-gliding | ,2500 | ,9000 | ,1196 | 6,7700 | 2,0903 |
| Falco eleonorae | BR | Flap-gliding | ,3600 | 1,0000 | ,1500 | 6,6700 | 2,4000 |
| Falco naumanni | BR | Flap-gliding | ,1480 | ,6500 | ,0611 | 6,9100 | 2,4223 |
| Falco pelegrinoides | BR | Flap-gliding | ,4110 | ,7700 | ,0723 | 8,2000 | 5,6846 |
| Falco peregrinus | UWBM 78842 | Flap-gliding | ,6990 | 1,0000 | ,1248 | 8,0200 | 5,6010 |
| Falco peregrinus | UWBM 79327 | Flap-gliding | 1,0720 | 1,2630 | ,1720 | 9,2700 | 6,2326 |
| Falco peregrinus F | BR | Flap-gliding | ,9980 | 1,1000 | ,1478 | 8,1900 | 6,7524 |
| Falco peregrinus M | BR | Flap-gliding | ,5700 | ,9600 | ,1098 | 8,3900 | 5,1913 |
| Falco subbuteo | BR | Flap-gliding | ,1840 | ,7500 | ,0653 | 8,6100 | 2,8178 |
| Falco tinnunculus | BR | Flap-gliding | ,2060 | ,7500 | ,0777 | 7,2400 | 2,6512 |
| Falco vespertinus | BR | Flap-gliding | ,1650 | ,7200 | ,0728 | 7,1200 | 2,2665 |
| Ficedula hypoleuca | BR | Bounding | ,0130 | ,2400 | ,0091 | 6,3300 | 1,4286 |
| Fratercula arctica | VF | Brief continuous flapping | ,2720 | ,5640 | ,0345 | 9,2200 | 7,8841 |
| Fregata magnificens | Flight 1.24 | Flap-gliding | 1,6700 | 2,1400 | ,3720 | 12,3000 | 4,4892 |
| Fringilla coelebs | BR | Bounding | ,0230 | ,2600 | ,0130 | 5,2000 | 1,7692 |
| Fringilla montifring | BR | Bounding | ,0230 | ,2700 | ,0125 | 5,8300 | 1,8400 |
| Fulica atra | UWBM 56581 | Brief continuous flapping | ,8250 | ,7360 | ,0752 | 7,2000 | 10,9707 |
| Fulica atra | UWBM 56917 | Brief continuous flapping | ,5630 | ,6450 | ,0611 | 6,8000 | 9,2144 |
| Fulica atra | BR | Brief continuous flapping | ,7440 | ,7500 | ,0700 | 8,0400 | 10,6286 |
| Fulmarus glacialis | VF | Flap-gliding | ,7245 | 1,0900 | ,1020 | 11,6500 | 7,1029 |
| Galerida cristata | BR | Bounding | ,0450 | ,3400 | ,0200 | 5,7800 | 2,2500 |
| Gallicrex cinerea | UWBM 64910 | Brief continuous flapping | ,3305 | ,6550 | ,0758 | 5,6600 | 4,3602 |
| Gallicrex cinerea | UWBM 64933 | Brief continuous flapping | ,1940 | ,5650 | ,0494 | 6,4600 | 3,9271 |
| Gallinago gallinago | BR | Continuous flapping | ,1220 | ,4600 | ,0309 | 6,8500 | 3,9482 |
| Garrulus glandarius | BR | Continuous flapping | ,1500 | ,5500 | ,0662 | 4,5700 | 2,2659 |
| Gavia arctica | VF | Brief continuous flapping | 1,4950 | 1,2000 | ,1196 | 12,0400 | 12,5000 |
| Gavia immer | VF | Brief continuous flapping | 2,4250 | 1,4700 | ,1358 | 15,9100 | 17,8571 |
| Gavia stellata | VF | Brief continuous flapping | ,9570 | 1,0400 | ,0890 | 12,1500 | 10,7528 |
| Glareola nordmanni | BR | Continuous flapping | ,1000 | ,6400 | ,0540 | 7,5900 | 1,8519 |
| Glareola pratincola | BR | Continuous flapping | ,0800 | ,6300 | ,0503 | 7,8900 | 1,5905 |
| Grus canadensis | UWBM 85110 | Continuous flapping | 3,5000 | 1,6900 | ,4199 | 6,8000 | 8,3353 |
| Grus canadensis | UWBM 85111 | Continuous flapping | 4,2000 | 1,8200 | ,4882 | 6,7900 | 8,6030 |
| Grus canadensis | UWBM 85112 | Continuous flapping | 3,9800 | 1,8000 | ,4401 | 7,3600 | 9,0434 |
| Haematopus ostralegu | BR | Continuous flapping | ,4030 | ,8500 | ,0891 | 8,1100 | 4,5230 |
| Haliaeetus vocifer | BR | Flap-gliding | 3,0000 | 1,9000 | ,5000 | 7,2200 | 6,0000 |
| Haliastur indus | UWBM 58751 | Flap-gliding | ,6050 | 1,1790 | ,2144 | 6,4800 | 2,8218 |
| Haliastur indus | UWBM 73850 | Flap-gliding | ,6120 | 1,3150 | ,2585 | 6,6900 | 2,3675 |
| Haliastur indus | UWBM 76196 | Flap-gliding | ,5180 | 1,1750 | ,2133 | 6,4700 | 2,4285 |
| Hemiprocne mystacea | UWBM 60348 | Flap-gliding | ,0575 | ,5130 | ,0278 | 9,4600 | 2,0683 |
| Hemiprocne mystacea | UWBM 63244 | Flap-gliding | ,0648 | ,5280 | ,0279 | 9,9800 | 2,3226 |
| Hieraaetus fasciatus | BR | Flap-gliding | 2,0490 | 1,7400 | ,3792 | 7,9800 | 5,4035 |
| Hieraaetus pennatus | BR | Flap-gliding | ,5950 | 1,1600 | ,2004 | 6,7100 | 2,9691 |
| Himantopus himantopu | UWBM 56452 | Continuous flapping | ,1850 | ,7100 | ,0567 | 8,8900 | 3,2628 |
| Himantopus himantopu | UWBM 56863 | Continuous flapping | ,1980 | ,7350 | ,0588 | 9,1900 | 3,3673 |
| Himantopus himantopu | UWBM 60150 | Continuous flapping | ,1500 | ,6730 | ,0544 | 8,3300 | 2,7574 |
| Himantopus himantopu | BR | Continuous flapping | ,1600 | ,7500 | ,0600 | 9,3800 | 2,6667 |
| Hippolais icterina | BR | Bounding | ,0140 | ,2200 | ,0081 | 5,9800 | 1,7284 |
| Hippolais pallida | BR | Bounding | ,0120 | ,2000 | ,0083 | 4,8200 | 1,4458 |
| Hippolais polyglotta | BR | Bounding | ,0110 | ,2000 | ,0083 | 4,8200 | 1,3253 |
| Hirundo daurica | BR | Flap-gliding | ,0220 | ,3300 | ,0154 | 7,0700 | 1,4286 |
| Hirundo rustica | BR | Flap-gliding | ,0170 | ,3200 | ,0140 | 7,3100 | 1,2143 |
| Hoplopterus spinosus | BR | Continuous flapping | ,1500 | ,7000 | ,0700 | 7,0000 | 2,1429 |
| Hydrobates pelagicus | VF | Flap-gliding | ,0174 | ,3320 | ,0100 | 11,0200 | 1,7400 |
| Ixobrychus cinnamomo | UWBM 64913 | Continuous flapping | ,1218 | ,5320 | ,0446 | 6,3500 | 2,7309 |
| Jynx torquilla | BR | Bounding | ,0300 | ,2900 | ,0150 | 5,6100 | 2,0000 |
| Lagopus lagopus | UWBM 58894 | Brief continuous flapping | ,6180 | ,6900 | ,0730 | 6,5200 | 8,4658 |
| Lagopus lagopus | UWBM 58896 | Brief continuous flapping | ,5750 | ,6280 | ,0625 | 6,3100 | 9,2000 |
| Lagopus lagopus | UWBM 79851 | Brief continuous flapping | ,5875 | ,6690 | ,0748 | 5,9800 | 7,8543 |
| Lanius collurio | BR | Bounding | ,0300 | ,3000 | ,0145 | 6,2100 | 2,0690 |
| Lanius minor | UWBM 56649 | Bounding | ,0432 | ,3500 | ,0215 | 5,7000 | 2,0093 |
| Larus argentatus | UWBM 56577 | Flap-gliding | 1,1750 | 1,5850 | ,2794 | 8,9900 | 4,2054 |
| Larus argentatus | UWBM 56578 | Flap-gliding | 1,2500 | 1,5400 | ,2512 | 9,4400 | 4,9761 |
| Larus argentatus | UWBM 56728 | Flap-gliding | 1,0650 | 1,4740 | ,2212 | 9,8200 | 4,8146 |
| Larus argentatus | UWBM 56821 | Flap-gliding | 1,1600 | 1,5060 | ,2350 | 9,6500 | 4,9362 |
| Larus argentatus | BR | Flap-gliding | ,7050 | 1,3500 | ,2001 | 9,1100 | 3,5232 |
| Larus cachinnans | BR | Flap-gliding | 1,0000 | 1,4300 | ,2496 | 8,1900 | 4,0064 |
| Larus canus | BR | Flap-gliding | ,2760 | 1,1000 | ,1380 | 8,7700 | 2,0000 |
| Larus fuscus | BR | Flap-gliding | ,7970 | 1,3400 | ,1895 | 9,4800 | 4,2058 |
| Larus pipixcan | UWBM 86565 | Flap-gliding | ,2810 | ,9200 | ,0998 | 8,4900 | 2,8156 |
| Larus ridibundus | BR | Flap-gliding | ,2750 | ,9600 | ,0983 | 9,3800 | 2,7976 |
| Limosa lapponica | BR | Continuous flapping | ,2710 | ,7200 | ,0550 | 9,4300 | 4,9273 |
| Locustella naevia | BR | Bounding | ,0130 | ,1900 | ,0070 | 5,1600 | 1,8571 |
| Lullula arborea | BR | Bounding | ,0270 | ,2900 | ,0164 | 5,1300 | 1,6463 |
| Luscinia megarhyncho | BR | Bounding | ,0200 | ,2500 | ,0116 | 5,3900 | 1,7241 |
| Megapodius freycinet | UWBM 60186 | Brief continuous flapping | ,8100 | ,7800 | ,1106 | 5,5000 | 7,3237 |
| Megapodius freycinet | UWBM 60327 | Brief continuous flapping | ,8600 | ,7650 | ,1245 | 4,7000 | 6,9076 |
| Megapodius freycinet | UWBM 63063 | Brief continuous flapping | ,5650 | ,7030 | ,1017 | 4,8600 | 5,5556 |
| Melanocorypha mongol | UWBM 57843 | Bounding | ,0582 | ,4130 | ,0274 | 6,2200 | 2,1241 |
| Melanocorypha mongol | UWBM 57846 | Bounding | ,0602 | ,3730 | ,0190 | 7,3200 | 3,1684 |
| Melanocorypha mongol | UWBM 57848 | Bounding | ,0546 | ,3750 | ,0185 | 7,5800 | 2,9514 |
| Melanocorypha mongol | UWBM 59839 | Bounding | ,0463 | ,3660 | ,0219 | 6,1300 | 2,1142 |
| Mergus albellus | VF | Brief continuous flapping | ,4950 | ,6250 | ,0431 | 9,0600 | 11,4849 |
| Mergus merganser | BR | Brief continuous flapping | 1,4790 | ,9600 | ,0680 | 13,5500 | 21,7500 |
| Mergus merganser | VF | Brief continuous flapping | 1,4700 | ,9550 | ,0853 | 10,6900 | 17,2333 |
| Mergus serrator | VF | Brief continuous flapping | ,8180 | ,8860 | ,0589 | 13,3300 | 13,8879 |
| Merops apiaster | UWBM 56655 | Flap-gliding | ,0638 | ,4690 | ,0279 | 7,8900 | 2,2867 |
| Merops apiaster | UWBM 56657 | Flap-gliding | ,0575 | ,4430 | ,0261 | 7,5300 | 2,2031 |
| Merops apiaster | UWBM 56659 | Flap-gliding | ,0570 | ,4450 | ,0270 | 7,3400 | 2,1111 |
| Merops apiaster | BR | Flap-gliding | ,0580 | ,4700 | ,0273 | 8,0900 | 2,1245 |
| Micronisus gabar | BR | Flap-gliding | ,1500 | ,6500 | ,0650 | 6,5000 | 2,3077 |
| Milvus migrans | BR | Flap-gliding | ,8580 | 1,4000 | ,2744 | 7,1400 | 3,1268 |
| Milvus milvus | BR | Flap-gliding | ,8510 | 1,5000 | ,3040 | 7,4000 | 2,7993 |
| Monticola saxatalis | UWBM 60159 | Bounding | ,0453 | ,3610 | ,0224 | 5,8200 | 2,0223 |
| Motacilla alba | BR | Bounding | ,0200 | ,2700 | ,0129 | 5,6500 | 1,5504 |
| Motacilla cinerea | BR | Bounding | ,0170 | ,2600 | ,0110 | 6,1500 | 1,5455 |
| Motacilla flava | BR | Bounding | ,0170 | ,2600 | ,0103 | 6,5600 | 1,6505 |
| Muscicapa striata | BR | Bounding | ,0150 | ,2400 | ,0110 | 5,2400 | 1,3636 |
| Neophron percnopteru | BR | Flap-gliding | 1,8490 | 1,6500 | ,3500 | 7,7800 | 5,2829 |
| Ninox novaeseelandia | UWBM 57397 | Flap-gliding | ,5000 | ,9040 | ,1363 | 6,0000 | 3,6684 |
| Nucifraga caryocatac | BR | Continuous flapping | ,1920 | ,6500 | ,0790 | 5,3500 | 2,4304 |
| Numenius arquata | BR | Continuous flapping | ,7260 | 1,0400 | ,1189 | 9,1000 | 6,1060 |
| Numenius phaeopus | BR | Continuous flapping | ,3730 | ,8200 | ,0730 | 9,2100 | 5,1096 |
| Nyctea scandica | UWBM 76751 | Flap-gliding | 1,5110 | 1,5800 | ,3712 | 6,7200 | 4,0706 |
| Nycticorax nycticora | BR | Continuous flapping | ,6560 | 1,1000 | ,1600 | 7,5600 | 4,1000 |
| Oceanodroma leucorho | VF | Flap-gliding | ,0265 | ,4800 | ,0251 | 9,1800 | 1,0558 |
| Oenanthe oenanthe | BR | Bounding | ,0250 | ,3100 | ,0157 | 6,1200 | 1,5924 |
| Pandion haliaetus | UWBM 79102 | Flap-gliding | 1,0500 | 1,5600 | ,2820 | 8,6300 | 3,7234 |
| Pandion haliaetus | UWBM 89927 | Flap-gliding | 1,3070 | 1,6600 | ,3236 | 8,5100 | 4,0389 |
| Pandion haliaetus | BR | Flap-gliding | 2,0000 | 1,7000 | ,3196 | 9,0400 | 6,2578 |
| Parus ater | BR | Bounding | ,0090 | ,1900 | ,0061 | 5,9200 | 1,4754 |
| Pelecanus onocrotalu | BR | Flap-gliding | 7,3000 | 2,8600 | ,9340 | 8,7600 | 7,8158 |
| Perdix perdix | UWBM 56877 | Brief continuous flapping | ,5300 | ,5220 | ,0463 | 5,8800 | 11,4471 |
| Perdix perdix | UWBM 56882 | Brief continuous flapping | ,3820 | ,5600 | ,0548 | 5,7200 | 6,9708 |
| Perdix perdix | UWBM 58115 | Brief continuous flapping | ,3720 | ,5300 | ,0527 | 5,3300 | 7,0588 |
| Perdix perdix | UWBM 58116 | Brief continuous flapping | ,4040 | ,5240 | ,0491 | 5,6000 | 8,2281 |
| Pernis apivorus | BR | Flap-gliding | ,8000 | 1,2700 | ,2600 | 6,2000 | 3,0769 |
| Phalacrocorax carbo | BR | Brief continuous flapping | 2,5560 | 1,3500 | ,2243 | 8,1300 | 11,3955 |
| Philomachus pugnax F | BR | Continuous flapping | ,1200 | ,4500 | ,0300 | 6,7500 | 4,0000 |
| Philomachus pugnax M | BR | Continuous flapping | ,1900 | ,5700 | ,0450 | 7,2200 | 4,2222 |
| Phoenicopterus ruber | BR | Continuous flapping | 3,0000 | 1,5300 | ,2715 | 8,6200 | 11,0497 |
| Phoenicurus ochruros | BR | Bounding | ,0150 | ,2500 | ,0106 | 5,9000 | 1,4151 |
| Phoenicurus phoenicu | BR | Bounding | ,0150 | ,2300 | ,0106 | 4,9900 | 1,4151 |
| Phylloscopus trochil | BR | Bounding | ,0080 | ,1900 | ,0071 | 5,0800 | 1,1268 |
| Pica pica | UWBM 56707 | Continuous flapping | ,2230 | ,5980 | ,0750 | 4,7700 | 2,9733 |
| Pica pica | UWBM 56890 | Continuous flapping | ,2430 | ,6360 | ,0836 | 4,8400 | 2,9067 |
| Pica pica | UWBM 59758 | Continuous flapping | ,2580 | ,6620 | ,0933 | 4,7000 | 2,7653 |
| Pica pica | UWBM 59759 | Continuous flapping | ,2180 | ,6220 | ,0845 | 4,5800 | 2,5799 |
| Pica pica | UWBM 59899 | Continuous flapping | ,2390 | ,6380 | ,0884 | 4,6100 | 2,7036 |
| Pica pica | UWBM 59930 | Continuous flapping | ,2500 | ,6750 | ,0937 | 4,8600 | 2,6681 |
| Pica pica | UWBM 59931 | Continuous flapping | ,2020 | ,6120 | ,0806 | 4,6500 | 2,5062 |
| Pica pica | BR | Continuous flapping | ,2270 | ,6100 | ,0595 | 6,2500 | 3,8151 |
| Picoides tridactylus | UWBM 56772 | Bounding | ,0665 | ,3770 | ,0278 | 5,1200 | 2,3921 |
| Picoides tridactylus | UWBM 56806 | Bounding | ,0673 | ,3870 | ,0306 | 4,9000 | 2,1993 |
| Picoides tridactylus | UWBM 56807 | Bounding | ,0700 | ,3920 | ,0294 | 5,2200 | 2,3810 |
| Picoides tridactylus | UWBM 56963 | Bounding | ,0720 | ,3900 | ,0296 | 5,1400 | 2,4324 |
| Pitta moluccensis | UWBM 73874 | Bounding | ,0555 | ,3870 | ,0310 | 4,8300 | 1,7903 |
| Pitta moluccensis | UWBM 73875 | Bounding | ,0445 | ,3890 | ,0318 | 4,7500 | 1,3994 |
| Platalea leucorodia | BR | Flap-gliding | 1,5010 | 1,2600 | ,2341 | 6,7800 | 6,4118 |
| Pluvialis apricaria | BR | Continuous flapping | ,1900 | ,5600 | ,0500 | 6,2700 | 3,8000 |
| Pluvialis squatarola | BR | Continuous flapping | ,2100 | ,6000 | ,0550 | 6,5500 | 3,8182 |
| Podargus strigoides | UWBM 62802 | Continuous flapping | ,2980 | ,9350 | ,1798 | 4,8600 | 1,6574 |
| Podargus strigoides | UWBM 62983 | Continuous flapping | ,2230 | ,8300 | ,1333 | 5,1700 | 1,6729 |
| Podargus strigoides | UWBM 79150 | Continuous flapping | ,4300 | ,8950 | ,1469 | 5,4500 | 2,9272 |
| Podargus strigoides | UWBM 57500 | Continuous flapping | ,2520 | ,9920 | ,1746 | 5,6300 | 1,4433 |
| Podiceps auritus | VF | Brief continuous flapping | ,3695 | ,5900 | ,0350 | 9,9500 | 10,5571 |
| Podiceps cristatus | UWBM 56440 | Brief continuous flapping | ,9850 | ,7500 | ,0637 | 8,8300 | 15,4631 |
| Podiceps cristatus | BR | Brief continuous flapping | 1,0000 | ,8100 | ,0855 | 7,6700 | 11,6959 |
| Podiceps cristatus | VF | Brief continuous flapping | ,7900 | ,7860 | ,0561 | 11,0100 | 14,0820 |
| Podiceps griseigna | VF | Brief continuous flapping | ,4800 | ,7200 | ,0542 | 9,5600 | 8,8561 |
| Podilymbus podiceps | VF | Brief continuous flapping | ,3430 | ,6000 | ,0291 | 12,3700 | 11,7869 |
| Porphyrio porphyrio | UWBM 62805 | Brief continuous flapping | ,7490 | ,8400 | ,1177 | 5,9900 | 6,3636 |
| Porphyrio porphyrio | UWBM 63060 | Brief continuous flapping | ,7150 | ,7270 | ,0932 | 5,6700 | 7,6717 |
| Porphyrio porphyrio | UWBM 82829 | Brief continuous flapping | 1,0630 | ,9700 | ,1669 | 5,6400 | 6,3691 |
| Prunella modularis | BR | Bounding | ,0170 | ,2100 | ,0090 | 4,9000 | 1,8889 |
| Psephotus haematonot | UWBM 57446 | Continuous flapping | ,0577 | ,3630 | ,0219 | 6,0200 | 2,6347 |
| Pterocles coronatus | BR | Continuous flapping | ,3000 | ,5700 | ,0432 | 7,5200 | 6,9444 |
| Pterocles orientalis | BR | Continuous flapping | ,4100 | ,7200 | ,0721 | 7,1900 | 5,6865 |
| Ptilinopus regina | UWBM 62874 | Continuous flapping | ,1347 | ,4030 | ,0279 | 5,8200 | 4,8280 |
| Ptilinopus regina | UWBM 62961 | Continuous flapping | ,0982 | ,4070 | ,0280 | 5,9200 | 3,5071 |
| Ptilinopus regina | UWBM 62962 | Continuous flapping | ,1025 | ,4140 | ,0306 | 5,6100 | 3,3497 |
| Ptilinopus regina | UWBM 62976 | Continuous flapping | ,0780 | ,3960 | ,0270 | 5,8100 | 2,8889 |
| Ptyonoprogne rupestr | BR | Flap-gliding | ,0240 | ,3300 | ,0113 | 9,6400 | 2,1239 |
| Puffinus diomedea | VF | Flap-gliding | ,5720 | 1,2110 | ,1280 | 11,4600 | 4,4687 |
| Puffinus pacificus | UWBM 68952 | Flap-gliding | ,3890 | 1,0000 | ,0912 | 10,9700 | 4,2654 |
| Puffinus pacificus | UWBM 68954 | Flap-gliding | ,3640 | ,9500 | ,0968 | 9,3200 | 3,7603 |
| Puffinus pacificus | UWBM 78197 | Flap-gliding | ,2470 | 1,0100 | ,0918 | 11,1200 | 2,6906 |
| Puffinus puffinus | VF | Flap-gliding | ,3420 | ,8110 | ,0575 | 11,4400 | 5,9478 |
| Regulus ignicapillus | BR | Bounding | ,0060 | ,1500 | ,0050 | 4,5000 | 1,2000 |
| Regulus regulus | BR | Bounding | ,0050 | ,1600 | ,0053 | 4,8300 | ,9434 |
| Riparia riparia | BR | Flap-gliding | ,0140 | ,2800 | ,0096 | 8,1700 | 1,4583 |
| Saxicola rubetra | BR | Bounding | ,0160 | ,2400 | ,0095 | 6,0600 | 1,6842 |
| Scolopax minor | UWBM 57803 | Continuous flapping | ,1620 | ,5010 | ,0451 | 5,5700 | 3,5920 |
| Serinus serinus | BR | Bounding | ,0110 | ,2200 | ,0076 | 6,3700 | 1,4474 |
| Stercorarius pomarin | UWBM 59608 | Flap-gliding | ,5710 | 1,1800 | ,1564 | 8,9000 | 3,6509 |
| Streptopelia turtur | BR | Continuous flapping | ,1320 | ,5200 | ,0400 | 6,7600 | 3,3000 |
| Sturnus vulgaris | UWBM 56492 | Bounding | ,0750 | ,3820 | ,0254 | 5,7500 | 2,9528 |
| Sturnus vulgaris | UWBM 56493 | Bounding | ,0690 | ,3900 | ,0251 | 6,0500 | 2,7490 |
| Sturnus vulgaris | UWBM 79098 | Bounding | ,0685 | ,3290 | ,0208 | 5,2000 | 3,2933 |
| Sturnus vulgaris | BR | Bounding | ,0850 | ,3800 | ,0230 | 6,2800 | 3,6957 |
| Sula bassana (=Morus | UWBM 73815 | Flap-gliding | 1,4170 | 1,8600 | ,2587 | 13,3700 | 5,4774 |
| Sula serrator (=Moru | UWBM 63012 | Flap-gliding | 1,3300 | 1,7050 | ,2064 | 14,0900 | 6,4438 |
| Sylvia atricapilla | BR | Bounding | ,0210 | ,2300 | ,0098 | 5,4000 | 2,1429 |
| Sylvia borin | BR | Bounding | ,0170 | ,2300 | ,0095 | 5,5700 | 1,7895 |
| Sylvia communis | BR | Bounding | ,0150 | ,2100 | ,0087 | 5,0700 | 1,7241 |
| Sylvia curruca | BR | Bounding | ,0120 | ,2000 | ,0073 | 5,4800 | 1,6438 |
| Sylvia hortensis | BR | Bounding | ,0210 | ,2400 | ,0112 | 5,1400 | 1,8750 |
| Syrrhaptes paradoxus | UWBM 59831 | Continuous flapping | ,2550 | ,6900 | ,0499 | 9,5300 | 5,1102 |
| Syrrhaptes paradoxus | UWBM 59841 | Continuous flapping | ,2680 | ,6830 | ,0483 | 9,6600 | 5,5487 |
| Tachybaptus ruficoll | VF | Brief continuous flapping | ,1800 | ,4400 | ,0236 | 8,2000 | 7,6271 |
| Tadorna tadorna | UWBM 56439 | Continuous flapping | 1,0000 | 1,0600 | ,1383 | 8,1200 | 7,2307 |
| Tadorna tadorna | UWBM 56451 | Continuous flapping | ,8900 | 1,0400 | ,1337 | 8,0900 | 6,6567 |
| Tadorna tadorna | UWBM 56454 | Continuous flapping | ,9700 | 1,0010 | ,1166 | 8,6000 | 8,3190 |
| Tadorna tadorna | UWBM 59707 | Continuous flapping | ,9500 | 1,0200 | ,1171 | 8,8900 | 8,1127 |
| Tetrax tetrax | UWBM 64702 | Brief continuous flapping | ,7580 | ,8800 | ,1125 | 6,8800 | 6,7378 |
| Trichoglossus haemat | UWBM 62873 | Continuous flapping | ,1152 | ,4460 | ,0300 | 6,6200 | 3,8400 |
| Trichoglossus haemat | UWBM 62876 | Continuous flapping | ,1305 | ,4360 | ,0287 | 6,6300 | 4,5470 |
| Trichoglossus haemat | UWBM 62932 | Continuous flapping | ,1350 | ,4500 | ,0306 | 6,6200 | 4,4118 |
| Tringa glareola | UWBM 56947 | Continuous flapping | ,0590 | ,4100 | ,0220 | 7,6500 | 2,6818 |
| Tringa nebularia | BR | Continuous flapping | ,1710 | ,6000 | ,0369 | 9,7600 | 4,6341 |
| Tringa ochropus | BR | Continuous flapping | ,0870 | ,4500 | ,0289 | 7,0100 | 3,0104 |
| Turdus iliacus | UWBM 56550 | Bounding | ,0610 | ,3560 | ,0224 | 5,6600 | 2,7232 |
| Turdus iliacus | UWBM 56570 | Bounding | ,0575 | ,3510 | ,0220 | 5,6100 | 2,6136 |
| Turdus iliacus | UWBM 59607 | Bounding | ,0700 | ,3630 | ,0232 | 5,6700 | 3,0172 |
| Turdus merula | BR | Bounding | ,0980 | ,3900 | ,0297 | 5,1200 | 3,2997 |
| Turdus philomelos | BR | Bounding | ,0720 | ,3600 | ,0226 | 5,7300 | 3,1858 |
| Turdus pilaris | BR | Bounding | ,0930 | ,4300 | ,0335 | 5,5200 | 2,7761 |
| Turdus ruficollis | UWBM 56784 | Bounding | ,0760 | ,4080 | ,0289 | 5,7700 | 2,6298 |
| Turdus ruficollis | UWBM 58048 | Bounding | ,0794 | ,4020 | ,0283 | 5,7200 | 2,8057 |
| Turdus ruficollis | UWBM 58076 | Bounding | ,0863 | ,4020 | ,0302 | 5,3500 | 2,8576 |
| Turdus ruficollis | UWBM 59994 | Bounding | ,0780 | ,4200 | ,0329 | 5,3600 | 2,3708 |
| Turdus torquatus | BR | Bounding | ,1050 | ,4400 | ,0330 | 5,8700 | 3,1818 |
| Turdus viscivorus | BR | Bounding | ,1350 | ,4700 | ,0358 | 6,1700 | 3,7709 |
| Tyto alba | UWBM 66222 | Flap-gliding | ,4156 | ,9750 | ,1488 | 6,3900 | 2,7930 |
| Tyto alba | UWBM 66223 | Flap-gliding | ,3244 | 1,0460 | ,1433 | 7,6300 | 2,2638 |
| Upupa epops | UWBM 56497 | Continuous flapping | ,0661 | ,4500 | ,0428 | 4,7300 | 1,5444 |
| Upupa epops | UWBM 56678 | Continuous flapping | ,0830 | ,4780 | ,0446 | 5,1200 | 1,8610 |
| Upupa epops | BR | Continuous flapping | ,0700 | ,4400 | ,0422 | 4,5900 | 1,6588 |
| Uria aalge | UWBM 79468 | Brief continuous flapping | ,9250 | ,7220 | ,0613 | 8,5000 | 15,0897 |
| Uria aalge | VF | Brief continuous flapping | 1,0100 | ,7020 | ,0424 | 11,6200 | 23,8208 |
| Uria lomvia | UWBM 80877 | Brief continuous flapping | ,9190 | ,7950 | ,0717 | 8,8100 | 12,8173 |
| Vanellus vanellus | UWBM 56902 | Continuous flapping | ,1970 | ,7550 | ,0945 | 6,0300 | 2,0847 |
| Vanellus vanellus | UWBM 58086 | Continuous flapping | ,2100 | ,7420 | ,0920 | 5,9800 | 2,2826 |
| Vanellus vanellus | UWBM 59865 | Continuous flapping | ,2010 | ,7130 | ,0799 | 6,3600 | 2,5156 |
| Vanellus vanellus | BR | Continuous flapping | ,2360 | ,7300 | ,0820 | 6,5000 | 2,8780 |
