# Supplementary material for: New Bohaiornis-like bird from the Early Cretaceous of China: enantiornithine interrelationships and flight performance
Source: PeerJ. 2019 Oct 25;7:e7846. doi: 10.7717/peerj.7846 (PMC6816414; doi:10.7717/peerj.7846)
Supplement: Supplemental Information 3 — Blue and yellow areas show deviation in the proportions of the carpometacarpus (red = longer; yellow = shorter) with respect to that of Bohaiornis guoi. Note the proportionally much longer carpometacarpus of Gretcheniao sinensis. [file peerj-07-7846-s003.pdf]

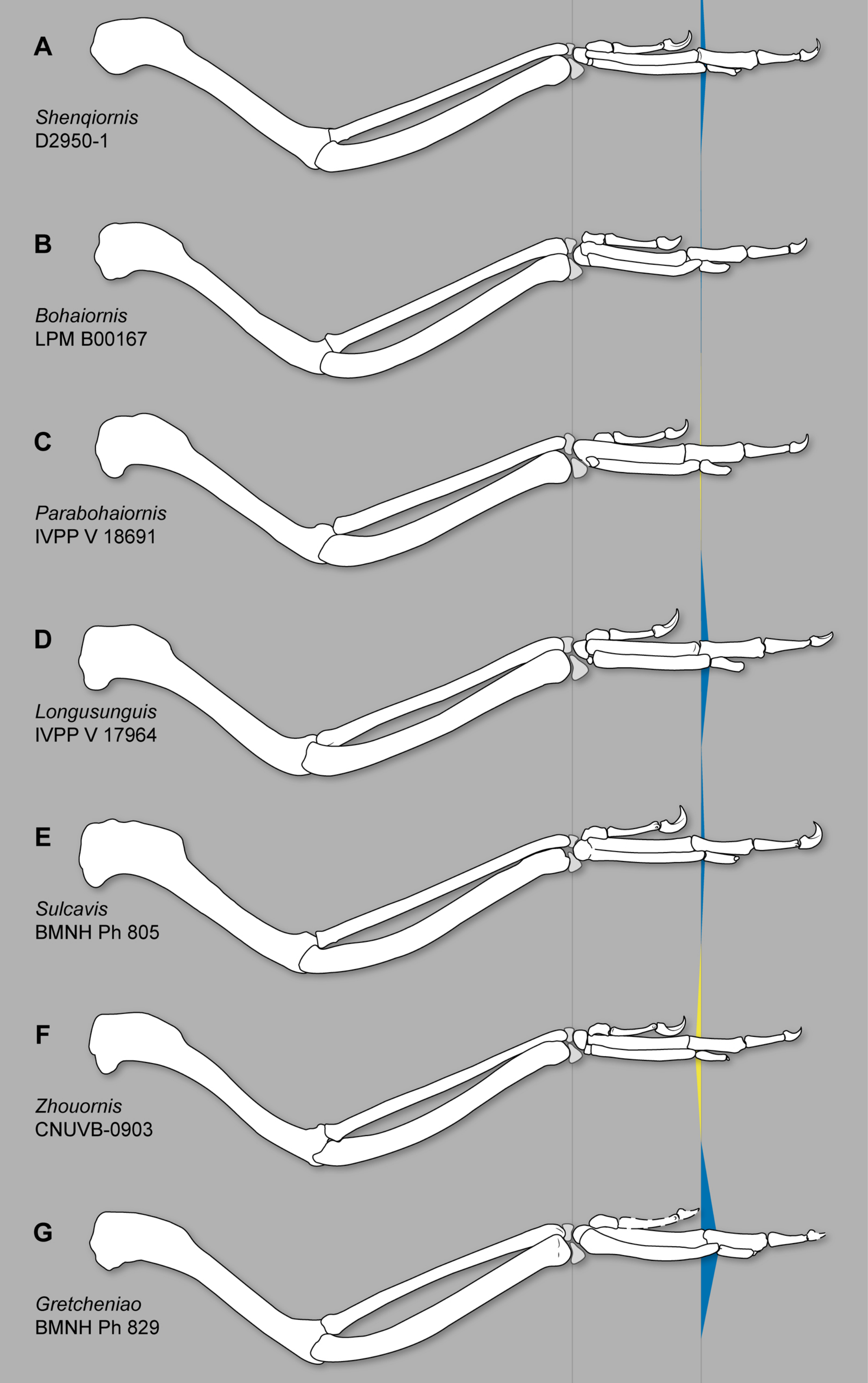
A
Shenqiornis
D2950-1
B
Bohaiornis
LPM B00167
C
Parabohaiornis
IVPP V 18691
D
Longusunguis
IVPP V 17964
E
Sulcavis
BMNH Ph 805
F
Zhouornis
CNUVB-0903
G
Gretcheniao
BMNH Ph 829
